# CSF1R marks a subset of foetal haematopoietic multipotent progenitor cells with acute myeloid leukaemia propagation properties

**DOI:** 10.1038/s41375-025-02856-4

**Published:** 2026-01-16

**Authors:** Giuseppina Camiolo, Daniel González Silvera, Tom Leah, Jürg Schwaller, Katrin Ottersbach

**Affiliations:** 1 Centre for Regenerative Medicine, Institute for Regeneration and Repair, University of Edinburgh, Edinburgh, EH16 4UU UK; 2 University Children Hospital Basel, Department of Biomedicine, University of Basel, Basel, Switzerland

**Keywords:** Cancer stem cells, Acute myeloid leukaemia, Haematopoiesis

## Abstract

KMT2A-rearranged infant leukaemia is one of the most severe malignancies in infants and children, and is characterised by a very aggressive phenotype and lineage plasticity. KMT2A::MLLT3 is among the most common translocations initiating leukaemia in infants, where it can manifest with a myeloid or lymphoid leukaemia phenotype. The cell-of-origin and the mechanisms driving lineage choice in KMT2A::MLLT3+ infant leukaemia are poorly understood. In this study, we show that a subset of foetal lymphoid-primed multipotent progenitors (LMPPs) expressing the Colony-Stimulating Factor 1 receptor (CSF1R) gives rise to acute myeloid leukaemia (AML) upon KMT2A::MLLT3 induction in a mouse model, with the myeloid phenotype, at least in part, being dependent on CSF1R signalling. In line with their leukaemia-propagating properties, KMT2A::MLLT3 + CSF1R+ LMPPs possess a stem cell-like and myeloid-biased expression signature and require autophagy to expand and form blast-like colonies in methylcellulose. Interrogation of public datasets confirms the existence of a human foetal-restricted CSF1R+ LMPP population at early stages of embryonic development. Finally, CSF1R inhibition on a KMT2A::MLLT3+ paediatric leukaemia cell line resulted in significant cell death, suggesting that CSF1R could be therapeutically targeted in these patients. Our findings suggest that KMT2A::MLLT3+ infant AML may originate from foetal liver CSF1R+ LMPPs, and that these patients may benefit from anti-CSF1R-CAR-T cell therapy.

## Introduction

Leukaemia is the most frequent cancer type in children [1]. Over the last two decades, the outcome of childhood leukaemia has dramatically improved, since more efficient therapeutic regimens have been developed [2]. In contrast, infant leukaemia is still characterised by a poor prognosis, particularly those harbouring rearrangements of the *KMT2A* (Lysine methyltransferase 2 A, also known as *MLL*) gene [3]. In haematological malignancies, the *KMT2A* gene can form translocations with more than 100 genes, the most common partner genes of which are *AFF1*, *MLLT3*, and *MLLT1* [4, 5]. Infant *KMT2A*-rearranged (KMT2A-r) leukaemias can manifest as lymphoid (ALL, Acute Lymphoblastic Leukaemia), myeloid (AML, Acute Myeloid Leukaemia), or mixed-lineage leukaemia, and can also undergo lineage switching during treatment or at relapse [6]. While the *KMT2A::MLLT3* translocation is exclusively associated with AML in adult patients, it accounts for 17% of cases with ALL and 22% of cases with AML in infant patients [7], highlighting the high plasticity of these leukaemias in this particular patient group.

An in-utero origin of KMT2A-r leukaemias has been demonstrated by retrospective analyses of blood spot screening tests of newborns, and through shared leukaemic clones in monochorionic twins (reviewed in [8]). Understanding the initiation of infant leukaemia has proven challenging, since haematopoiesis during embryonic life is a dynamic process that takes place in different anatomical sites, namely the yolk sac, the aorta-gonad-mesonephros (AGM) region, foetal liver, and lastly, the bone marrow [9]. Faithful murine models of disease are, therefore, crucial to be able to characterise the mechanisms involved in initiating and sustaining infant KMT2A-r leukaemia, and to identify targets unique to the infant disease that may improve the strategies to treat these vulnerable patients. For the KMT2A::AFF1-associated disease, we [10] and others [11, 12], succeeded in creating models which recapitulate many of the clinical features observed in KMT2A::AFF1+ Pro-B ALL infant patients. The biology of KMT2A::MLLT3+ infant leukaemia, however, is still poorly understood, particularly the identity of the cell-of-origin and the mechanisms that drive the choice between the myeloid and the lymphoid lineage in this subgroup of patients. In fact, all the KMT2A::MLLT3 murine models available to date display an AML phenotype, which is most likely due to the gene expression imposed by the KMT2A::MLLT3 oncoprotein, but also to the more myeloid-biased murine microenvironment [11–13]. Dissecting the mechanisms that determine the choice between ALL and AML would provide much needed insight into the plasticity observed in this disease.

There is growing evidence demonstrating a permissiveness of the foetal context to leukaemia transformation. Foetal-restricted signatures were found to be expressed and maintained in infant leukaemia patients [14, 15]. Within the foetal liver, the population of haematopoietic cells where the KMT2A translocation is most likely to initiate the disease has been suggested to be lymphoid-primed multipotent progenitors (LMPPs) [16]. LMPPs are shown to possess both myeloid and lymphoid lineage potential [17, 18] and they express many of the genes associated with the KMT2A-r signature [19]. Within this population, a subset was identified in the murine foetal liver that expresses the Colony-Stimulating Factor 1 Receptor (CSF1R) and that exhibited higher lineage plasticity compared with CSF1R- LMPPs [20]. The existence of this LMPP subset was confirmed in a recently published single-cell RNA sequencing dataset which shows Csf1r expression in E11.5 Lymphoid-Multipotent Progenitor (LMP) cells [21].

CSF1R has emerged in the last years as a therapeutic target in solid tumours [22], both through expression on tumour cells as well as through marking tumour-associated macrophages that support tumour growth. Similarly, CSF1R-expressing cells in the AML microenvironment were suggested to support the bulk leukaemia population. Exposure of primary AML patient samples in conditioned media to CSF1R chemical inhibitors showed reduced cell viability of leukaemic cells [23]. Indeed, cells expressing high amount of CSF1R retain potent leukaemia-initiating activity, suggesting that leukaemic stem cells are contained within the CSF1R-high expressing cells in AML [24]. Notably, CSF1R was recently identified as a target for CAR-T cell therapy in adult AML. Despite expression of CSF1R on myeloid cells, CSF1R-CAR-T cells were able to clear AML in mice without showing any on-target-off-tumour toxicity [25].

Because of this association of CSF1R with lineage plasticity in foetal progenitors and its potential use as a novel immunotherapy target, we decided to further investigate foetal LMPPs which do or do not express CSF1R. Specifically, our aim was to determine whether in the context of KMT2A::MLLT3-mediated transformation, the lineage choice between ALL or AML could lie in these specific progenitors, and to evaluate the contribution of CSF1R to the initiation of KMT2A::MLLT3+ leukaemia. We show that CSF1R marks a restricted subset of foetal LMPPs, which upon KMT2A::MLLT3 induction, gives rise to AML that recapitulates all the features of this disease. Transcriptionally, these cells possess a myeloid-biased and stem cell-like gene expression signature that seems to endow them with leukaemia stem cell properties. Finally, using public single-cell RNA sequencing datasets of human foetal tissues, we confirm the foetal-restricted expression of CSF1R in human LMPPs at a very early stage of embryonic development, suggesting that these cells may serve as the cell-of-origin for infant AML.

## Methods

### Cell lines

THP-1 (ATCC TIB-202), NOMO-1 (DSMZ ACC 542) were kindly provided by Brian Huntly (University of Cambridge). Cells were maintained in 20% foetal calf serum (FCS) in RPMI 1640 containing 1% penicillin/streptomycin (P/S) and 1% l-glutamine. Cytogenetic for both cell lines is KMT2A::MLLT3+ . MS5 cells were kindly provided by Elisa Laurenti (University of Cambridge).

### Apoptosis assay

Apoptotic cells were detected by double staining with PE-annexin V (640907; Biolegend) and DAPI (62248; ThermoFisher) in annexin V binding buffer, according to the manufacturer’s instructions (556454; BD Biosciences). Data were acquired on an LSRFortessa (BD Biosciences).

### Mice

All animal work was carried out under the regulation of the UK Home Office and following local ethical review. Males and females from an inducible rtTA-KMT2A::MLLT3 transgenic line [13] were mated to obtain E14.5 embryos, with the day of plug detection being counted as day 0 of embryonic development. Pregnant females were given Doxycycline (VWR INTERNATIONAL LTD, cat# J60579.22) at 1 mg/ml with 5% glucose (MERCK LIFE SCIENCE, cat# 1076511000) in the drinking water from E12.5 to induce KMT2A::MLLT3 expression in embryos. For the transplants, NOD/SCID/IL2rγ^null^ (NSG) mice were used as recipients, with a mixture of both males and females that were randomly allocated to groups. Test cells injected along with total bone marrow helper cells were from CD45.1/.2 mice.

### Genotyping

Transgenic embryos were identified by PCR using the following primers: *HPRT* (*KMT2A::MLLT3)* Fw: CTAGATCTCGAAGGATCTGGAG; *HPRT* (*KMT2A::MLLT3*) Rv: ATACTTTCTCGGCAGGAGCA; rtTA (*ROSA26*) wildtype: AAAGTCGCTCTGAGTTGTTAT; rtTA common: GCGAAGAGTTTGTCCTCAACC; rtTA mut: GGAGCGGGAGAAATGGATATG. PCR reactions were run on a Peltier Thermal Cycler (cycling conditions in Supplementary Table S1). The expected band sizes are 400–500 bp for the *KMT2A::MLLT3* gene, 650 bp for the wildtype *ROSA* allele, and 450 bp for the *rtTA* insertion.

### Sorting of murine foetal liver LMPPs

Foetal livers were harvested at E14.5 and dissociated in Flow Cytometry Staining Buffer (PBS supplemented with 2% FCS and 1% P/S) using a 21Gx15mm needle attached to a syringe (BD Microlance Cat# 10472204-X and 3000185). Cell pellets were washed in Flow Cytometry Staining Buffer and stained with the following antibodies: for the lineage cocktail on APC: APC-CD3e antibody (clone I45-2C11, Biolegend, cat# 100312), APC-Ter119 antibody (clone TER-119, Biolegend Cat# 116212), APC-B220 antibody (clone RA3-6B2, ThermoFisher Cat# 17-0452-81), APC-F4/80 antibody (clone BM8, Biolegend cat# 123116), APC-Gr1 antibody (clone RB6-8C5, Biolegend, cat# 20-5931-U100), APC-NK1.1 antibody (clone PK136, Biolegend, Cat# 108710), APC-CD19 antibody (clone 6D5, Biolegend cat# 115512), PE-CD127 (IL7R) antibody (clone ATR34, ThermoFisher, Cat# 12-1271-82), PB-Sca1 antibody (clone E13-161.7, Biolegend, Cat# 122520), APCeF780-ckit antibody (clone 2B8, Thermo Fisher, Cat# 47-1171-82), Alexa700-CD45 antibody (clone 30-F11, Biolegend, Cat# 103128), biotin-CD135 (Flt3) antibody (clone A2F10, Biolegend, Cat# 13-1351-82), Qdot655 streptavidin-conjugate (ThermoFisher, Cat# Q10121MP), PEDazzle594-CSF1R antibody (clone I45-2C11, Biolegend, Cat# 135527). Cells were incubated on ice for 20 min, washed twice with Flow Cytometry Staining Buffer and resuspended in diluted SYTOX RED (ThermoFisher Cat# S34855) to exclude dead cells. Cells were sorted on a BD FACSAriaTM II (BD Biosciences) and used for in vitro functional assays, transplants and RNA-seq.

### Colony-forming assays

For myeloid assays, cells were plated in M3434 Methocult (STEMCELL Technologies, cat# 3434) in technical triplicates for each biological replicate (number of replicates are stated in the figure legends) and colonies were scored and counted after 7 days. For lymphoid assays, cells were plated in M3630 Methocult (STEMCELL Technologies, cat# 3630) supplemented with 20 ng/ml SCF (PeproTech, cat# 250-03-10uG) and 20 ng/ml Flt3 (PeproTech, cat# 250-31L-10uG) in triplicate at different concentrations, and colonies were counted after 14 days. As previously established in the lab, the different types of B lymphoid colonies were distinguished by morphology and size, with T1 being diffuse and <1mm^2^, T2 being more compact and also <1mm^2^, while T3 are >1mm^2^. Where stated, Doxycycline (1ug/ml), GW2580 (10uM), and Chloroquine (10uM) were added to the methylcellulose medium at the indicated concentrations.

### Transplantation of mouse KMT2A::MLLT3+ CSF1R- / CSF1R+ LMPP cells into NSG mice and flow cytometry analysis of recipients

On the day of the transplantation, NSG mice (aged 8–12 weeks old, CD45.1/.1, mixture of males and females randomly assigned to each group) were irradiated with a total dose of 2 Gy (2 doses of 1 Gy, 3 h apart, with a split adaptor). Freshly sorted KMT2A::MLLT3+ CSF1R- and KMT2A::MLLT3+ CSF1R+ LMPP cells (CD45.2/.2) were transplanted through tail-vein injection (2000 cells/mouse) along with 20,000 helper bone marrow cells (CD45.1/.2). Mice were administered antibiotics after transplantation through their drinking water (0.1 mg/mL Enrofloxacin, 10% Baytril solution from Bayer) for 1 month and Doxycycline 400 µg/ml + 5% glucose through their drinking water to keep KMT2A::MLLT3 expression for the whole duration of the experiment. Mice were bled on a monthly basis. For flow cytometry analysis of peripheral blood and tissues, red blood cell lysis was carried out with BD Pharm Lyse™ solution according to the manufacturer’s instructions (BD Biosciences Cat# 555899). Cells were stained in Flow Cytometry Staining Buffer using the following mixture of antibodies: FITC-CD45.2 monoclonal antibody (clone 104, BD Cat# 553772), APC-CD45.1 antibody (clone A20, Biolegend Cat# 110714), BUV395-CD11b antibody (clone M1/70, BD, Cat# 563553), Alexa Fluor® 700-CD24 antibody (clone M1/69, Biolegend Cat# 101836), PE/Cy7-CD45R/B220 antibody (clone RA3-6B2, Biolegend Cat# 103222), Brilliant Violet 605™-CD19 antibody (clone 6D5, Biolegend Cat# 115539), PECy5-IgM antibody (clone II/41, Invitrogen Cat# 2020-11-09), BV875-CD19 antibody (clone 6D5, Biolegend, cat# 115543), PE-ckit (CD117) antibody (clone 2B8, Biolegend, Cat# 105807), APC-eFluor 780-ckit monoclonal antibody (clone 2B8, Life Technologies, cat# 47-1171-82), PE-CD127 (IL7R) monoclonal antibody (clone A7T34, ThermoFisher Cat# 12-1271-82), PerCP-CD43 antibody (clone 1B11, Biolegend, cat# 121222). Cells were incubated on ice for 20 min, washed twice with Flow Cytometry Staining Buffer and resuspended in diluted DAPI (Invitrogen, Cat# D1306) to exclude dead cells. Data were acquired on a BD LSRFortessa^TM^ (BD Biosciences).

For serial transplants, total bone marrow cells from primary recipients which received KMT2A::MLLT3+ CSF1R+ and KMT2A::MLLT3+ CSF1R- LMPPs were thawed and injected through tail vein injection into irradiated NSG mice (200,000 cells/mouse) as above. Mice were administered Doxycycline 400 µg/ml + 5% glucose through their drinking water for the whole duration of the experiment. Mice were bled monthly and blood and tissues analysed for engraftment as above.

### Histology

Tissues from diseased mice were fixed in formalin, dehydrated, and embedded in wax. Sections were prepared and stained with H&E, dehydrated, and mounted in DePeX-mounting medium (Fisher Scientific). Pictures were taken on a EVOS M5000 Observer (Invitrogen), and on a Zeiss Observer, using the ZEN software,

### Cocultures of MS5 cells with KMT2A::MLLT3+ CSF1R+ or KMT2A::MLLT3+ CSF1R- LMPPs

MS5 stromal cells (40,000/48 well-plate) were seeded 24 h prior starting the coculture in complete α-MEM (10% FCS, 1% P/S, 1% Glutamate, 10 mM HEPES, 50 nM 2β-mercaptoethanol). Sorted LMPPs were collected in complete α-MEM and added on to the stroma layer (2000/48 well-plate), in a final volume of 1 mL/well. Where stated, 50 ng/mL IL7 (cat# 17-17-10uG, PEPROTECH EC LTD), 10 µM GW2580, a chemical inhibitor of CSF1R (SELLECK CHEMICALS GMBH, cat# S8042) and Doxycycline at 1 µg/mL were added to the coculture media. At day 7, cells were harvested and stained in Flow Cytometry Staining Buffer using the following mixture of antibodies: FITC-CD45.2 monoclonal antibody (clone 104, BD Cat# 553772), BUV395-CD11b antibody (clone M1/70, BD, Cat# 563553), PE/Cy7-CD45R/B220 antibody (clone RA3-6B2, Biolegend Cat# 103222), APC-eFluor 780-ckit monoclonal antibody (clone 2B8, Life Technologies, cat# 47-1171-82). Cells were incubated on ice for 20 min, washed twice with Flow Cytometry Staining Buffer and resuspended in diluted DAPI (Invitrogen, Cat# D1306) to exclude dead cells. Data were acquired on a BD LSRFortessa^TM^ (BD Biosciences).

### Bulk RNA-sequencing

#### RNA extraction

RNA extraction from sorted KMT2A::MLLT3+ CSF1R+ ( ~ 3000 cells per biological replicate) and from KMT2A::MLLT3+ CSF1R- ( ~ 6000 cells per biological replicate) LMPPs was carried out using RNeasy Plus Micro Kit (cat# 74034, Qiagen), according to the manufacturer’s instructions.

#### Quality control

Total RNA from 4 biological replicates each of KMT2A::MLLT3+ CSF1R+ and KMT2A::MLLT3+ CSF1R- LMPPs was assessed on the Agilent 2100 Electrophoresis Bioanalyser Instrument (Agilent Technologies Inc, #G2939AA) and RNA 6000 Pico chips (#5067-1513) for quantity, quality, and integrity of total RNA. One of the replicates of KMT2A::MLLT3+ CSF1R- LMPPs showed poor sequencing quality and was therefore excluded from all further analysis.

#### Library preparation

Eight Libraries were prepared from 0.5 ng of total RNA samples using the NEBNext® Single Cell/Low Input RNA Library Prep Kit for Illumina® (NEB #E6420) according to the provided protocol. The kit uses a template switching method to generate full length cDNAs directly from single cells or 2 pg – 200 ng RNA, followed by conversion to sequence-ready libraries using the Ultra™ II FS workflow.

Total RNA samples were annealed with cDNA primers before reverse transcription and template switching. cDNA samples were then amplified for 13 cycles of PCR. Amplified cDNA was purified with Agencourt AMPure XP beads (Beckman Coulter, #A63881) and quality and quantity were assessed on the Agilent Bioanalyser with the DNA HS Kit (#5067-4626).

All samples went into the Fragmentation step undiluted. Fragmentation and end-prep of the cDNA was performed in a single enzymatic reaction. During this step, a single ‘A’ nucleotide was added to the 3’ ends of the blunt fragments to prevent them from ligating to another during the subsequent adapter ligation reaction, and a corresponding single ‘T’ nucleotide on the 3’ end of the adapter provided a complementary overhang for ligating the adapter to the fragment. Multiple unique dual indexing adapters were then ligated to the ends of the double-stranded cDNA to prepare them for hybridisation onto a flow cell, before 12 cycles of PCR were used to selectively enrich those DNA fragments that had adapter molecules on both ends and amplify the amount of DNA in the library suitable for sequencing. After amplification, libraries were purified using AMPure XP beads.

#### Library QC

Libraries were quantified by fluorometry using the Qubit dsDNA HS assay and assessed for quality and fragment size using the Agilent Bioanalyser with the DNA HS Kit (#5067-4626). Fragment size and quantity measurements were used to calculate molarity for each library pool.

#### Sequencing

Sequencing was performed on the NextSeq 2000 platform (Illumina Inc, #20038897) using NextSeq 1000/2000 P2 Reagents (200 Cycles) v3 (#20046812). Libraries were combined in an equimolar pool of 6 based on Qubit and Bioanalyser assay results and run over a P2 flow cell. PhiX Control v3 (Illumina Inc, #FC-110-3001) was spiked into each run at a concentration of 1%.

### Data analysis and statistical tests

For the transcriptomic analysis, fastq files quality control was done using FastQC 0.11.9. Reads were trimmed with TrimGalore 0.6.6 and aligned to the reference genome (GRCm39; GCA_000001635.9) with STAR/2.7.1a. Aligned reads were then analysed in RStudio (2022.07.2 + 576 version). The following packages were used to generate the data plots: DESeq2 [26], ggplot2 (10.1007/978-3-319-24277-4_9), ComplexHeatmap [27], ClusterProfiler [28], EnhancedVolcano ( < https://bioconductor.org/packages/EnhancedVolcano), Org.Mm.eg.db (M C. _org.Mm.eg.db: Genome wide annotation for Mouse_. R package version 3.17.0), AnnotationDbi (https://bioconductor.org/packages/AnnotationDbi), tidyverse (10.21105/joss.01686). For GO (Gene Ontology) terms DAVID [29] and ShinyGO v0.77 [30] software were used. GSEA (Gene Set Enrichment Analysis) was carried out using GSEA 4.4.0 [31, 32] EnrichR [33, 34]. Data analysis of CD84, SNAP29, SXT17, ULK, ULK2, ULK3, and CSF1R, in AML with t(11q23)/*KMT2A* rearrangements, AML with t(5;17), AML with t(8;21)patients and healthy bone marrow (HBM) from the Leukaemia MILE study (GSE13159) were obtained from BloodSpot [35]. Graphs were generated using GraphPad Prism 8 (GraphPad software corporation). Normal distribution of data points was evaluated using the Shapiro–Wilk test. Parametric tests (t-test and ANOVA) were applied to samples that passed the Shapiro-Wilk test (p > 0.05), while the Mann–Whitney U test was used for non-normal data. Statistical tests are specified in the figure legends.

### Data accessibility

The RNA-Seq data were deposited in NCBI Gene Expression Omnibus (GEO) under accession number GSE277342.

## Results

### KMT2A::MLLT3+ CSF1R+ LMPPs have a higher clonogenic potential and are more promiscuous than KMT2A::MLLT3+ CSF1R- LMPPs

**Fig. 1:**
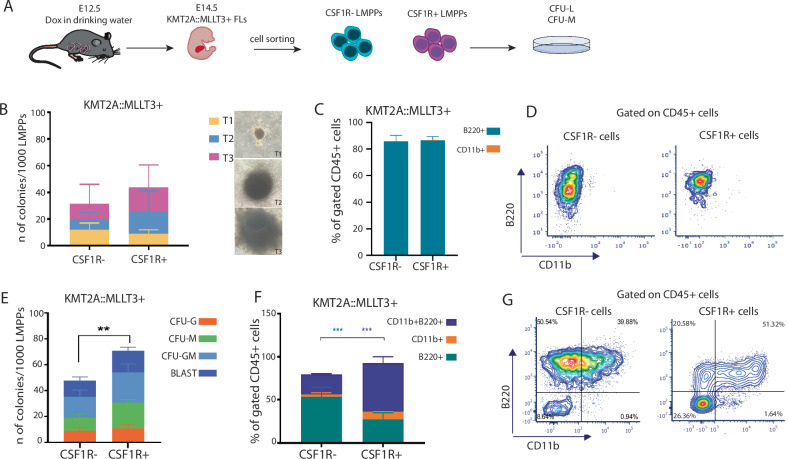
KMT2A::MLLT3+ CSF1R+ LMPPs have a higher clonogenic potential and are more promiscuous than KMT2A::MLLT3+ CSF1R- LMPPs. **A** Experimental layout. **B** Type 1 (T1), Type 2 (T2), and Type 3 (T3) colony output of CSF1R + /- KMT2A::MLLT3+ LMPPs in CFU-lymphoid assays (*N* = 4, from 2 experiments/litters); right panel showing representative pictures of Type 1 (T1), Type 2 (T2), Type 3 (T3) colonies. All pictures were acquired at 4X magnification. **C** Phenotype of CSF1R- and CSF1R+ colonies at the end of the CFU-lymphoid assay. Data shown as percent of B220+ lymphoid, CD11b+ myeloid cells in the CD45+ population (*N* = 3). **D** Representative flow cytometry plots showing the cell phenotype of CSF1R- and CSF1R+ colonies at the end of the CFU-lymphoid assay. **E** BLAST, CFU-GM, CFU-M, CFU-G colony output of CSF1R + /- KMT2A::MLLT3+ LMPPs in CFU-myeloid assay (*N* = 3, from 2 experiments/litters). **F** Phenotype of CSF1R- and CSF1R+ colonies at the end of the CFU-myeloid assay. Data shown as percent of B220+ lymphoid, CD11b+ myeloid and B220 + CD11b+ mixed-phenotype cells in the CD45+ population. **G** Representative flow cytometry plots showing the cell phenotype of CSF1R- and CSF1R+ colonies at the end of the CFU-myeloid assay (N = 3). Data are presented as mean with SD, and statistical analysis was calculated with 2-way ANOVA test: *p* < 0.01 (**) *p* < 0.001 (***), *p* < 0.0001 (****). CFU-GM (colony-forming unit-granulocytes/macrophages), CFU-M (colony-forming unit macrophages), CFU-G (colony-forming unit granulocytes).

To understand whether KMT2A::MLLT3+ LMPPs with or without CSF1R expression possess different lineage biases, we first performed colony-forming assays (in both myeloid and lymphoid conditions) on freshly sorted KMT2A::MLLT3+ CSF1R+ and CSF1R- LMPPs from E14.5 foetal liver (Fig. 1A). We found that KMT2A::MLLT3+ CSF1R+ LMPPs showed a trend toward a higher colony number output when kept under lymphoid conditions, especially of Type 3 (T3) colonies, which represent the most transformed colony type [16]; however, this did not reach significance (Fig. 1B). Analysis of the cell phenotype within colonies confirmed lymphoid output from both CSF1R- and CSF1R+ cells, with cells being uniformly B220+ (Fig. 1C,D).

Under myeloid conditions, we observed a similar colony-type output from both populations, but the number of total colonies was significantly higher from CSF1R+ cells (*p* < 0.001) (Fig. 1E). Interestingly, we observed cells within these colonies with an abnormal mixed phenotype (CD11b+ B220+ ) the percentage of which was significantly higher in CSF1R+ colonies (*** *p* = 0.006), whereas the phenotype of the CSF1R- colonies was represented by more B220+ lymphoid-biased cells (*** *p* = 0.005) (Fig. 1F,G).

### KMT2A::MLLT3+ CSF1R+ LMPPs have AML-propagating potential

**Fig. 2:**
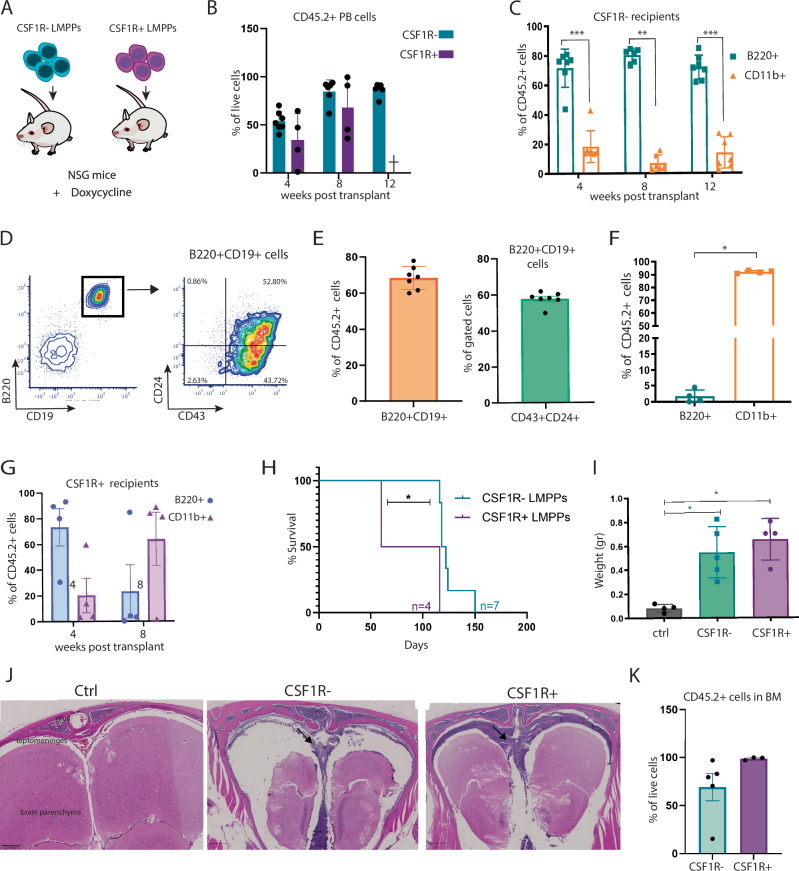
KMT2A::MLLT3+ CSF1R+ LMPPs give rise to AML in vivo. **A** Experimental design for the in vivo studies. Freshly sorted CSF1R- KMT2A::MLLT3+ LMPPs and CSF1R+ KMT2A::MLLT3+ LMPPs were injected via tail-vein into NSG mice (CSF1R- LMPPs, *n* = 7 recipients; CSF1R+ LMPPs *n* = 4 recipients). **B** Peripheral blood reconstitution of NSG recipients injected with CSF1R+ /- KMT2A::MLLT3+ LMPPs. Blood was analysed monthly by flow cytometry, as percentage of CD45.2+ engrafted cells in the live population. 12-week timepoint for CSF1R+ mice was not analysed since mice had already succumbed to leukaemia. **C** Peripheral blood reconstitution of CSF1R- recipients, assessed by flow cytometry as percentage of B220+ and CD11b+ cells within CD45.2+ cells. **D** Representative flow cytometry plots of Pro-B phenotype of engrafted cells from CSF1R- recipient at week 12 post-transplant. **E** Pro-B phenotype of peripheral blood from CSF1R- recipients, assessed by flow cytometry as percentage of engrafted B220+ CD19+ CD43+ CD24+ cells within CD45.2+ cells at week 12 post-transplant. **F** Phenotype of peripheral blood donor cells in NSG recipients injected with KMT2A::MLLT3+ CSF1R- LMPPs (*n* = 4) at the end point of the experiment (120–150 days post-transplant), assessed by flow cytometry as percentage of engrafted B220+ and CD11b+ cells within the CD45.2+ population. **G** Peripheral blood reconstitution of NSG recipients injected with KMT2A::MLLT3+ CSF1R+ LMPPs. Blood was analysed monthly by flow cytometry, as a percentage of CD45.2+ B220+ , and CD45.2+ CD11b+ engrafted cells in the live population. The 12-week timepoint was not analysed since the mice had already succumbed to leukaemia. **H** Survival curve of NSG primary recipients of CSF1R- LMPPs (*n* = 7) and CSF1R+ LMPPs (*n* = 4) (both KMT2A::MLLT3+ ). **I** Spleen weights in control (ctrl, *n* = 4), CSF1R- LMPPs (*n* = 5) and CSF1R+ LMPPs (*n* = 4) mice (ctrl mice were injected with vehicle solution: PBS + 2% FCS 1% P/S). **J** Representative picture of CNS histological sections of control (CSF1R- recipient that did not develop disease; left image) and leukaemia infiltration in CSF1R- and CSF1R+ primary recipients (scale bar 200 µm, 500 µm). **K** Bone marrow engraftment of NSG recipients injected with CSF1R+ /- KMT2A::MLLT3+ LMPPs. Blood was analysed at the end point of the experiment by flow cytometry, as percentage of CD45.2+ engrafted cells in the live population (CSF1R+ *n* = 3; CSF1R- *n* = 5). Survival differences were analysed with the Gehan-Breslow-Wilcoxon test. Data are presented as mean with SD, and statistical analysis was calculated with Mann-Whitney U test: *p* = 0.0006 (***), *p* = 0.0022 (**), *p* = 0.0286 (*). CNS (central nervous system).

Next, we assessed the lineage output of KMT2A::MLLT3+ CSF1R+ /- LMPPs in vivo. As KMT2A::MLLT3 has so far exclusively induced an AML phenotype in mice [36, 37], we used NSG mice as recipients, as they are more likely to promote B-cell reconstitution of donor cells [38] (Fig. 2A). As previously observed with KMT2A::AFF1 [19], the expression of KMT2A::MLLT3 conferred repopulating potential to LMPPs (Fig. 2B). Strikingly, we observed, for the first time, that the donor reconstitution of NSG receiving KMT2A::MLLT3+ CSF1R- LMPPs was primarily composed of B220+ lymphoid cells, with the majority of these displaying a pro-B cell phenotype (B220+ CD19+ CD43+ CD24+ ), which is the stage at which differentiation arrests in infant patients with Pro-B ALL (Fig. 2D,E). This was maintained for the first 3 months post-injection, but switched to a myeloid phenotype at the end point of the experiment, when the mice got sick between 120 and 150 days post-transplant (Fig. 2F,H).

Reconstitution of recipients injected with KMT2A::MLLT3+ CSF1R + LMPPs was mainly lymphoid at 30 days post-transplant, but converted to myeloid at the next sampling stage of 8 weeks (Fig. 2G). Interestingly, NSG mice which received KMT2A::MLLT3+ CSF1R+ LMPP cells had a shorter disease progression than those injected with CSF1R- cells (Fig. 2H). Recipients of both populations showed significant spleen enlargement (CSF1R- LMPPs recipients *p = 0.0286, CSF1R+ LMPPs recipients *p = 0.0286) at the end point of the experiment, compared to control NSG mice (Fig. 2I), and leukaemia infiltration (black arrow) in the central nervous system (CNS) (Fig. 2J).

**Fig. 3:**
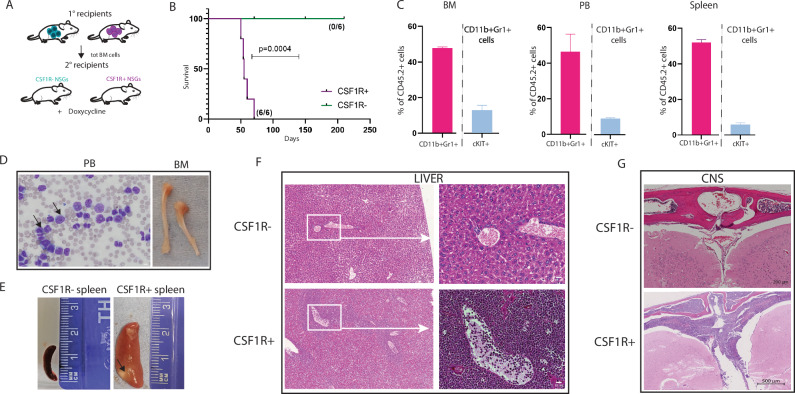
KMT2A::MLLT3+ CSF1R+ LMPPs have AML-propagating properties. **A** Experimental layout of secondary transplants. 200.000 total bone marrow cells from CSF1R- primary recipients (*n* = 2) and CSF1R+ primary recipients (*n* = 3) were intravenously injected into NSG mice (6 recipients each). CSF1R- donor cells failed to engraft secondary recipients, while all CSF1R+ recipients showed similar chimerism. **B** Survival curve of NSG secondary recipients that received total bone marrow cells from CSF1R- and CSF1R+ primary recipients. **C** Percentages of donor CD11b+ Gr1+ and CD11b+ Gr1+ cKIT+ cells in the bone marrow, peripheral blood, and spleens of CSF1R+ secondary recipients. Percentages of CD11b+ Gr1+ cells are shown within CD45.2+ cells; percentages of cKIT+ cells are shown within CD11b+ Gr1+ cells. **D** Representative picture of May-Grünwald Giemsa staining of peripheral blood smear (PB, left) and pale bones (BM, right) from secondary CSF1R+ NSG recipients. Black arrow indicates the presence of myeloblast cells; scale bar = 0.5 µm. **E** Representative picture of spleen enlargement and leukaemia infiltration (black arrow) in secondary CSF1R+ NSG recipients. **F** Liver histological sections (Haematoxylin-Eosin staining) of CSF1R- secondary recipients, and leukaemic liver from secondary CSF1R+ NSG recipients (left panel scale bar = 200 µm, right panel scale bar = 20 µm). White box area shown at higher magnification on the right. **G** Representative picture of CNS histological sections of leukaemia infiltration in CSF1R+ secondary recipients (lower panel) compared to CSF1R- secondary recipients (upper panel); scale bar 500 µm. Survival differences were analysed with the Gehan-Breslow-Wilcoxon test. CNS (central nervous system).

We then harvested total bone marrow cells (which at this point contained ≥ 70% leukaemia cells in CSF1R- mice, and > 90% leukaemia cells in CSF1R+ mice) from primary recipients (Fig. 2K) and carried out secondary transplantations into NSG mice (Fig. 3A). Surprisingly, only cells from CSF1R+ primary recipients were able to engraft secondary recipients, with a shortened disease latency of 54 ± 13 days (Fig. 3B). Flow cytometry analysis of mice at the end point of the experiment confirmed the presence of myeloid cells (CD11b+ Gr1+ ) in peripheral blood (PB), bone marrow and spleen. A subset of these also co-expressed cKIT (9.00% of CD11b+ Gr1+ cells in PB, 12.82% of CD11b+ Gr1+ cells in BM, and 5.84% of CD11b+ Gr1+ cells in spleen) (Fig. 3C). The presence of myeloblast cells in these recipients was confirmed by morphological analysis of PB (Fig. 3D, black arrow). Moreover, post-mortem analysis of diseased mice revealed signs of leukaemia represented by pale long bones (Fig. 3D, BM) and spleen enlargement with visible infiltration of leukaemic cells (Fig. 3E, black arrow). Myeloblast infiltration was also found around the portal liver veins of sick mice (Fig. 3F). Leukaemia-infiltrating cells were also found in the CNS of CSF1R+ secondary recipients (Fig. 3G). Overall, these data identified KMT2A::MLLT3+ CSF1R+ LMPPs as an AML-inducing and propagating subset of LMPPs.

### The transcriptome of KMT2A::MLLT3+ CSF1R+ LMPPs is enriched for stem cell-like and HSC self renewal-related processes

**Fig. 4:**
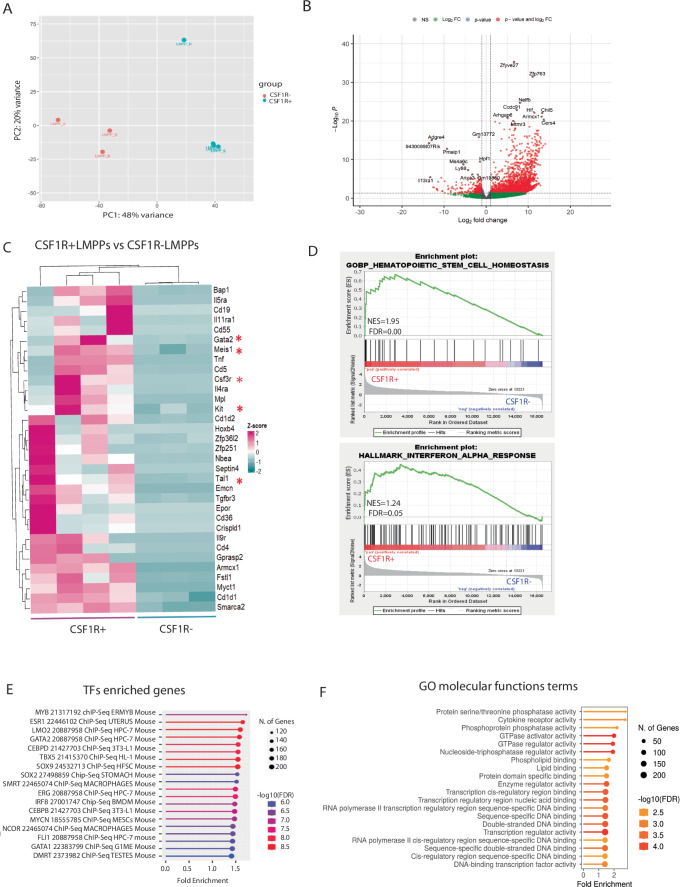
The Transcriptome of KMT2A::MLLT3+ CSF1R+ LMPPs is enriched for stem cell-like and HSC self renewal-related processes. **A** Principal component analysis (PCA) of all 7 samples. **B** Volcano plot showing all 2400 differentially expressed genes between the two populations (log2-fold-change threshold = 1, corrected p-value threshold = 0.05). Top 10 upregulated genes were: *Zfyve27, Zfp763, Nelfb, Ccdc91, Hlf, Chil5, Armcx1, Arhgap6, Cers4, Mtmr3*. Top 10 downregulated genes were: *Adgre4, 9430069I07Rik, Pmaip1, Ms4a6c, Hpf1, Ly86, Il13ra1, Anxa3, Gm13772, Gm19880*. **C** Heatmap of the most overrepresented haematopoiesis-associated genes (red asterisks indicate upregulated genes in CSF1R+ LMPPs associated with definitive haematopoiesis) in KMT2A::MLLT3+ CSF1R+ LMPPs versus KMT2A::MLLT3+ CSF1R- LMPPs; gene list obtained from DAVID. **D** GSEA of genes overrepresented in KMT2A::MLLT3+ CSF1R+ LMPPs versus KMT2A::MLLT3+ CSF1R- LMPPs. Upper panel indicates enriched genes for haematopoietic stem cell homoeostasis, lower panel indicated enriched genes for IFNγ response. NES= normalised enrichment score; FDR= false discovery rate. **E** Enrichment for transcription factors (TFs)/target genes obtained from ChIP Enrichment Analysis (ChEA) 2016 dataset (FDR = 0.05), data obtained from ShinyGO 0.77. **F** GO terms for molecular functions of DEGs KMT2A::MLLT3+ CSF1R+ LMPPs.

To gain better insight into the differences between KMT2A::MLLT3+ CSF1R+ and CSF1R- LMPPs, we performed bulk RNA-sequencing on sorted E14.5 foetal liver populations. Principal Component Analysis (PCA) separated the 2 populations into different clusters (Fig. 4A). Differential gene expression analysis with a threshold of a two-fold change identified 2297 differentially expressed genes (DEGs) with 307 genes found downregulated (Supplementary Table 2) and 1990 upregulated (Supplementary Table 3) in KMT2A::MLLT3+ CSF1R+ LMPPs (Fig. 4B). To further characterise the DEGs, we analysed the gene ontology (GO) annotations associated with our dataset. We found genes associated with haematopoietic homoeostasis and definitive haematopoiesis being upregulated in KMT2A::MLLT3+ CSF1R+ LMPPs, such as *Gata2, Meis1, Kit, Csf3r, Tal1* (Fig. 4C, red asterisks). Gene set enrichment analysis (GSEA) confirmed enrichment for a haematopoietic cell stem (HSC) homoeostasis signature in KMT2A::MLLT3+ CSF1R+ LMPPs (Fig. 4D upper panel). Furthermore, using the ChEA2016 database [39], our data showed enriched target genes for transcription factors associated with HSC renewal and stemness like *Gata2, Mecom, Meis1* and *Erg* (Fig. 4E). Notably, analysis of GO terms for molecular functions revealed enrichment in pathways associated with transcription regulatory activities, RNA polymerase II transcription, DNA-binding transcription activity (Fig. 4F), altogether suggesting that the transcriptional profile of KMT2A::MLLT3+ CSF1R+ LMPPs resembles that of a more undifferentiated haematopoietic cell population, with a stem cell-like signature.

### The KMT2A::MLLT3+ CSF1R+ LMPP transcriptome is AML-biased

**Fig. 5:**
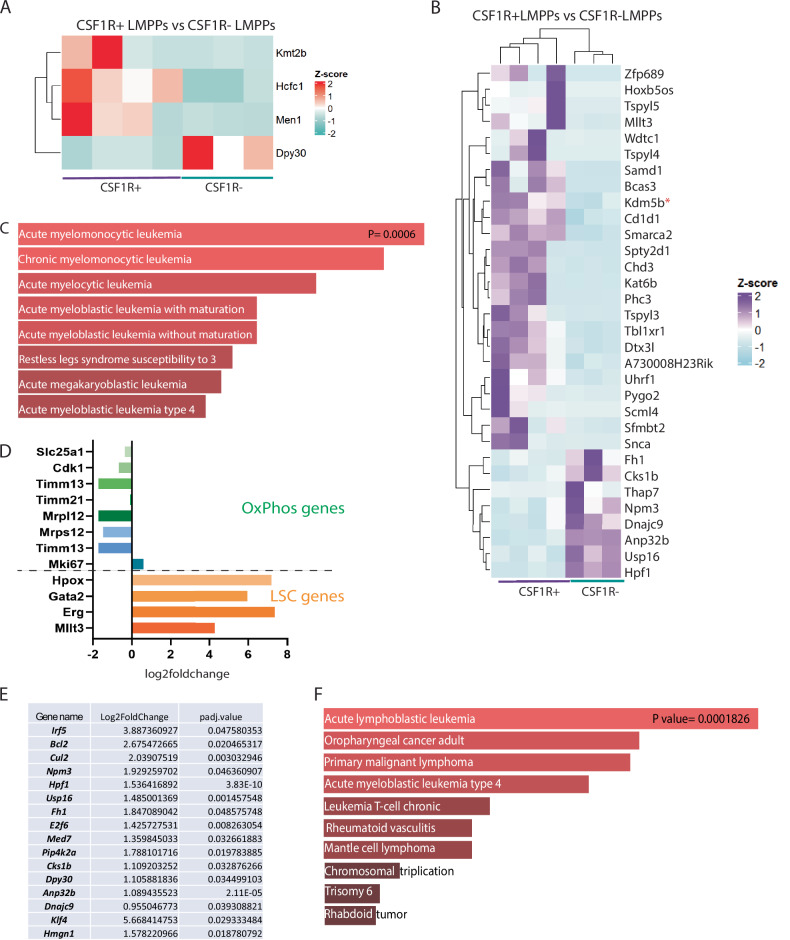
The KMT2A::MLLT3+ CSF1R+ LMPP transcriptome is AML-biased. **A** Heatmap showing a list of the most overrepresented KMT2A complex genes in KMT2A::MLLT3+ CSF1R+ versus KMT2A::MLLT3+ CSF1R- LMPPs. **B** Heatmap of the most overrepresented histone-binding genes in KMT2A::MLLT3+ CSF1R+ LMPPs versus KMT2A::MLLT3+ CSF1R- LMPPs. **C** Gene set enrichment analysis of the DEGs in KMT2A::MLLT3+ CSF1R+ LMPPs showing as most enriched term acute myelomonocytic leukaemia (*p* = 0.0006); data obtained from Rare Diseases GeneRIF Gene Lists. Gene lists for the heatmaps were obtained from DAVID, Gene Ontology terms were obtained from RStudio, and Gene Set Enrichment analysis from EnrichR. **D** Bar graph showing Log2 fold change of OxPhos-related genes (*Slc25a1, Cdk1, Timm13, Timm21, Mrpl12, Mrps12, Mki67*) and leukaemia stem cell (LSC) genes (*Hpox, Gata2, Erg, Mllt3*) in KMT2A::MLLT3+ CSF1R+ LMPPs. Genes were selected from Zhang et al. paediatric AML sc-RNA sequencing dataset [45]. **E** List of upregulated genes in KMT2A::MLLT3+ CSF1R- LMPPs related to KMT2A complex and histone-binding, and pathways in cancer annotations. Data are shown as gene name with log2FC and padj. values. **F** Gene set enrichment analysis of the upregulated genes in KMT2A::MLLT3+ CSF1R- LMPPs indicating acute lymphoblastic leukaemia as most enriched term (*p* = 0.0001). Data obtained from Rare Diseases GeneRIF Gene Lists, and Gene Set Enrichment analysis from EnrichR.

We next sought to identify in our dataset key regulators and co-factors cooperating with the KMT2A-r complex in KMT2A::MLLT3+ CSF1R+ LMPPs to induce and maintain AML. We found genes coding for members of the KMT2A complex and histone-binding genes to be upregulated (Fig. 5A,B). In particular, genes known to cooperate with the KMT2A complex in regulating Hox genes, such as *Men1* and *Hcfc1* [40, 41], were found to be upregulated in KMT2A::MLLT3+ CSF1R+ LMPPs (Fig. 5A). One of the hallmarks of KMT2A-r leukaemia are wide-spread transcriptional and epigenetic changes, including alterations to histone modifications. Among the most overrepresented histone-binding genes was *Kdm5b* (Fig. 5B, red asterisk), recently demonstrated to be a key factor in sustaining tumorigenicity of AML [42]. Moreover, we observed an enriched signature for interferon γ response (Fig. 4D lower panel). IFNγ signalling has emerged as a major player in promoting cancer and leukaemia progression [43], and recent evidence suggests a correlation between IFNγ signalling score and drug resistance in primary AML cells [44]. Indeed, analysis of our DEGs using EnrichR revealed enriched databases associated with AML with the highest scores (Fig. 5C). We then compared our dataset with a published single-cell RNA sequencing dataset of paediatric patients with AML [45]. We found that leukaemic and progenitor cells from AML patients and our KMT2A::MLLT3+ CSF1R+ LMPPs displayed a similar upregulation of key HSC-like/leukaemic stem cell (LSC) genes such *as Mllt3, Erg, Gata2*, and *Hopx* genes, and show downregulation of OXPHOS (*Slc25a1, Cdk1, Timm13, Timm21, Mrpl12, Mrps1, Mki67*) genes (Fig. 5D). In contrast, upregulated genes in KMT2A::MLLT3+ CSF1R- LMPPs revealed enrichment with high scores for datasets associated with acute lymphoblastic leukaemia (Fig. 5E,F). Overall, these results suggest that, within the murine foetal LMPP cell population, AML arises from KMT2A::MLLT3+ CSF1R+ LMPPs since many key drivers and mechanisms associated with both development and progression of AML were found to be upregulated in this cell subset.

### CSF1R and autophagy contribute to KMT2A::MLLT3+ CSF1R+ LMPP functionality

**Fig. 6:**
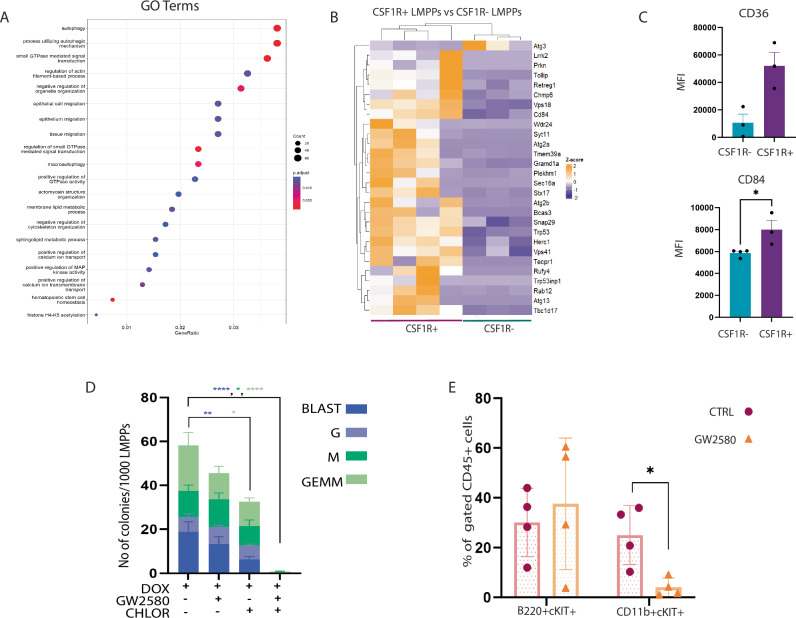
CSF1R and autophagy contribute to KMT2A::MLLT3+ CSF1R+ LMPP functionality. **A** Gene Ontology (GO) term enrichment analysis for biological processes of the DEGs in KMT2A::MLLT3+ CSF1R+ LMPPs. **B** Heatmap showing a list of the most overrepresented autophagy-related genes in KMT2A::MLLT3+ CSF1R+ versus KMT2A::MLLT3+ CSF1R- LMPPs. **C** CD36 and CD84 expression assessed by flow cytometry in KMT2A::MLLT3+ CSF1R-/+ LMPPs (*n* = 3). Data are presented as median fluorescence intensity (MFI), and statistical analysis was calculated with Unpaired T-test: *p* < 0.01 (*). **D** BLAST, CFU-M, CFU-G and CFU-GEMM colony output of KMT2A::MLLT3+ CSF1R+ LMPPs in CFU-myeloid assay (*N* = 3). Doxycycline (1 μg/ml), GW2580 (10 μM), and Chloroquine (10 μM) were added to the media as indicated. Data are presented as mean with SD, and statistical analysis was calculated with Dunnett’s multiple comparison test: *p* < 0.01 (*), *p* < 0.001 (**), *p* < 0.0001 (****). **E** Flow cytometry assessment of lymphoid (B220+ cKIT+ ) and myeloid (CD11b+ cKIT+ ) cell output of CSF1R+ LMPPs at 7 days after coculturing with and without 10 µM GW2580 (chemical inhibitor of CSF1R). 50 ng/ml IL7 was added to both experimental conditions (*N* = 4 biological replicates, 3 experiments/litters). Data are presented as mean with SD, and statistical analysis was calculated with Mann-Whitney U test: *p* = 0.0286 (*). CFU-M (colony-forming unit macrophages), CFU-G (colony-forming unit granulocytes), CFU-GEMM (colony-forming unit granulocyte/erythrocyte/monocyte/ megakaryocyte).

Within the E14.5 KMT2A::MLLT3+ CSF1R+ LMPP dataset, we observed enriched GO terms for autophagy and autophagy-related processes with associated key genes (Fig. 6A,B), which are known to be essential in AML for sustaining disease progression and resistance to therapy [46]. Using flow cytometry, we confirmed increased expression levels of CD36 and CD84 [47, 48] compared to their negative counterpart (Fig. 6C). The latter has been recently suggested as a therapeutic target for other cancer types [49]. Interestingly, earlier data from the Leukaemia MILE Study [50] indicated upregulation of *CD84*, together with other autophagy-related genes from our dataset (i.e. *SNAP29*, *SX17*) and other well-known autophagy-initiating genes (i.e. *ULK* genes), in KMT2A-r AML samples compared to other AML subtypes and healthy bone marrow (Supplementary Fig. S1A). *CD84* and *SNAP29* were also shown to be more highly expressed in KMT2A-r AML in a more recent dataset (Supplementary Fig. S1B). Therefore, we wanted to understand the impact of autophagy on CSF1R+ LMPP fitness and functionality. To this aim, we carried out colony-forming assays under myeloid conditions of freshly sorted CSF1R+ LMPPs in the presence of chloroquine, a well-known autophagy inhibitor. Our data demonstrated that chloroquine was able to significantly decrease both blast-like and GEMM colonies (Fig. 6D). These results confirm that autophagy plays a role in sustaining cellular function and viability of KMT2A::MLLT3+ CSF1R+ LMPPs in vitro.

Given that only KMT2A::MLLT3+ CSF1R+ LMPPs gave rise to AML in secondary transplants, we next wanted to determine whether CSF1R could be functionally involved in the choice between the lymphoid and the myeloid lineage in this subset. We cultured KMT2A::MLLT3+ CSF1R+ LMPPs on MS5 stromal cells in the presence of the CSF1R inhibitor, GW2580. In this culture system, factors promoting both lymphoid and myeloid differentiation were supplied to LMPPs, with IL7 added to the culture media, while myeloid factors, like CSF1, were released by MS5 cells in the culture system [20]. After 7 days of coculture, flow cytometry revealed that KMT2A::MLLT3+ CSF1R+ LMPPs produced both myeloid (CD11B+ cKIT+ ) and lymphoid (B220+ cKIT+ ) progenitor cells. Strikingly, GW2580 addition resulted in a loss of the myeloid phenotype (Fig. 6E), suggesting that CSF1R is responsible for the myeloid potential of LMPPs.

In light of these data, we then sought to determine whether CSF1R inhibition with GW2580, alone and in combination with chloroquine, could also affect the colony-forming capacity of CSF1R+ LMPPs. Importantly, CSF1R inhibition in combination with the inhibition of autophagy completely abrogated the clonogenic potential of KMT2A::MLLT3+ CSF1R+ LMPPs (Fig. 6D).

In the Leukaemia MILE study, CSF1R expression was found upregulated in KMT2A-r AML leukaemia in comparison with the other two most common infant AML subtypes (t(15;17) and t(8;21)) (Supplementary Fig. S1C), suggesting that CSF1R inhibition may represent a therapeutic opportunity for this patient group. To test this, we used the KMT2A::MLLT3+ AML cell lines THP-1, obtained from a 1-year-old patient, and NOMO-1 obtained from a 30-year-old patient, which both express CSF1R (Supplementary Fig. S1D). Treatment for 24 h of both cell lines with GW2580 resulted in increased cell death, which was strikingly higher in the paediatric THP-1 cells (*p = 0.0286) than in NOMO-1 cells (*p = 0.038) (Supplementary Fig. S1E). In contrast to the effect of chloroquine on primary foetal cells, established AML cell lines appear to be autophagy-independent.

### CSF1R+ LMPPs are present in human foetal development

**Fig. 7:**
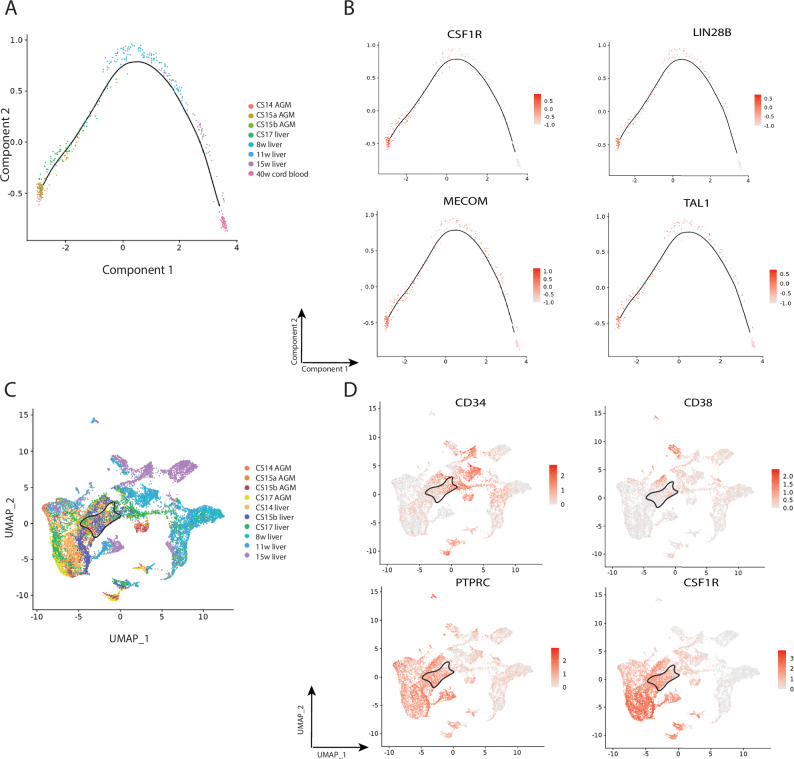
CSF1R+ LMPPs are present in human foetal development. **A** Pseudo-time analysis originated with Monocle of clustered AGM, liver and cord blood from CS14 to 40 weeks embryos/foetuses. **B** *CSF1R*, *LIN28B*, *MECOM* and *TAL1* pseudo-time expression. **C** Uniform Manifold Approximation and Projection (UMAP) analysis of haematopoietic clusters from AGM and liver from CS14 to 15 weeks embryos. **D** Feature plots showing the expression of LMPP marker genes (Lin- CD34+ CD38- CD45RA+) and *CSF1R* expression in the LMPP-containing area. *PTPRC*: gene encoding CD45RA protein marker. Black circles indicate LMPP-containing area.

To confirm that CSF1R+ LMPPs are also a feature of human foetal haematopoietic development, we analysed a publicly available dataset of human haematopoietic cells during embryonic life (The Atlas of Human Hematopoietic Stem Cell Development; [51]). To track *CSF1R* expression during haematopoietic stem cell ontogeny, we performed a pseudo-time analysis of the main haematopoietic tissues during foetal life - AGM, liver and cord blood (CB) from Carnegie stage (CS)14 up to 40 weeks of gestation (Fig. 7A). The highest gene expression levels of *CSF1R* coincided with the earliest stages of embryonic development (from CS14 to 8-week liver) on the temporal trajectory (Fig. 7B). Interestingly, the *CSF1R* expression pattern was similar to well-known HSC-like/self renewal-associated transcription factors, such as *MECOM* and *TAL1* and *LIN28B*, a foetal-restricted gene, the expression of which is maintained in infant and childhood leukaemia [14, 15, 52] (Fig. 7B). We then looked for the presence of cells with an LMPP phenotype (Lin- CD34+ CD38- CD45RA+ ; [52]) within this dataset (Fig. 7C) and found that clusters CS14 AGM to CS17 Liver (highlighted with solid black line) display the LMPP phenotype (Fig. 7D and Supplementary Fig. S2A,B). Importantly, *CSF1R* was also expressed in these cells (Fig. 7D), along with *LIN28B*, *HOPX*, *SOCS2*, and *MKI67* (Supplementary Fig. S2C), which were upregulated in our murine KMT2A::MLLT3+ CSF1R+ LMPPs, and in HSC-like leukaemic cells of paediatric AML patients in the Zhang et al. dataset [45]. These data suggest that CSF1R+ LMPPs also exist in human foetal development, and express key genes found to be upregulated in both our murine and paediatric patient AML datasets.

## Discussion

In this study, our aim was to understand whether the lineage choice between B-ALL and AML in KMT2A::MLLT3+ infant leukaemia could lie in a specific subset of foetal LMPPs which do or do not express CSF1R. To date, there is little knowledge of the mechanisms driving the choice between AML or B-ALL in infants with the t(9;11) translocation [11, 37]. LMPPs have been identified in murine [18] and human foetal livers [52] and demonstrated to retain both lymphoid and myeloid programmes. Within mouse foetal LMPPs, a subset was found to express CSF1R [17, 20, 53] and shown to have both B and myeloid potential. CSF1R has gained attention in recent years, since CSF1R-expressing macrophages in the AML microenvironment were suggested to support leukaemia cells. In addition, CSF1R is also found on AML cells, and exposure of primary AML patient samples to chemical inhibitors of CSF1R led to reduced cell viability of leukaemic cells [23]. Most importantly, CSF1R has recently been identified as a target for CAR-T cell therapy in adult AML, resulting in efficient eradication of leukaemic cells without any apparent toxicity [25]. Taken together, these recent observations prompted us to investigate the function of CSF1R+ LMPPs, and of CSF1R itself, in initiating KMT2A::MLLT3+ leukaemia. Our data show that KMT2A::MLLT3-expressing CSF1R+ LMPPs are characterised by high plasticity, able to grow under lymphoid and myeloid conditions and to produce transformed blast-like colonies with a mixed phenotype. This is in line with what was previously found in wild-type foetal liver, where expression of CSF1R in MPPs and Pro-B cells was associated with co-expression of lymphoid and myeloid genes. Interestingly, these bi-phenotypic populations were only found in the foetal liver and not in adult bone marrow [20, 53].

The difference in lineage output of these two LMPP populations was also apparent in vivo. Strikingly, for the first time, a B-cell biased donor reconstitution, enriched for pro-B cells, was observed in those recipients that received KMT2A::MLLT3+ CSF1R- murine LMPPs. In support of this, genes differentially expressed in the CSF1R- subset are associated with ALL. This suggests that lack of CSF1R expression on LMPPs might endow them with a lymphoid lineage bias. This lymphoid bias, however, never transformed into overt ALL. Instead, after a long latency, the mice eventually succumbed to AML. The reason for this sudden switch is unclear, but may reflect the outgrowth of a minor myeloid clone with a more aggressive phenotype. Heterochronic transplantations have shown that the age of the haematopoietic microenvironment contributes to age-specific leukaemia phenotypes, with the neonatal microenvironment being more permissive for a lymphoid-mixed phenotype leukaemia, and an aged microenvironment more likely to support myeloid disease [54, 55]. It may therefore be important to test the lineage potential of CSF1R- LMPPs in a younger microenvironment in the future.

In contrast, blood reconstitution from KMT2A::MLLT3+ CSF1R+ LMPPs was myeloid-biased already at 8 weeks post injection, and mice showed a shorter disease latency compared to CSF1R- recipients. Most importantly, only CSF1R+ LMPPs could propagate the disease in secondary recipients (with reduced latency), with extensive AML-like blast infiltration in peripheral blood, spleen, liver and central nervous system, indicating that KMT2A::MLLT3+ CSF1R+ LMPPs have LSC properties. In adult KMT2A::MLLT3+ AML, both HSCs and GMPs were shown to be able to initiate AML, albeit with different phenotypic outcomes [13, 56]. This points to fundamental differences between AML in different patient age groups, which may be a consequence of CSF1R+ LMPPs being a transient, foetal-restricted population.

The difference between the two LMPP sub-populations was reflected in their transcriptome, with KMT2A::MLLT3+ CSF1R+ LMPPs carrying a prominent AML and stem cell signature in support of their AML LSC properties. There was some heterogeneity amongst the KMT2A::MLLT3+ CSF1R+ replicates which was likely due to the relatively low number of sorted primary cells being available for this subpopulation. Notwithstanding, our findings suggest that autophagy is crucial for KMT2A::MLLT3+ CSF1R+ LMPPs functionality and ability to expand, with CSF1R being potentially involved in such mechanisms. Indeed, while autophagy and CSF1R inhibition alone partially reduced the ability of KMT2A::MLLT3+ CSF1R+ LMPPs to produce blast-like and GEMM colonies, inhibition of both showed a synergistic effect and completely abrogated any colony formation. Similarly, genetic deletion of the main genes involved in the autophagic flux in a KMT2A::MLLT1 AML mouse model resulted in delayed disease progression [57]. Further to previous work that reported autophagy is crucial for the development of KMT2A::MLLT3-driven adult AML [58], our data show that in foetal KMT2A::MLLT3+ CSF1R+ LMPPs such mechanisms could be in part regulated by CSF1R. Interestingly, in the adult model, autophagy seems important only for disease initiation. Indeed, autophagy inhibition in established AML cell lines did not affect cell viability. While it is extensively proven that the biology of paediatric and adult leukaemia is quite different from infant leukaemia, and that the latter share similarities with foetal-restricted haematopoietic stem cells [59] the role of autophagy in a faithful model of infant KMT2A::MLLT3+ AML could be further elucidated in the future. Interestingly, treatment with the CSF1R inhibitor of THP-1 cells resulted in significantly higher cell death. In this context, CSF1R may not only serve as a marker of LSC potential, but appears to be functionally involved in sustaining myeloid disease, as has been suggested for adult AML. Gottschlich et al. [25] have recently generated CSF1R-targeting CAR-T cells with promising results for the treatment of adult AML. These CSF1R-targeting CAR-T cells may therefore also be of benefit to infant patients.

## Supplementary information

Supplementary Figures

Supplementary Table 1

Supplementary Table 2

Supplementary Table 3
