# Supplementary material for: CSF1R marks a subset of foetal haematopoietic multipotent progenitor cells with acute myeloid leukaemia propagation properties
Source: Leukemia. 2026 Jan 16;40(3):540–52. doi: 10.1038/s41375-025-02856-4 (PMC12960200; doi:10.1038/s41375-025-02856-4)
Supplement: Supplementary file 1 — Supplementary Figures [file 41375_2025_2856_MOESM1_ESM.pdf]

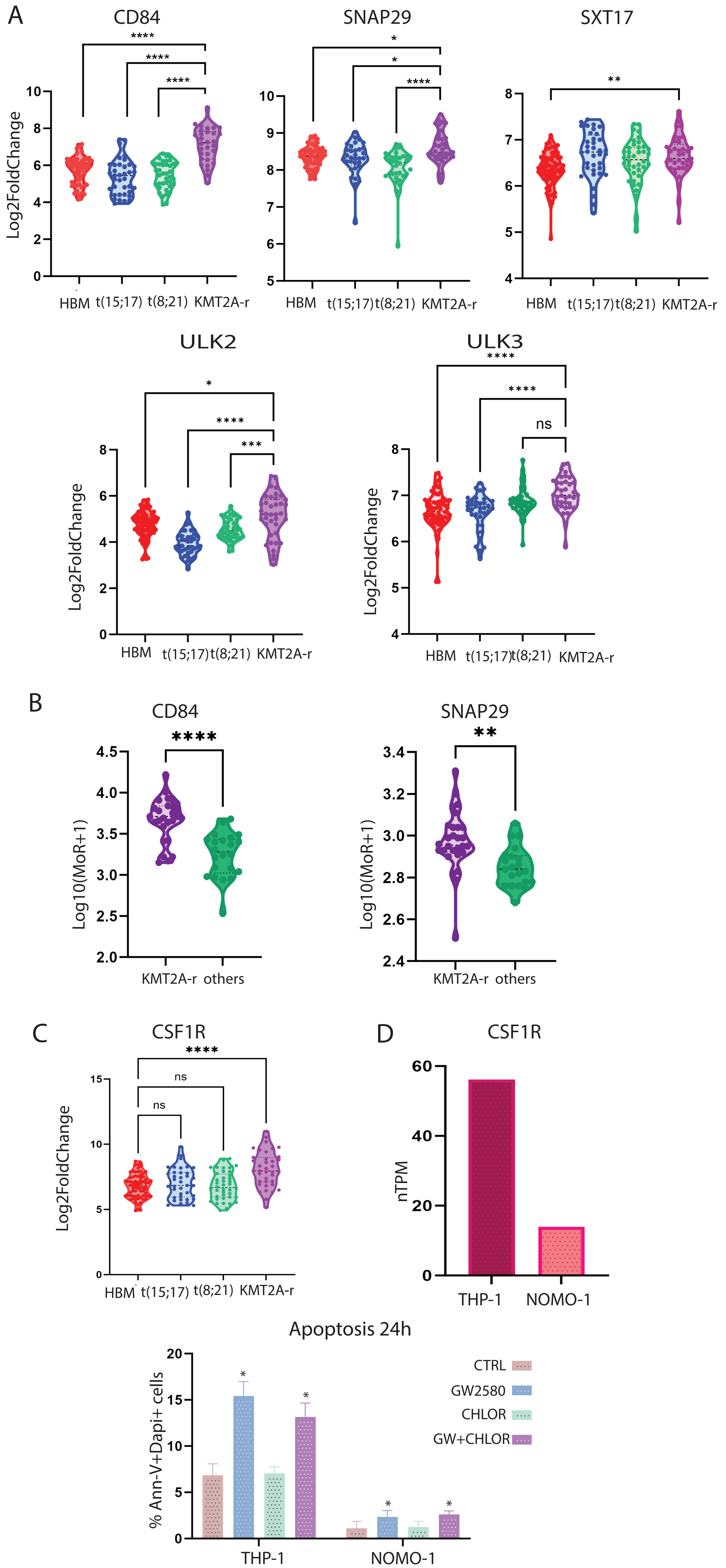

Supplementary figure 1

Supplementary Figure 1

(A) Violin plots showing gene expression levels of autophagy-related genes (*CD84*, *SNAP29*, *SXT17*, *ULK2*, *ULK3*) in several AML subtypes (t(15;17); t(8;21); KMT2A-r) and healthy bone marrow (HBM). (B) Violin plots showing gene expression of *CD84* and *SNAP29* in several KMT2A-r AML and other AML subtypes (t(15;17); t(8;21)). Data (indicated as Log10(MoR+1) were published on St Jude Cloud (doi: 10.1158/2159-8290.CD-20-1230); MoR=Median of Ratio. Statistical analysis was calculated with t-test: $p<0.001$ (**), $p<0.0001$ (****). (C) Violin plot indicating *CSF1R* gene expression levels in several AML subtypes (t(15;17); t(8;21); KMT2A-r) and healthy bone marrow (HBM). Data (indicated as Log2 fold change) were published in the LEUKEMIA MILE Study and were obtained from BloodSpot (doi: 10.1093/bioinformatics/btz931). (D) Bar graph indicating transcript levels of CSF1R in THP-1 and NOMO-1 cells, expressed as transcripts per million (nTPM). Data were obtained from the Human Protein Atlas proteinatlas.org. (E) Bar graph indicating percentages of apoptotic cells (expressed as %AnnexinV+Dapi+ cells) in THP-1 and NOMO-1 cell lines treated for 24 hours with 10μM GW2580. Statistical analysis was calculated with Mann Whitney U test: $p=0.0286$ (*).

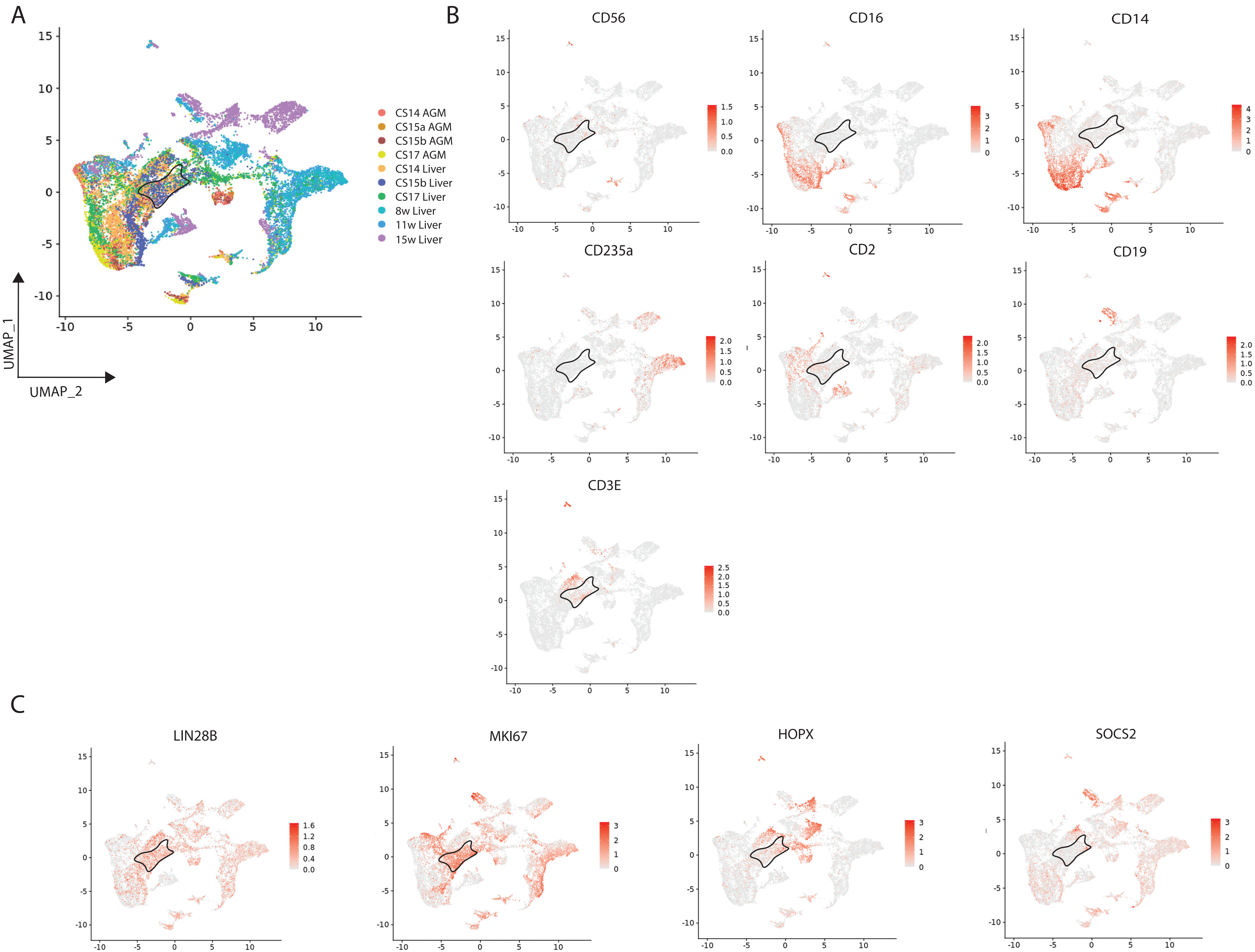

Supplementary figure 2

Supplementary Figure 2

(A) UMAP visualization of CS14 AGM, CS15a AGM, CS15b AGM, CS17 AGM, CS14 Liver, CS15b Liver, CS17 Liver, 8-11-15w Liver clusters (AGM: aorta gonad mesonephros; CS: Carnegie Stage) taken from “The Atlas of Human Hematopoietic Stem Cell Development” (doi: 10.3390/biology10060552). (B) Gene expression levels of marker genes associated with mature blood cell lineage (*CD2*, *CD3E*, *CD19*, *CD14*, *CD16*, *CD56*, *CD235a*. *NCAM1*: gene coding for CD56, *GYPA*: gene coding for CD235a, *FCGR3A*: gene coding for CD16), visualized on UMAP. (C) Gene expression levels of leukaemic stem cell- (*LIN28B, HPOX*) and OxPhos-related (*SOCS2, MKI67*) genes, as shown above in Zhang et al. dataset (doi: 10.1186/s13059-023-03031-7). Black circles indicate LMPPs-containing area.
