# Supplementary material for: CSF1R marks a subset of foetal haematopoietic multipotent progenitor cells with acute myeloid leukaemia propagation properties
Source: Leukemia. 2026 Jan 16;40(3):540–52. doi: 10.1038/s41375-025-02856-4 (PMC12960200; doi:10.1038/s41375-025-02856-4)
Supplement: Supplementary file 2 — Supplementary Table 1 [file 41375_2025_2856_MOESM2_ESM.pdf]

genotyping conditions

Temperature	Time	Number of cycles
94 C	2 min	-
94 C	20 sec	10
65 C (goes down 0.5 C every cycle)	15 sec	
68 C	10 sec	
94 C	15 sec	20
60 C	15 sec	
72 C	10 sec	
72 C	3 min	-
HOLD		
