# Supplementary material for: CSF1R marks a subset of foetal haematopoietic multipotent progenitor cells with acute myeloid leukaemia propagation properties
Source: Leukemia. 2026 Jan 16;40(3):540–52. doi: 10.1038/s41375-025-02856-4 (PMC12960200; doi:10.1038/s41375-025-02856-4)
Supplement: Supplementary file 3 — Supplementary Table 2 [file 41375_2025_2856_MOESM3_ESM.pdf]

genes significantly downregulated in CSF1R+ LMPPs vs CSF1R-LMPPs

gene symbol	baseMean	log2FoldChange	lfcSE	stat	pvalue
9430069I07Rik	664.4173421	-13.50072396	1.587295552	-8.505488435	1.81E-17
Il13ra1	549.9087078	-13.22779627	2.464988949	-5.366270009	8.04E-08
Adgre4	473.0086852	-13.01035571	1.494641246	-8.704667921	3.19E-18
Gm32196	362.2625455	-12.6255551	3.224394145	-3.915636405	9.02E-05
Cd300c	292.6318995	-12.31764469	3.187270906	-3.86463688	0.000111255
Gm35154	204.7407758	-11.8022755	3.271655101	-3.607432671	0.000309242
Gm49625	186.2204717	-11.66546112	3.277322383	-3.559448769	0.000371634
Gm38843	182.0313166	-11.63264121	3.307388091	-3.517168499	0.000436177
Nrg1	169.3690198	-11.52866193	3.479369189	-3.313434506	0.000921577
Mblac1	154.8618989	-11.39930294	2.404245488	-4.741322381	2.12E-06
Cd300ld	681.5223957	-10.64063035	2.015525291	-5.279333581	1.30E-07
Or10aa3	81.01345651	-10.4646973	3.318966912	-3.152998382	0.001616027
Gm26770	77.48474792	-10.40067311	3.285129446	-3.165985778	0.001545584
Cftr	52.47614781	-9.838137262	3.63166257	-2.708989911	0.006748839
Smkr-ps	149.2284765	-9.833450427	2.036301776	-4.829073245	1.37E-06
Pmaip1	2397.469558	-9.390578498	1.163564553	-8.070526449	7.00E-16
Plbd1	89.80291191	-9.093202655	2.19109937	-4.150064018	3.32E-05
Ifitm6	4947.441159	-9.04768514	2.582044876	-3.504077418	0.000458192
Clec7a	2087.815601	-8.913035445	2.390433082	-3.728627885	0.000192525
Ms4a4c	4810.602075	-8.435074712	2.648642787	-3.184678112	0.001449151
Cpb2	56.81072965	-8.413636861	2.947943803	-2.854069624	0.004316307
Mrgpra2a	166.3203593	-8.195539236	2.175328256	-3.767495418	0.000164894
Adgra2	40.38492828	-8.051790388	2.022519379	-3.981069587	6.86E-05
Thbs1	231.4625061	-7.759115317	2.107514708	-3.68164231	0.000231736
Ms4a4b	1106.881695	-7.390903919	2.694162252	-2.743303196	0.00608245
9130604C24Rik	372.1321946	-7.270981108	2.125362854	-3.421054007	0.000623789
Plcxd2	21.74695063	-7.14232561	1.671668584	-4.272572733	1.93E-05
Hdac9	880.6628828	-7.082687968	1.508182785	-4.69617346	2.65E-06
Pilra	521.7185158	-6.937274823	1.869297707	-3.71116639	0.000206306
Plcd4	28.58085714	-6.808413802	2.532465278	-2.688452971	0.007178395
Ms4a4d	13.18618554	-6.418825967	2.228672147	-2.880112256	0.003975336
Cx3cr1	387.6168119	-6.281046471	2.291261333	-2.741305141	0.006119565
Ctsh	1359.466769	-6.146410638	1.636207043	-3.756499316	0.000172307
Thap7	257.7837417	-6.142283677	1.586521186	-3.871542171	0.000108149
Gpr183	2622.511434	-6.095795166	2.170962565	-2.807876683	0.004986932
Ccl6	4170.428839	-5.882985883	1.614583913	-3.643654465	0.000268794
Mrgpra2b	207.2037862	-5.849618965	1.928173122	-3.033762321	0.002415246
Rassf4	5041.554284	-5.83353412	1.26532909	-4.610290055	4.02E-06
Ifi211	1329.716611	-5.711852892	1.336298599	-4.274383658	1.92E-05
Smad6	56.17406465	-5.707392038	2.133988214	-2.674519006	0.007483653
Nupr1	1718.664267	-5.672935447	1.536145221	-3.692968198	0.000221652
Klf4	329.6531588	-5.668414753	1.960220788	-2.891722599	0.003831361
Ms4a6c	30621.2904	-5.351626357	0.803197511	-6.662902065	2.68E-11
Nfkbil1	52.03117397	-5.123347058	1.737932601	-2.947954975	0.003198837
Id1	114.3123879	-4.963966273	1.678717491	-2.956999197	0.003106489
Arhgef12	161.5032951	-4.933008563	1.13710786	-4.338206371	1.44E-05
BE692007	2321.320382	-4.658805759	1.602619666	-2.906994003	0.003649202
Bcl2a1d	699.5356196	-4.582970504	1.638116082	-2.797708023	0.005146661

Cadm1	23.64678098	-4.502697517	1.676677996	-2.685487331	0.007242412
Ctsz	3746.937422	-4.364358265	0.875943235	-4.982467001	6.28E-07
Ccr5	686.3805104	-4.275564777	1.354239785	-3.157169672	0.001593086
Ly86	5439.581435	-4.262188081	0.696077545	-6.123151234	9.17E-10
Nrp1	776.6060454	-4.236941856	1.373451426	-3.084886568	0.002036298
2310039H08Ril	189.9062745	-4.23046002	1.275069912	-3.317825933	0.00090721
Pno1	5499.67398	-4.164620843	0.959890689	-4.338640733	1.43E-05
Prtn3	5262.963469	-3.966246162	1.070967468	-3.703423567	0.000212709
Irf5	159.5339105	-3.887360927	1.447796563	-2.685018756	0.007252574
Sdf2l1	336.3873646	-3.772372559	1.256575152	-3.002106601	0.002681183
Bin1	181.0237602	-3.701596229	1.284381743	-2.882006263	0.003951519
Ccr2	13107.80883	-3.6870778	0.79112682	-4.660539508	3.15E-06
Cybb	5807.23872	-3.630298615	0.700160172	-5.184954472	2.16E-07
Rhob	306.3778773	-3.572206446	0.981053565	-3.641194095	0.000271376
Gm5552	123.0987715	-3.265839808	0.846806034	-3.856656279	0.000114949
Casp1	9890.866805	-3.251001036	0.636281873	-5.109372392	3.23E-07
Atg3	1356.919497	-3.247077783	1.056881475	-3.072319707	0.002124021
Anxa3	9839.053332	-3.229369352	0.568137745	-5.684130975	1.31E-08
Spcs2	10946.21545	-3.176052882	1.001406241	-3.171592858	0.001516054
Atp8b4	10386.492	-3.130874714	0.7272559	-4.305052338	1.67E-05
Clec12a	11294.27721	-3.010285542	0.685058865	-4.394199823	1.11E-05
Gtf2f2	2600.194121	-2.938508555	0.643536572	-4.566187351	4.97E-06
Nptn	430.6438761	-2.884173422	1.069671314	-2.696317444	0.007011082
Mrm3	1106.398679	-2.827876395	0.811570779	-3.484448269	0.000493153
Gm46610	93.58543092	-2.769984852	0.90747668	-3.052403345	0.002270168
Jagn1	2123.304947	-2.752727254	0.768655953	-3.581221538	0.000341992
NA	17.57482875	-2.727695737	0.908178981	-3.003478163	0.002669127
Bphl	2017.730973	-2.692244837	0.817656277	-3.292636421	0.000992527
Bcl2	946.9241803	-2.675472665	0.882176406	-3.032809139	0.002422888
Selenos	11933.81846	-2.647611914	0.991068014	-2.671473477	0.007551904
Gapt	9087.637363	-2.641677269	0.512450939	-5.154985715	2.54E-07
Rufy1	949.6753908	-2.619729641	0.927107985	-2.825700655	0.004717733
Cdc34	2033.656131	-2.609889501	0.729577879	-3.57725964	0.000347215
Igsf6	11813.7993	-2.600606329	0.622987876	-4.174409213	2.99E-05
Id2	10221.44541	-2.5643814	0.697870068	-3.674582876	0.000238238
Ms4a6b	11202.39435	-2.526255156	0.692940422	-3.64570326	0.000266662
Cdca8	5139.558235	-2.510666957	0.774190055	-3.242959452	0.00118295
Dusp22	2768.870554	-2.495243466	0.765660428	-3.258942704	0.001118283
Cd74	8966.675534	-2.492587593	0.682600353	-3.651606072	0.000260605
Ctsc	17363.04869	-2.447203433	0.587009312	-4.168934601	3.06E-05
Gm9392	245.6891455	-2.43692986	0.590251904	-4.128626852	3.65E-05
Gm3375	244.9949987	-2.436622195	0.728256376	-3.345830225	0.000820366
Gm6540	139.6601168	-2.432871859	0.698602818	-3.482482172	0.000496788
Gm37696	99.91729682	-2.408275064	0.84696314	-2.843423697	0.004463169
Sucla2	4305.182115	-2.393922748	0.797716414	-3.000969652	0.002691214
Psmd6	5725.186031	-2.38856437	0.874038092	-2.732792075	0.006279996
Hspa5	60678.33993	-2.378501927	0.868294717	-2.73927951	0.0061574
Alkbh7	263.5659209	-2.284030386	0.852802598	-2.678263868	0.007400489
Gm7979	29.0973566	-2.258917816	0.769599944	-2.935184487	0.003333494
Sat1	39493.02854	-2.25802288	0.677569326	-3.332534093	0.000860589

Apoc1	782.3563905	-2.22678336	0.798275293	-2.789493022	0.005279063
Gm15361	137.2843381	-2.216787508	0.525326294	-4.219829721	2.44E-05
Rab32	2721.617427	-2.214724227	0.569497698	-3.888908131	0.000100696
Arl6ip4	3687.793813	-2.191019938	0.570423142	-3.841043214	0.000122513
Gm32340	18.51473021	-2.190316386	0.703591295	-3.11305214	0.001851633
Plac8	101421.5435	-2.168425658	0.694128082	-3.123956102	0.001784371
Glipr1	18178.99284	-2.133632726	0.462446051	-4.613798131	3.95E-06
Psmb5	3002.194577	-2.127288939	0.695873163	-3.057006726	0.002235593
Erp29	5304.314953	-2.115238799	0.437472479	-4.835135695	1.33E-06
Fcer1g	11232.45232	-2.11407675	0.621702555	-3.400463343	0.000672718
Gm7464	19.04124998	-2.106471501	0.777739464	-2.708453922	0.00675975
Gm3571	1229.042042	-2.104610688	0.523158758	-4.022891057	5.75E-05
Hpgds	4581.065087	-2.081442212	0.652714444	-3.188901718	0.001428144
Psmg4	895.0079011	-2.073345626	0.680020576	-3.048945426	0.002296462
Gria3	6739.029391	-2.055701527	0.534737748	-3.844317211	0.000120889
Gm19880	131.5754824	-2.046377252	0.361039904	-5.668008517	1.44E-08
Ncf4	8996.178253	-2.042188545	0.642597402	-3.178021791	0.001482836
Cul2	5131.05207	-2.03907519	0.551045066	-3.700378274	0.000215278
Cox5b	1080.849549	-2.017610983	0.576560866	-3.499389401	0.000466325
Emb	15742.73611	-2.014222523	0.573988499	-3.509168787	0.00044951
Gm11868	61.05052414	-2.01420937	0.625465995	-3.220333939	0.001280414
Cox6a2	3041.844111	-2.004309666	0.610350503	-3.283866657	0.001023934
Gm8724	22.77642695	-1.998780979	0.595926	-3.354075808	0.000796305
Gm15946	460.6783364	-1.978100532	0.489773697	-4.038805152	5.37E-05
Mterf1a	1429.640668	-1.975477177	0.497895214	-3.967656485	7.26E-05
Rps12-ps10	102.6926284	-1.973572295	0.514747496	-3.834059053	0.000126046
Gm21181	20.65453173	-1.940856028	0.697795	-2.78141292	0.005412285
Gm14238	208.8472399	-1.935702326	0.365945835	-5.289586988	1.23E-07
Npm3	2201.26496	-1.929259702	0.714993016	-2.698291674	0.006969635
Rps12-ps4	157.2509946	-1.922992552	0.651884966	-2.949895539	0.003178814
Tpd52	8237.532469	-1.915008397	0.403866153	-4.741690743	2.12E-06
Cd52	13659.70097	-1.908683253	0.554338425	-3.443173283	0.000574931
Gm6440	425.1393605	-1.903237899	0.596081855	-3.192913666	0.00140845
Srsf9	1561.957904	-1.902749072	0.631635325	-3.012417129	0.002591762
Gm8702	255.8648726	-1.870489639	0.4499385	-4.157211793	3.22E-05
Gm34783	145.1012278	-1.83245895	0.453687642	-4.039032096	5.37E-05
Mrps7	2438.200707	-1.82889718	0.583737469	-3.133081698	0.001729813
Mecr	2140.960563	-1.824072574	0.590602983	-3.088491974	0.002011751
Mrps18c	21654.80558	-1.807112286	0.528010252	-3.422494694	0.000620493
Coq7	5561.023884	-1.801963286	0.660535094	-2.72803565	0.006371272
Gm13772	1553.893862	-1.800915057	0.201744151	-8.926727486	4.39E-19
Sec11c	11413.76802	-1.800580642	0.641282847	-2.807779206	0.004988442
Gm14237	54.81980305	-1.795220329	0.533163117	-3.367112752	0.000759596
2410006H16Rik	6432.307383	-1.787601762	0.650888737	-2.746401436	0.006025299
Hspe1-ps6	78.05634045	-1.779684674	0.555603771	-3.203154417	0.001359311
Gm13352	204.9947061	-1.765101446	0.560571924	-3.148751071	0.001639698
Naxe	738.6044158	-1.75828938	0.643359001	-2.732983263	0.006276352
Rpl36a-ps3	4693.719967	-1.751713669	0.5089146	-3.442058191	0.000577306
Gm45640	448.5518438	-1.751706451	0.625209672	-2.801790391	0.005081988
Gm15267	733.6685452	-1.747360363	0.621192074	-2.812914774	0.004909466

Gm20091	513.1426	-1.744342145	0.414747864	-4.205789338	2.60E-05
Gm7180	414.1746071	-1.734789758	0.344076158	-5.041877269	4.61E-07
Gm6419	1024.549948	-1.727146076	0.527094448	-3.27672978	0.001050168
Rpl23a-ps3	3941.857909	-1.722354478	0.564190174	-3.052790633	0.002267241
Mrpl12	2199.896346	-1.715768429	0.617816817	-2.7771475	0.005483829
Gm10053	3469.633368	-1.713503842	0.346858082	-4.940071831	7.81E-07
Strap	4498.588903	-1.709792144	0.614831438	-2.780912032	0.005420642
Gm47333	50.87993152	-1.703076917	0.40726353	-4.181756514	2.89E-05
Mrpl43	565.9336652	-1.696419869	0.565692868	-2.998835527	0.002710136
Gm32460	70.45947115	-1.692821692	0.63155465	-2.68040413	0.007353332
Cdkn3	7930.023171	-1.690955812	0.541083698	-3.125127993	0.001777277
Gm13252	236.7567168	-1.684510506	0.397221582	-4.24073258	2.23E-05
Gm12344	69.15594364	-1.683045157	0.465923371	-3.61227889	0.000303518
Gm13488	608.328588	-1.676403161	0.508468761	-3.296963925	0.000977361
Gm13182	853.2012435	-1.667979087	0.468111357	-3.563210042	0.000366347
Skil	4986.432752	-1.660347942	0.584578607	-2.840247526	0.004507854
Gm6421	313.6283865	-1.657221186	0.57231501	-2.895645154	0.0037838
Tmem126a	9925.437697	-1.655966055	0.573419679	-2.887877966	0.003878503
Bex6	12724.37558	-1.640000982	0.509118919	-3.221253267	0.001276313
Gm11625	394.4504394	-1.638760007	0.332246078	-4.932368245	8.12E-07
Cox7c-ps1	1238.8882	-1.637770733	0.446717169	-3.666236372	0.000246146
Rpl26-ps2	39.36608946	-1.633669086	0.509154241	-3.208593694	0.001333858
Pak1ip1	6170.728306	-1.617122064	0.460382015	-3.512565673	0.000443802
Phf11b	1645.333133	-1.612794408	0.59374773	-2.716295706	0.006601692
Cacybp	11949.77186	-1.605812765	0.370801807	-4.330649783	1.49E-05
Atp5pb-ps	535.3238093	-1.599955414	0.581506842	-2.751395682	0.005934192
Gm19807	104.7899081	-1.599494946	0.488830096	-3.272087705	0.001067565
Psmd12	31222.94392	-1.592602138	0.423957644	-3.756512382	0.000172298
Gm30431	2166.296008	-1.580314543	0.52433524	-3.013939217	0.002578795
Hmgn1	5088.352049	-1.578220966	0.515003842	-3.064483871	0.002180459
Cycs-ps2	605.6070447	-1.574940676	0.350909034	-4.488173644	7.18E-06
Naa38	7486.128395	-1.574244512	0.583835829	-2.696382157	0.00700972
Gm8539	283.9277095	-1.562959485	0.471105256	-3.31764391	0.000907801
Mrpl42	141658.8763	-1.553824944	0.500799499	-3.102688695	0.001917712
I830077J02Rik	4193.60344	-1.55234639	0.526280939	-2.949653453	0.003181305
Ndufs5-ps	2578.268758	-1.548325205	0.404259257	-3.830030301	0.000128127
Hpf1	2599.316506	-1.536416892	0.221249751	-6.94426494	3.80E-12
Gm2534	190.5847578	-1.521474949	0.479597754	-3.172397987	0.001511857
Dbnl	1601.764148	-1.514562733	0.389181234	-3.891664348	9.96E-05
BC035044	41589.14844	-1.509616797	0.451553032	-3.343166116	0.000828283
Pomp	119987.8548	-1.502080782	0.492265582	-3.051362589	0.002278053
Ywhah	5068.402515	-1.50098398	0.540797036	-2.775503342	0.005511633
Usp16	3851.405725	-1.485001369	0.378526121	-3.923114649	8.74E-05
Cycs	87372.98636	-1.483205745	0.395067055	-3.754313923	0.000173817
Gm14277	282.4876567	-1.479154955	0.428110513	-3.455077393	0.000550135
Gm50083	49.56006716	-1.475123891	0.527705312	-2.795355394	0.005184269
Gm14706	204.2973171	-1.471873961	0.32497078	-4.529250173	5.92E-06
Prdx5	5248.959932	-1.465927607	0.525921719	-2.787349436	0.005314114
Ostf1	2459.159311	-1.463062096	0.486416659	-3.007837145	0.002631141
Gm5481	212.0497668	-1.448915591	0.486880847	-2.975914126	0.002921166

Parp8	4192.912438	-1.44855381	0.539884027	-2.683083287	0.007294682
Fh1	2779.900737	-1.447089042	0.540880961	-2.6754298	0.00746335
Fdps	11945.8224	-1.442712293	0.4465489	-3.230804722	0.001234422
Rnf113a2	2412.473823	-1.434453917	0.401799399	-3.570074819	0.000356879
Tnni2	6318.624206	-1.432869982	0.521468741	-2.747758149	0.006000425
E2f6	1617.484062	-1.425727531	0.423727964	-3.364723722	0.000766203
NA	1086.575277	-1.423722424	0.496232433	-2.869063629	0.00411689
Atp5mc3	18602.47796	-1.421029359	0.472431716	-3.007904234	0.00263056
Mndal	18981.33802	-1.420453524	0.466465739	-3.045140094	0.002325719
Sap130	1137.736043	-1.419375495	0.448537595	-3.164451568	0.001553755
Bola2	13049.29361	-1.413077277	0.509489587	-2.773515521	0.005545419
Psmc3ip	13785.70168	-1.411896089	0.48487386	-2.911883287	0.003592568
Sumo3	4846.762359	-1.407561797	0.44957248	-3.130889586	0.001742777
Gm7676	238.5139822	-1.404742581	0.450615126	-3.117388879	0.001824607
Gm35106	158.7048567	-1.402920323	0.441496045	-3.17765094	0.001484734
Gm13602	77.94880797	-1.379088875	0.496283111	-2.77883499	0.005455423
Zbtb8os	8858.055601	-1.37058541	0.259339618	-5.284905648	1.26E-07
Gm11930	248.109531	-1.361359227	0.39221944	-3.47091217	0.000518694
Med7	5561.851168	-1.359845033	0.477594654	-2.847278589	0.004409476
Pycard	13187.02881	-1.344380897	0.496399319	-2.708264987	0.0067636
Gm45113	65.06661983	-1.3434104	0.404316576	-3.322669611	0.000891604
Golga5	1430.91595	-1.329353564	0.48777849	-2.725322236	0.006423876
Lyset	2742.508045	-1.328025676	0.460793302	-2.882042059	0.00395107
Hspe1-ps3	125.0843591	-1.324448392	0.455049819	-2.910556905	0.003607853
Gm5449	269.4991588	-1.322662513	0.371846753	-3.557009718	0.0003751
Rap1a	3857.070686	-1.321715869	0.337898853	-3.911572521	9.17E-05
Fau	42407.69716	-1.319039742	0.486156991	-2.713197105	0.006663746
Pcif1	628.3443774	-1.315525584	0.372014	-3.536226012	0.000405887
Gm10136	3268.64148	-1.315501496	0.457298359	-2.876680989	0.004018817
Gm6474	656.651029	-1.314075881	0.44088212	-2.980560614	0.002877213
Rbm8a2	556.6378155	-1.309075022	0.473175796	-2.76657224	0.005664903
Gm11945	265.0379348	-1.299908816	0.413683365	-3.14227964	0.001676378
Gm20900	554.5707364	-1.290709485	0.253028302	-5.101047886	3.38E-07
Gm9892	1679.036204	-1.284423487	0.405824177	-3.164975275	0.001550961
Gm5436	4295.391534	-1.275727681	0.47629075	-2.678464115	0.007396066
Lamtor5	33782.70173	-1.268217204	0.475736076	-2.665799945	0.007680538
Calr	12623.85067	-1.260930216	0.350410874	-3.598433471	0.00032014
Rps11-ps4	250.0533259	-1.260629423	0.390834917	-3.225477988	0.001257625
Mien1	3207.919398	-1.249185033	0.445198452	-2.805906057	0.005017532
Gm14925	125.5427081	-1.241706518	0.447865688	-2.772497542	0.005562794
Bbip1	18032.21382	-1.235105098	0.459239633	-2.689456677	0.007156844
Gm12734	264.0794266	-1.231692824	0.359296751	-3.428065577	0.000607899
Brcc3	33937.23528	-1.231588221	0.352232839	-3.496517317	0.000471374
Ndufs5	7417.814664	-1.230861342	0.420032464	-2.930395737	0.003385306
Brix1	104038.2898	-1.229668176	0.40898957	-3.006600326	0.002641869
Gm16477	198.337995	-1.229185579	0.384293109	-3.198562635	0.001381145
Rplp2	8531.770193	-1.226768066	0.298441081	-4.110587122	3.95E-05
Gm47894	129.30646	-1.22595501	0.418284433	-2.930912347	0.003379681
Smim29	808.6816709	-1.220842936	0.328949307	-3.711340649	0.000206164
Rbx1-ps	1547.616998	-1.215669071	0.381186238	-3.189173553	0.001426802

Gm29770	1825.309837	-1.21170493	0.297966922	-4.066575319	4.77E-05
Gm11826	387.2525628	-1.211124064	0.376075477	-3.22042818	0.001279993
Gm37361	152.6622708	-1.209197108	0.384957133	-3.141121446	0.001683022
Trappc2l	9038.736365	-1.208853411	0.451461712	-2.677643262	0.007414214
Exosc8	67962.33209	-1.203493788	0.328000149	-3.669186714	0.000243323
Ndufaf1	6199.077354	-1.203047346	0.271606976	-4.429368364	9.45E-06
Rpl23a	14836.44309	-1.198630584	0.445154957	-2.692614258	0.007089424
Gm7868	527.4670144	-1.198460809	0.280927524	-4.266085404	1.99E-05
Gm6563	11167.61657	-1.195927587	0.417714862	-2.863023792	0.004196191
Timm17a	61639.5159	-1.190502719	0.247419515	-4.811676707	1.50E-06
Pip4k2a	2997.800128	-1.188101716	0.390037892	-3.046118697	0.002318163
Vps26a	14055.29342	-1.186153434	0.338572917	-3.503391363	0.000459374
Pbdc1	36873.38407	-1.155658762	0.365129868	-3.165062248	0.001550498
Gm14017	233.8491281	-1.148294839	0.400208845	-2.869239035	0.004114607
Pnkd	3763.003328	-1.144827827	0.33110045	-3.457645031	0.000544919
Gm47681	322.9710532	-1.138271507	0.342482399	-3.323591262	0.000888663
Polr1c	20784.47269	-1.134227888	0.388707811	-2.917944681	0.003523469
Surf1	3268.650045	-1.133905018	0.321518289	-3.526720117	0.000420741
Gm8069	198.2522007	-1.131814955	0.308650927	-3.6669741	0.000245438
Rps11-ps1	1877.007588	-1.112989386	0.28605103	-3.890877045	9.99E-05
Cd53	33342.28589	-1.110136923	0.322216771	-3.44531081	0.000570403
Cks1b	84609.96637	-1.109203252	0.389938607	-2.844558687	0.004447299
Ppp1cc	1291.552814	-1.107023377	0.337015133	-3.284788334	0.00102059
Dpy30	48406.07887	-1.105881836	0.39136884	-2.82567676	0.004718085
Kpna2rt	1959.184554	-1.100472734	0.380129419	-2.894994914	0.003791647
Polr2i	4570.448895	-1.0927956	0.374363152	-2.91907896	0.003510673
Anp32b	9344.086616	-1.089435523	0.21871419	-4.98109209	6.32E-07
Ybx3	5490.318219	-1.080379855	0.391017197	-2.762998313	0.005727306
Gm3150	154.1263485	-1.072663145	0.388539766	-2.760755111	0.00576679
Rpl19-ps12	294.5716829	-1.068470065	0.313257935	-3.410831606	0.000647651
Gm13831	423.6358928	-1.064700604	0.342700542	-3.106795794	0.00189127
Ndufb10	33768.84159	-1.062968045	0.382759269	-2.777119018	0.005484309
Rps11-ps2	2011.643327	-1.051940736	0.362367524	-2.902966369	0.003696463
Rpl19-ps6	580.9824376	-1.04670018	0.368692017	-2.838955368	0.004526149
Mrps16	13477.10329	-1.035826995	0.363891048	-2.846530576	0.004419848
Fcf1	54592.35048	-1.034904683	0.380865872	-2.717241842	0.006582849
Rpl31-ps11	388.5364809	-1.031512916	0.356899504	-2.89020552	0.0038499
Mybbp1a	2780.479408	-1.023598035	0.339765933	-3.012656466	0.002589719
Cct4	12399.07306	-1.019199496	0.346667703	-2.939989754	0.003282231
Ppp2ca	5815.56812	-1.016626029	0.297744406	-3.414425287	0.000639167
Hspe1-ps2	129.1732961	-1.012942927	0.375754851	-2.69575476	0.007022935
Gm7459	277.8737949	-1.0021384	0.33801039	-2.964815369	0.003028646
Grpel1	14151.14447	-0.961171659	0.33690034	-2.852985124	0.004331065
Gm9118	1454.787848	-0.955528302	0.289704268	-3.298288659	0.000972761
Evi2a	5689.695975	-0.955383086	0.343851085	-2.778479194	0.005461401
Dnajc9	3883.400135	-0.955046773	0.344534194	-2.77199416	0.005571404
Polr2g	24899.87716	-0.935051543	0.313839145	-2.979397436	0.002888159
Use1	12231.75519	-0.906542755	0.277681643	-3.264683779	0.001095863
Foxn2	4296.944636	-0.898491293	0.329699478	-2.725182638	0.006426593
Rbm8a	26856.97789	-0.897127159	0.291369188	-3.079004899	0.002076933

Gm12856	266.6343783	-0.873452569	0.300082519	-2.910707934	0.003606109
Tma16	12244.09511	-0.867612027	0.243311197	-3.565853264	0.000362674
Ube2n	43219.42392	-0.823990725	0.309358738	-2.663544376	0.007732221
Shcbp1	21152.87088	-0.795727292	0.289659431	-2.747113359	0.006012235
Mrpl18	36302.59096	-0.732865213	0.254184675	-2.883199834	0.003936576
Acin1	1791.975709	-0.719154849	0.255974108	-2.809482781	0.004962117
Snx2	51712.51851	-0.691375495	0.216270763	-3.196805177	0.001389587
Vdac3	29664.94489	-0.661772773	0.223191947	-2.965038759	0.003026448

padj
5.05E-15
3.33E-06
1.03E-15
0.00149506
0.001777073
0.004020143
0.0046437
0.005289492
0.009582717
5.98E-05
5.09E-06
0.01486243
0.014349854
0.045181262
4.07E-05
1.36E-13
0.000642505
0.005493483
0.00279152
0.013606582
0.032148671
0.002477145
0.001179745
0.003237095
0.041853934
0.006965649
0.000406993
7.22E-05
0.002944631
0.04734543
0.030186051
0.042047515
0.002556459
0.001736834
0.036119244
0.003611648
0.020412416
0.000104132
0.000404852
0.04866511
0.003110898
0.029333484
2.14E-09
0.025465146
0.024863668
0.000319854
0.028289913
0.037101565

0.047569645
2.10E-05
0.014687937
5.64E-08
0.017809489
0.009472645
0.000319703
0.003002466
0.047580353
0.022091611
0.030051432
8.48E-05
7.98E-06
0.003639738
0.001824064
1.15E-05
0.01844597
6.48E-07
0.014100635
0.000362992
0.000256428
0.000125073
0.046511239
0.005841691
0.019419054
0.004359072
0.022017696
0.010121455
0.020465317
0.04897987
9.25E-06
0.034499103
0.004406909
0.000589047
0.003306087
0.003596045
0.011669519
0.011150444
0.00354342
0.000601771
0.000696341
0.008713889
0.005870666
0.032977121
0.022146002
0.042851569
0.042209548
0.048315275
0.026310794
0.009063119

0.037926779
0.000496587
0.001638471
0.001921383
0.016526299
0.016028142
0.000102888
0.01917869
3.98E-05
0.007399289
0.045217037
0.00101216
0.013460555
0.019609984
0.001902376
7.03E-07
0.013878858
0.003032946
0.005568447
0.005411291
0.01235039
0.010356137
0.008519808
0.000959582
0.001236748
0.001972898
0.038640165
4.88E-06
0.046360907
0.025352847
5.98E-05
0.006558552
0.013317322
0.021590144
0.000629308
0.000959582
0.015675238
0.01764692
0.006934069
0.043434359
1.76E-16
0.036119244
0.008215735
0.041573438
0.012926984
0.015014848
0.042851569
0.006570462
0.036671052
0.035669512

0.000522609
1.59E-05
0.010563751
0.019405221
0.038927399
2.54E-05
0.038674467
0.00057185
0.022244445
0.048070991
0.015978228
0.000459453
0.003968658
0.009986551
0.004597038
0.033274075
0.029059505
0.029602575
0.012335749
2.63E-05
0.003384113
0.012734005
0.005364392
0.044472821
0.000329052
0.041136506
0.010702389
0.002556459
0.021536499
0.018780792
0.000172675
0.046511239
0.009472645
0.017033935
0.025352847
0.002001249
3.83E-10
0.014097016
0.001623534
0.008772809
0.019475248
0.0390669
0.001457548
0.002576283
0.006324416
0.037336318
0.000145836
0.038123105
0.021825941
0.023726666

0.047793079
0.048575748
0.012025061
0.004501117
0.041459774
0.008263054
0.031069718
0.021825941
0.01983627
0.014388026
0.039177242
0.027938635
0.015749757
0.016324446
0.013885557
0.03881713
4.98E-06
0.006066585
0.032661883
0.045217037
0.009343233
0.043690798
0.030051432
0.028013358
0.004683061
0.001511981
0.044725371
0.004996126
0.030438167
0.023433934
0.039716014
0.015312854
1.20E-05
0.01438014
0.048307695
0.049661891
0.004136395
0.012194927
0.036276657
0.039279108
0.047240765
0.006827328
0.005610638
0.026648709
0.02186593
0.013092585
0.00074158
0.026618585
0.002944631
0.013456468

0.000867848
0.01235039
0.015354597
0.048362231
0.003364041
0.000221075
0.046904871
0.000416633
0.03142988
4.40E-05
0.019783885
0.005503196
0.01438014
0.031068326
0.006283967
0.009319008
0.027502371
0.005140195
0.003377505
0.00162702
0.006511919
0.032876266
0.010336442
0.034499103
0.029069184
0.027445899
2.11E-05
0.040058679
0.040258774
0.007177968
0.01681924
0.038927399
0.028521903
0.033338254
0.032722348
0.044386266
0.029429776
0.021590144
0.025989302
0.007105217
0.04656901
0.024505556
0.03222617
0.009954159
0.038822355
0.039308821
0.023497236
0.010963818
0.043690798
0.018079449

0.028013358
0.004556232
0.049908893
0.041502641
0.029983994
0.035981426
0.013155768
0.024501156
