# Supplementary material for: CSF1R marks a subset of foetal haematopoietic multipotent progenitor cells with acute myeloid leukaemia propagation properties
Source: Leukemia. 2026 Jan 16;40(3):540–52. doi: 10.1038/s41375-025-02856-4 (PMC12960200; doi:10.1038/s41375-025-02856-4)
Supplement: Supplementary file 4 — Supplementary Table 3 [file 41375_2025_2856_MOESM4_ESM.pdf]

genes significantly upregulated in CSF1R+LMPPs vs CSF1R-LMPPs					
gene symbol	baseMean	log2FoldChange	lfcSE	stat	pvalue
Inpp4b	2200.975145	14.02385122	1.663577127	8.429937509	3.46E-17
Cers4	1660.394041	13.61726611	1.337712805	10.17951391	2.45E-24
Zfp773	1601.734212	13.56534389	1.635630002	8.293650687	1.10E-16
Chil5	1298.608276	13.26274525	1.254261897	10.57414347	3.93E-26
Prg2	1295.213166	13.25895028	1.630483101	8.131915177	4.23E-16
Armcx1	1230.030338	13.18446942	1.27164369	10.3680532	3.47E-25
Fer	1116.554803	13.04475183	1.540536476	8.467668265	2.50E-17
Tek	1024.212826	12.92021867	1.4627208	8.833003997	1.02E-18
Gap43	1014.559582	12.90648384	3.041578848	4.243350078	2.20E-05
Crispld1	1004.123136	12.89161549	1.947019263	6.62120593	3.56E-11
Tmem106c	987.4057187	12.86742701	1.434703819	8.968699209	3.00E-19
Lgalsl	882.2420588	12.70493185	1.573084159	8.076447645	6.67E-16
Jchain	862.7700644	12.6727231	1.464405598	8.653834104	4.98E-18
Esam	856.9087941	12.66291191	1.480051514	8.555723763	1.17E-17
Nbea	827.4101635	12.61234321	1.597636165	7.894377632	2.92E-15
Abcf3	815.6018246	12.59172182	1.510764891	8.334666697	7.77E-17
Gm41077	793.5212295	12.55201881	2.749125496	4.565822414	4.98E-06
Fut10	791.6995034	12.54876261	1.614927622	7.770479887	7.82E-15
Igll1	768.8803806	12.50649391	2.8342856	4.412573632	1.02E-05
Zfp759	751.4305499	12.47338773	1.867518928	6.679122523	2.40E-11
1810073O08Rik	741.5235312	12.4542263	1.54521101	8.059887111	7.64E-16
Il15ra	713.1337319	12.39804071	1.315988934	9.421082801	4.46E-21
6430511E19Rik	709.5150536	12.39056407	1.601289223	7.737867645	1.01E-14
Gem	675.9787792	12.32077124	1.570713687	7.844059261	4.36E-15
Gm15261	667.9913589	12.30357467	1.354931239	9.080589713	1.08E-19
Sytl4	661.6760175	12.28988029	2.720008135	4.518324829	6.23E-06
Lrrc49	660.3137227	12.28688972	1.83766713	6.686134567	2.29E-11
Sox11	637.3362185	12.23578686	2.346613571	5.214231696	1.85E-07
5830405F06Rik	632.2554008	12.22428046	1.465201865	8.34306914	7.24E-17
Myct1	621.8319573	12.20038834	1.32418191	9.213528935	3.16E-20
Galm	614.6422934	12.18348367	1.631215453	7.468960428	8.08E-14
Art4	593.9393977	12.13415152	1.287529094	9.424370737	4.33E-21
Hmgxb3	586.1825076	12.1150693	1.443328464	8.393840765	4.71E-17
Gm37893	566.6847296	12.0662659	1.698862612	7.10255545	1.22E-12
Cd79a	561.2396429	12.05252471	1.285456029	9.376069221	6.85E-21
C030034L19Rik	551.9483122	12.02834829	1.525539549	7.88465189	3.15E-15
Gria2	551.4400712	12.02692608	2.493774592	4.82277994	1.42E-06
Wdr48	547.3907748	12.01633315	1.400558471	8.57967261	9.51E-18
Slc15a2	540.518761	11.9981062	2.711165297	4.425442526	9.62E-06
AW112010	537.6072237	11.99031564	1.612980786	7.433638231	1.06E-13
Kng1	534.5110828	11.98194463	2.78248872	4.306197017	1.66E-05
Sgce	529.5349719	11.9686162	1.570031764	7.623168185	2.48E-14
Gm11346	526.7393326	11.96081988	1.377542603	8.682722302	3.86E-18
Pef1	518.7368353	11.93883007	2.086471136	5.722020238	1.05E-08
Mettl22	517.5646475	11.9354936	2.758211919	4.327257639	1.51E-05
Gm35035	516.8871306	11.93354791	1.299171154	9.185508672	4.10E-20
Gramd1c	514.8243165	11.92789303	1.764095526	6.761477969	1.37E-11
Pdzk1ip1	512.4738493	11.92117133	1.44410583	8.255053808	1.52E-16

NA	501.9402104	11.89123524	2.8235797	4.21140414	2.54E-05
Gm34703	499.9352171	11.88557831	1.423569085	8.349140507	6.88E-17
Det1	494.9642406	11.87109235	2.706605911	4.385970008	1.15E-05
Pdcd1lg2	486.4540764	11.84602892	1.737507898	6.817827379	9.24E-12
Eya1	486.0568926	11.84488205	2.72105086	4.353054265	1.34E-05
Gm48614	485.4314053	11.84295447	1.494595446	7.923852904	2.30E-15
Mterf4	484.0820966	11.83901999	2.774139145	4.267637406	1.98E-05
Srxn1	483.1759581	11.83626013	2.831964896	4.179522192	2.92E-05
Gdpd1	481.6823565	11.83180868	2.427026065	4.875023325	1.09E-06
Zhx3	478.4927133	11.82229184	1.487904867	7.945596594	1.93E-15
2810414N06Rik	477.0266596	11.81782516	2.088740825	5.657870529	1.53E-08
Tbxas1	457.1079026	11.75630821	2.741255412	4.288658459	1.80E-05
Zfp867	455.6233627	11.75172172	1.639735821	7.166838448	7.67E-13
Vpreb3	445.4671545	11.71909296	1.613271447	7.264179243	3.75E-13
Gm36933	436.9732746	11.69122637	1.555158156	7.517708938	5.57E-14
Pglyrp1	433.7301667	11.68074212	1.332495156	8.766067229	1.85E-18
Stx2	431.7302373	11.67382619	2.98672428	3.908571765	9.28E-05
Gm40124	428.0836208	11.66156868	1.518599126	7.679161985	1.60E-14
Pdlim7	427.9179415	11.66105567	2.110040363	5.52646095	3.27E-08
Gm42480	418.2394543	11.62810129	2.714138607	4.284269513	1.83E-05
Gab1	418.046643	11.62742731	1.514259154	7.678624416	1.61E-14
Rfxank	417.5844794	11.62580571	1.375660108	8.451074242	2.89E-17
Tnfrsf18	413.421913	11.61151511	1.262378764	9.198122967	3.64E-20
Rapgef5	404.6902806	11.58065876	2.101829551	5.509799191	3.59E-08
Vwa8	403.6051622	11.57676553	2.214706021	5.227224478	1.72E-07
Scrn3	401.8725247	11.57064004	1.614568884	7.166396033	7.70E-13
H2bc21	401.4079347	11.5689006	2.05953501	5.617239106	1.94E-08
Gm17529	399.9948765	11.56366935	1.929908588	5.991822321	2.08E-09
Gtdc1	399.5970043	11.56249576	1.290116254	8.962367325	3.18E-19
Gm43568	398.9453035	11.56005116	1.690438287	6.83849345	8.00E-12
Gm50094	393.0138061	11.53835498	2.700927547	4.271997222	1.94E-05
Fbxo32	391.3716641	11.53223166	1.75968916	6.553561801	5.62E-11
Hlf	2909.76789	11.49154993	1.083969512	10.60135899	2.94E-26
Plxna4os1	379.5425011	11.48806412	1.535963702	7.479385161	7.47E-14
Cdin1	378.3595527	11.48359518	2.28091445	5.034645287	4.79E-07
Edaradd	375.3906403	11.47216673	2.691687256	4.262072684	2.03E-05
Zc3h6	371.7332655	11.45793061	1.629172776	7.032974515	2.02E-12
Trim6	370.6635457	11.4540255	1.657724208	6.909487988	4.86E-12
Sufu	370.6315753	11.45367094	1.779650015	6.435912028	1.23E-10
Lrrk2	368.793146	11.44651799	1.997940809	5.729157709	1.01E-08
2900092N22Rik	363.1561048	11.42430365	1.778426345	6.423827267	1.33E-10
Zfp949	360.3950159	11.41336066	2.712994464	4.206923681	2.59E-05
Vopp1	359.6503181	11.41029456	2.037332894	5.600603901	2.14E-08
Gm44164	351.4658116	11.37710141	2.748734143	4.139033031	3.49E-05
Gm42547	349.5985775	11.36939771	2.222639117	5.115269332	3.13E-07
Gcsam	346.3903118	11.35618185	2.751579492	4.127150199	3.67E-05
Atg13	345.5473405	11.3526981	2.22175072	5.109798319	3.23E-07
Rps6kc1	345.3093861	11.35159595	2.797895609	4.057190668	4.97E-05
Ankrd29	342.7819898	11.3409769	3.015375821	3.761049227	0.000169202
Myl9	338.6844421	11.32363799	2.147476139	5.272998282	1.34E-07

Lancl3	332.5131504	11.29720822	2.352003436	4.803227769	1.56E-06
Gm57488	330.8741979	11.28996419	3.032729846	3.722706856	0.000197098
Kctd12b	329.685763	11.28483221	1.96566341	5.740978926	9.41E-09
BC016579	327.5241088	11.27546983	1.717796829	6.563913519	5.24E-11
Ifit2	1218.113019	11.26780097	1.356021998	8.30945293	9.61E-17
Procr	323.8012642	11.25883122	2.791350523	4.033470941	5.50E-05
Il5ra	319.6663104	11.2403461	2.707055857	4.152240178	3.29E-05
6030442K20Rik	317.6328058	11.23108169	2.750589992	4.083153695	4.44E-05
5830428M24Rik	317.1060254	11.22870532	2.912309944	3.855601064	0.000115446
Tceal1	316.7134845	11.22688519	2.781882752	4.035714727	5.44E-05
Pcyt1b	316.5337241	11.22604401	2.702355096	4.154170569	3.26E-05
Pde8a	314.9174821	11.21868364	2.809777265	3.99273059	6.53E-05
Gm43359	313.8809368	11.21399286	2.703193227	4.148424445	3.35E-05
Mindy1	310.3596715	11.19778305	2.128542961	5.260773805	1.43E-07
Bcas3	309.1635295	11.19225146	1.639062598	6.828446623	8.58E-12
Trp53inp1	307.0013666	11.1820277	2.160419476	5.175859516	2.27E-07
A630052C17Rik	306.5612654	11.17994713	2.989518599	3.739714858	0.000184229
Mr1	305.9147719	11.1770516	1.414556415	7.901453404	2.76E-15
Efna5	305.8184124	11.17645088	2.701726272	4.136781361	3.52E-05
Gm7435	305.3248903	11.1742122	2.079620223	5.373198471	7.74E-08
Pcsk7	304.3052353	11.16946575	1.544414856	7.232166737	4.75E-13
Acot11	303.572874	11.16576923	2.148264392	5.197576832	2.02E-07
Fsip1	303.2033648	11.1639413	1.981401484	5.634366074	1.76E-08
Rwdd3	298.6987023	11.14254502	2.075395486	5.368877929	7.92E-08
Septin4	292.6063484	11.11270327	2.142750905	5.186185313	2.15E-07
Cd200	291.4410577	11.10698597	2.863872944	3.878309615	0.000105185
Rad9b	291.4363214	11.10681069	1.589340386	6.98831464	2.78E-12
Cracd	287.7497648	11.08860824	2.266637938	4.892095052	9.98E-07
9430053O09Rik	282.912371	11.06405739	2.779345508	3.980813957	6.87E-05
Gm42484	281.8345185	11.05863996	2.69876797	4.09766237	4.17E-05
Anxa9	281.6699797	11.05774936	2.247981881	4.918967297	8.70E-07
Gm35551	275.4007084	11.02512916	1.410833949	7.814618558	5.51E-15
Krbox5	274.0771787	11.01829137	2.815812212	3.913006459	9.12E-05
Dync2li1	273.0990434	11.01321949	2.758900816	3.991886704	6.55E-05
Zfp582	272.4567839	11.00981606	2.692467797	4.08911708	4.33E-05
Gm37818	270.5479221	10.99986613	1.578694311	6.967698596	3.22E-12
Ubxn11	270.2188444	10.99808222	1.730090348	6.356940973	2.06E-10
Nid2	269.9867625	10.99656695	2.987696827	3.680616737	0.000232671
Rcan3	269.5339187	10.99417375	2.192076795	5.015414503	5.29E-07
Gm50069	267.4874972	10.98319575	2.714181252	4.046596278	5.20E-05
Muc13	265.6337748	10.97322394	2.928227398	3.747394738	0.000178681
Ap3m2	264.8945691	10.96917112	3.032655065	3.617019041	0.000298015
Zfp763	2856.446909	10.96642191	0.875211148	12.53002996	5.11E-36
Fbxo17	263.9423749	10.96400182	2.78924003	3.930820476	8.47E-05
Pkd2	260.7435131	10.9463538	3.183014951	3.438989124	0.000583891
Plac1	259.7067113	10.94057206	2.863937055	3.820116101	0.000133389
Acy3	259.21594	10.93798322	2.106265162	5.193070379	2.07E-07
Emcn	3097.157856	10.93612725	1.279537389	8.546938406	1.26E-17
Gm33104	257.074016	10.92578497	1.618357424	6.751156953	1.47E-11
Smpdl3a	941.0304068	10.92561343	1.235336278	8.84424235	9.22E-19

Ctla2b	255.2958257	10.91577107	1.661558383	6.569598264	5.05E-11
B4galt3	251.1473266	10.89250937	1.504386587	7.24049886	4.47E-13
Prkca	249.7535778	10.88431074	2.225279719	4.891210149	1.00E-06
Dach2	249.1802047	10.88087381	1.571023014	6.925979898	4.33E-12
0610040B10Rik	248.6516181	10.87792547	2.700056461	4.028777038	5.61E-05
Frmd6	248.384302	10.87634825	2.721405004	3.996593023	6.43E-05
Tcerg1l	246.8517772	10.86731649	2.984505393	3.641245387	0.000271322
Eya2	246.7224742	10.86669006	2.681485897	4.052488237	5.07E-05
Tmem71	916.0857511	10.8399552	1.438400792	7.536115979	4.84E-14
Ero1b	241.8757156	10.83817868	1.900221531	5.703639549	1.17E-08
Ranbp17	241.2845172	10.83449692	2.866992334	3.779046351	0.00015743
Invs	240.8795321	10.83221567	1.620485304	6.68455039	2.32E-11
Fam135a	240.2009328	10.82807941	2.161365054	5.009833665	5.45E-07
Spns2	715.6978545	10.82066603	1.852043127	5.842556186	5.14E-09
Nhsl2	238.4476881	10.81736206	2.197904204	4.921671308	8.58E-07
2310022B05Rik	236.8331756	10.8074824	1.534716341	7.042006473	1.89E-12
Pim2	235.761751	10.80097037	1.772831972	6.092495254	1.11E-09
Gm28874	234.2962311	10.79209616	1.609290008	6.706122644	2.00E-11
4930556M19Rik	233.6365874	10.7880945	2.074458757	5.200438168	1.99E-07
Simc1	232.5893318	10.78175039	1.744162165	6.181621532	6.34E-10
Rtl8c	230.1442502	10.76612883	1.425502661	7.552514019	4.27E-14
Fzd6	229.4994276	10.76228225	2.688507705	4.00306915	6.25E-05
Gm37238	228.7546552	10.75739059	1.533221307	7.01620212	2.28E-12
Zfp189	696.2620968	10.7516228	1.643430008	6.542184791	6.06E-11
Flrt2	226.1947857	10.74125193	2.924498523	3.672852574	0.000239858
Rps6ka6	223.0373295	10.72105476	2.719440839	3.942374699	8.07E-05
NA	219.957867	10.70081542	1.542170503	6.93880177	3.95E-12
Lrrc39	219.3708286	10.69718392	2.739851207	3.904293741	9.45E-05
Vangl1	218.8655011	10.69376888	2.72340463	3.92661772	8.61E-05
Mfap2	218.1488602	10.68896034	1.428088443	7.484802771	7.17E-14
Pias4	217.2338727	10.68304316	2.694843562	3.964253553	7.36E-05
Luzp1	216.3856409	10.67744573	1.342760724	7.951860329	1.84E-15
Ankrd50	216.3833237	10.67734219	3.031887813	3.52168116	0.00042882
Gm2788	211.8276663	10.64673941	2.163259443	4.921619296	8.58E-07
Fuom	210.1077943	10.63492754	2.668603789	3.985202893	6.74E-05
Gm10658	209.7948586	10.63266898	2.767357446	3.842174053	0.000121949
NA	208.7589916	10.6255334	1.806627752	5.881418232	4.07E-09
Gm37645	208.2592028	10.62219005	2.676698467	3.968392474	7.24E-05
Gm43571	208.0960667	10.62092463	3.059597456	3.471347061	0.000517854
Rufy4	207.7790502	10.61882757	1.68102481	6.316877363	2.67E-10
Il9r	206.9714216	10.61318735	2.041545727	5.19860379	2.01E-07
Dhrs11	206.6520362	10.61096941	1.867107651	5.683105309	1.32E-08
Ppm1j	206.0232733	10.60659198	3.083551504	3.439732389	0.00058229
1700040D17Rik	205.0178547	10.59941017	2.961104157	3.579546551	0.000344191
Pgbd1	203.7787638	10.59080214	2.66443394	3.974878859	7.04E-05
4833407H14Rik	202.5298143	10.58191456	2.689340374	3.934762093	8.33E-05
Nxf2	201.636094	10.57551627	3.058785211	3.457423631	0.000545367
Gm4633	199.5850137	10.5606688	2.896022731	3.646611158	0.000265722
Timp2	198.6274982	10.55374397	3.18128213	3.317449865	0.000908432
Gm3235	198.2109705	10.5511242	1.42768087	7.390394048	1.46E-13

Ddhd1	197.3383822	10.54456161	1.892719129	5.571118001	2.53E-08
Unc13b	197.3042675	10.54425201	2.467731932	4.272851468	1.93E-05
Zfp558	197.266135	10.54395356	2.692735815	3.915702946	9.01E-05
D930020B18Rik	195.7235581	10.53283477	1.629346083	6.464455207	1.02E-10
Fam221a	194.6000036	10.5243317	2.762818996	3.809272961	0.000139376
Deptor	194.3864598	10.52282002	2.250465451	4.675841622	2.93E-06
Slc2a4rg-ps	869.8028491	10.51789987	2.481808555	4.237998071	2.26E-05
Ccdc17	193.6787053	10.51740572	3.090854932	3.402749709	0.000667114
Kyat1	605.3253736	10.51667622	1.475165498	7.129150075	1.01E-12
Gm10603	193.3534204	10.51483723	1.830337962	5.74475176	9.21E-09
4931439C15Rik	192.5468667	10.50900148	2.708008267	3.880712483	0.000104151
Mecom	3941.335199	10.50477874	1.372149477	7.655710197	1.92E-14
Il1rl2	191.7851929	10.50330829	1.938921488	5.417087978	6.06E-08
Slc12a7	191.0992891	10.49819687	2.044000596	5.136102647	2.80E-07
Mon1a	191.0925858	10.49793799	2.858518787	3.672509706	0.00024018
Grap	189.5317283	10.48625917	2.727743443	3.844298187	0.000120898
Gm44428	189.0877591	10.48262741	1.514503101	6.921496167	4.47E-12
Srgap1	188.0733199	10.47508083	2.796533073	3.745738224	0.000179864
Wdr59	187.7123726	10.47235086	2.197299811	4.766009082	1.88E-06
Gm46781	187.2260222	10.46879596	1.622468703	6.452386992	1.10E-10
Zfp248	187.2012781	10.46822422	1.530991521	6.837545522	8.06E-12
Gm45203	186.7482698	10.46489406	2.670481838	3.918728787	8.90E-05
Ndn	186.6863056	10.46422537	1.730681218	6.046304343	1.48E-09
Gm13431	186.3521885	10.46175581	2.706352417	3.865629527	0.000110803
Gm37949	186.2819581	10.46124106	3.022413593	3.461220887	0.000537731
Drc7	185.3107044	10.45376678	2.713593617	3.85237005	0.00011698
Adam22	184.8066825	10.44972505	2.772163925	3.769519167	0.000163562
Lppos	184.5455349	10.44751742	1.539430721	6.786610969	1.15E-11
Pcbp3	184.4810227	10.4472459	2.757519789	3.788638594	0.000151475
Ciart	184.1696761	10.44480273	2.86350867	3.647554081	0.000264749
4930434J08Rik	184.0126282	10.44347176	2.910466851	3.588246251	0.00033291
Gm13522	183.610104	10.4402394	1.65178598	6.320576353	2.61E-10
Gm42639	183.4647338	10.43941904	1.991446554	5.242128652	1.59E-07
4930520O04Rik	182.6248758	10.43269293	2.871401407	3.633310516	0.000279808
Gm43300	182.0820206	10.42824732	2.881184557	3.619430521	0.000295252
Gm11415	180.8147285	10.41811494	1.62003101	6.430812052	1.27E-10
Arl15	179.0725527	10.40448999	1.940084879	5.362904529	8.19E-08
Zfp747	178.6832814	10.40104423	1.891330843	5.499325658	3.81E-08
A830005F24Rik	178.100848	10.3964728	2.715318764	3.828822212	0.000128758
Enpp5	177.985877	10.39556973	2.570781549	4.043739046	5.26E-05
3222401L13Rik	177.9164843	10.39500319	2.721751529	3.819232977	0.000133867
Gm29946	177.6881727	10.393013	2.905368179	3.57717589	0.000347326
Ighv1-74	175.5350857	10.37564359	2.256398893	4.59831975	4.26E-06
Bloc1s6os	174.4427664	10.36642072	2.057241248	5.038991286	4.68E-07
Dnai4	173.6908196	10.36046017	2.126656046	4.871714064	1.11E-06
Map4k5	173.2904443	10.35696437	2.780946502	3.724258758	0.00019589
Cited1	173.0838688	10.3552652	2.674272939	3.872179629	0.000107866
Ighv1-76	172.6378004	10.35154533	2.713023035	3.815502191	0.000135906
Plxna3	172.0483539	10.34664259	2.771817442	3.732800879	0.000189362
Tctn2	171.5909968	10.34271596	3.031255773	3.412023508	0.000644826

Cbx2	171.2767158	10.34015103	2.489230141	4.153955418	3.27E-05
NA	170.5757571	10.3340869	3.005789007	3.438061313	0.000585895
Snx32	170.2769489	10.33177332	2.067420709	4.9974218	5.81E-07
Gm45747	491.1422012	10.32181405	2.383278997	4.33092981	1.48E-05
Tmc6	169.013863	10.32096357	2.66991826	3.865647772	0.000110795
Gm36989	167.7432018	10.31008733	1.722840127	5.984355232	2.17E-09
NA	167.123662	10.30466934	2.897446889	3.556465309	0.000375878
Gm20696	165.8582964	10.29375567	2.220709322	4.63534582	3.56E-06
Tspan7	1317.91329	10.28995276	1.539989815	6.681831702	2.36E-11
Pemt	165.2304472	10.28825186	2.887544257	3.562976338	0.000366674
Igf1	1448.54467	10.28768581	1.062010062	9.686994666	3.42E-22
Tgfbi	165.1345009	10.28746578	2.670258972	3.85260976	0.000116866
I730030J21Rik	164.5423018	10.28206066	1.455090595	7.066268376	1.59E-12
Dio2	164.5130815	10.2818303	2.322541989	4.426972839	9.56E-06
Ppip5k1	164.4230453	10.2811991	2.749132612	3.739797437	0.000184169
Cenpt	1417.761317	10.28101515	1.138089832	9.033570868	1.66E-19
NA	164.0387155	10.27784893	2.661564106	3.861582333	0.000112655
Hm629797	163.8975598	10.27658125	2.704491357	3.799820332	0.000144801
Cyp27a1	163.8219369	10.27588895	2.870347982	3.580015043	0.000343574
Mtmr7	163.8156669	10.27576578	2.773438531	3.705063468	0.000211338
A230087F16Rik	162.6449682	10.26555268	2.843157233	3.610617296	0.000305469
Ccp110	1467.970876	10.26523242	1.836472786	5.589645813	2.28E-08
Rgs7bp	162.3116883	10.26244279	2.787865137	3.681111633	0.000232219
Gm43340	161.9875315	10.25978062	1.311202864	7.824708821	5.09E-15
1500015A07Rik	161.7902824	10.25795924	2.716239707	3.776529449	0.000159029
Rbp2	161.3976685	10.25436825	2.936459259	3.492085994	0.000479264
Gm43915	161.051681	10.2513584	2.773779554	3.695808625	0.000219188
Gm29642	159.7711736	10.23983268	2.802655131	3.653618515	0.00025857
Dennd2d	1245.886855	10.23687194	1.259924665	8.124987333	4.47E-16
Pnpla6	159.0125815	10.23275882	2.146354655	4.767506061	1.87E-06
Aldh4a1	157.7482243	10.2216038	1.861553287	5.490900463	4.00E-08
Zdhhc16	157.276973	10.21706765	2.80932302	3.636843316	0.000276
Plekhg1	156.9985187	10.2147114	1.908785814	5.351418336	8.73E-08
Klrb1c	156.2182782	10.20739927	2.715697023	3.758666443	0.000170821
Aqp1	155.9139406	10.20439889	3.033642713	3.363744467	0.000768927
NA	155.8817544	10.20410734	2.828362597	3.607779056	0.000308829
2410018L13Rik	155.3900467	10.19970721	2.686200493	3.79707592	0.000146413
Cfap92	155.3418074	10.19933074	2.294196456	4.445709395	8.76E-06
Mamdc2	154.9621917	10.19577112	1.849485064	5.512762075	3.53E-08
Trbv13-2	154.2272198	10.18854396	1.729952194	5.889494515	3.87E-09
Snx33	153.9477114	10.1862748	2.739587362	3.71817849	0.000200664
Zscan20	153.785229	10.18468927	2.601759405	3.914539233	9.06E-05
Gm15991	152.9809676	10.17714026	2.702013206	3.766502784	0.00016555
Zfp78	152.248791	10.17023193	3.132284153	3.246905909	0.00116667
Slc25a27	152.2019595	10.16991538	1.916397201	5.306788895	1.12E-07
Pard3b	151.7474599	10.16544602	2.726466571	3.728432296	0.000192675
0610010K14Rik	151.5402442	10.16353815	2.72938804	3.723742467	0.000196291
Parp12	151.503785	10.16327184	2.164619059	4.695178027	2.66E-06
Prss3b	151.225358	10.16064095	2.018965338	5.032598014	4.84E-07
Rfx5	296.1657861	10.15716395	1.516507354	6.697734716	2.12E-11

NA	150.0425042	10.14918635	2.681512667	3.784873542	0.000153787
Trpt1	150.0116869	10.14871752	2.52904408	4.012866995	6.00E-05
Gm10643	149.8198789	10.14702535	2.680374072	3.785675089	0.000153292
Sh2d5	555.5838161	10.14098471	1.328250558	7.63484318	2.26E-14
Hif3a	149.0683668	10.1399583	1.933253394	5.245022887	1.56E-07
Trak1	148.986766	10.1389247	1.745538247	5.8084804	6.30E-09
4933406J10Rik	148.4173243	10.13333703	2.751334709	3.683062261	0.000230449
Cyp2r1	148.0840853	10.13020359	2.794883202	3.624553463	0.000289461
Cd4	148.0455853	10.12979393	1.727145975	5.865047933	4.49E-09
Gucy1a1	147.6320314	10.125744	1.524288667	6.642930709	3.08E-11
Rbm44	584.6356361	10.1237237	2.061864732	4.909984413	9.11E-07
Cgnl1	146.0960563	10.11053374	2.393369519	4.224393123	2.40E-05
Clec10a	145.9232387	10.10900255	2.698941861	3.745542908	0.000180004
Zdhhc2	145.6226535	10.10606401	2.931763194	3.447094238	0.000566651
Ccdc39	144.2719933	10.09254435	2.801448038	3.60261701	0.00031503
C230071H17Rik	144.1096884	10.09097097	2.682943294	3.761157008	0.000169129
Gm44027	143.2472541	10.08233144	2.745157918	3.672769196	0.000239936
Gm38042	142.1817526	10.07149832	3.069243055	3.281427419	0.001032831
St3gal2	546.6903372	10.07000851	1.287366979	7.822174003	5.19E-15
Flrt3	570.9606397	10.06960416	2.502794066	4.023345065	5.74E-05
Pigb	272.3691619	10.06483813	2.392574542	4.206697829	2.59E-05
Gm17473	140.8936586	10.0582781	1.976358521	5.089298321	3.59E-07
Epor	140.7800605	10.05710558	2.866718467	3.508229253	0.0004511
Suox	139.8473034	10.04766614	2.667792974	3.766284054	0.000165695
Gm47572	139.6909478	10.04607635	2.670271306	3.762193125	0.00016843
Ovca2	139.3740073	10.04262138	2.05314897	4.891326214	1.00E-06
Creb3l1	139.2243094	10.04104661	2.940760685	3.414438537	0.000639136
Zer1	139.12091	10.04027576	2.066094743	4.859542762	1.18E-06
Gprc5b	139.0354326	10.03899524	2.090068229	4.803190201	1.56E-06
Fbxo31	138.4353178	10.0328725	1.946468935	5.154396414	2.54E-07
Tsbp1	137.9020635	10.02727783	2.912459666	3.442889851	0.000575534
Zfp618	137.6722707	10.02483768	1.690350289	5.930627366	3.02E-09
Gm43788	137.3053948	10.02122167	2.681295144	3.737455645	0.000185892
Sugct	136.215236	10.00986996	2.11300973	4.737256918	2.17E-06
Tmem177	546.6872972	10.00878899	2.448414673	4.087865141	4.35E-05
Gm17816	135.7226087	10.00430044	2.3647966	4.230512021	2.33E-05
Angpt2	135.2911126	9.999899066	2.963613411	3.374225204	0.000740238
Cyp4v3	135.2097499	9.999035438	2.698680841	3.705156714	0.00021126
Cyth3	135.186832	9.998784841	2.698263023	3.705637574	0.00021086
Tcim	135.0449154	9.997414943	2.024494708	4.938227253	7.88E-07
Rassf9	134.1083455	9.987228015	2.675822274	3.732395874	0.000189667
Col3a1	133.7799124	9.983492153	3.086127324	3.234957961	0.001216606
Gm43852	133.6929108	9.982561123	3.204257965	3.115404949	0.001836925
Prrg1	133.3107619	9.97860727	2.468717513	4.042020692	5.30E-05
Tnnc1	132.8382409	9.973301818	2.897475278	3.442066233	0.000577289
Sh2d2a	254.5238614	9.965950888	2.210024271	4.509430516	6.50E-06
NA	131.9640771	9.963802439	1.398877227	7.122714019	1.06E-12
Pdgfd	131.8175305	9.962356977	3.117900225	3.195213528	0.001397274
Gm38142	380.5009105	9.958018091	2.465287704	4.039292482	5.36E-05
5330432J10Rik	131.4116308	9.957887184	2.992147854	3.328006392	0.000874699

NA	131.3610882	9.957368661	2.753794241	3.615872426	0.000299338
Cacna2d1	130.9432082	9.952812258	2.089786559	4.762597507	1.91E-06
NA	129.7218704	9.939001399	1.604738874	6.193531896	5.88E-10
Prss2	129.4007167	9.935688102	2.763172239	3.59575417	0.000323453
5830418P13Rik	129.2713267	9.934203381	2.859873759	3.473651013	0.000513428
Jam3	467.2248586	9.93212691	1.17855954	8.427344203	3.54E-17
Gm48796	127.9263584	9.918873455	1.563947947	6.342201782	2.27E-10
C230035I16Rik	127.0292726	9.90901768	2.793818276	3.546765288	0.000389992
Zfhx2	126.8606465	9.906758823	1.447047017	6.846190002	7.58E-12
C1qb	125.8674747	9.895867257	2.035839861	4.860827929	1.17E-06
Rsad2	416.446533	9.888810177	1.169982214	8.452102999	2.86E-17
Gm44210	125.2108614	9.888231259	2.194339417	4.506245107	6.60E-06
Gm12185	124.7811888	9.883421424	2.010592676	4.915675631	8.85E-07
Aldh1l1	124.6910578	9.882020703	2.963543163	3.334529028	0.00085444
Enkur	124.0575301	9.87470613	1.481811758	6.663941001	2.67E-11
Ikbke	123.5073046	9.868205992	2.160844881	4.566827577	4.95E-06
Gm12828	123.4360265	9.867621023	2.813889105	3.506755474	0.000453606
Hdgfl3	123.4255499	9.867479231	2.745620816	3.593897297	0.000325768
Gm27042	123.006427	9.862522093	2.713599058	3.634480217	0.000278542
Pira2	122.8698852	9.860987166	2.780830081	3.546058866	0.000391039
Gm30948	122.7146668	9.859090403	2.319218695	4.251039553	2.13E-05
Htr2a	122.5225982	9.856786753	3.143296655	3.135811804	0.001713791
Dok4	122.3438072	9.854604838	2.853416573	3.453615897	0.000553125
Slc5a11	122.1948421	9.853149748	1.998452505	4.93038975	8.21E-07
Crim1	121.5559295	9.845431429	1.588718965	6.197088125	5.75E-10
Olfm5	121.0999109	9.839944763	2.874364298	3.423346432	0.000618552
Tecpr1	121.0682964	9.839579162	2.176781002	4.520243035	6.18E-06
Gm13940	121.0357517	9.839275167	2.68306965	3.667170984	0.000245249
Map9	121.0158478	9.838683778	1.376904116	7.145511196	8.97E-13
Gm37902	120.0896432	9.827725732	2.970252031	3.308717789	0.000937243
Dnal1	119.9103932	9.825832599	2.223432898	4.419217062	9.91E-06
Cyp4b1	119.7104014	9.823329546	2.68794845	3.654582567	0.000257601
Hdhd3	119.5339585	9.821207954	2.780560389	3.532096621	0.000412279
Tbc1d30	118.7788245	9.812126086	2.887006051	3.398720305	0.000677019
Gm26664	118.7141411	9.811214462	2.800943784	3.502824483	0.000460353
6720427I07Rik	118.6639696	9.810740617	2.7537026	3.562745162	0.000366997
Gm20467	118.5814644	9.809726629	2.739075456	3.581400654	0.000341757
Setbp1	118.2475076	9.805884542	1.738117255	5.641670325	1.68E-08
5530402G07Rik	118.1609292	9.804424139	2.928804059	3.347586231	0.000815186
Plekho2	117.5074388	9.796467955	2.027778236	4.831133789	1.36E-06
Zfp619	117.4756232	9.796181025	3.177714056	3.082776125	0.002050794
Slc20a2	117.3887656	9.79494788	2.353900603	4.161156111	3.17E-05
Fam237b	117.2578509	9.793536907	2.67215315	3.665035781	0.000247304
NA	117.1834861	9.792439583	1.921847521	5.095325968	3.48E-07
Gm15201	117.086235	9.791395661	2.689635633	3.640417141	0.000272197
Gm39556	117.0672011	9.791084677	2.867967173	3.413945867	0.000640293
Btbd9	683.1338787	9.790027779	1.3078283	7.485713359	7.12E-14
Acrbp	116.7682487	9.787429269	2.724899827	3.591849201	0.00032834
Stox1	116.6094735	9.785497552	3.215153814	3.043555027	0.002338007
Ccrl2	116.5637352	9.784967838	2.682958909	3.647080768	0.000265237

Gm15550	116.1371942	9.779675413	2.674232891	3.657002143	0.000255182
Psrc1	710.0187562	9.778963099	1.097772143	8.90800806	5.20E-19
Scarf1	116.0183237	9.777933482	2.125679317	4.59990997	4.23E-06
Chst2	115.6034211	9.773008231	2.045111772	4.778715942	1.76E-06
Rai14	329.3413354	9.759548099	2.678434562	3.643750808	0.000268694
Zfp142	114.503538	9.759233762	2.151007316	4.537052799	5.70E-06
Itih2	114.0956146	9.754105012	2.77877136	3.510222234	0.000447732
Gm28053	113.8020954	9.749895039	1.628274444	5.987869598	2.13E-09
Nsg2	113.2121276	9.742990756	2.347578032	4.150230843	3.32E-05
Gm44148	113.1486131	9.741755597	1.541058627	6.321469819	2.59E-10
Acox3	112.858767	9.738409086	2.233031337	4.361071394	1.29E-05
Gm43729	112.8264916	9.737885772	2.797455716	3.480979419	0.000499584
Gm43336	112.7604921	9.737111317	2.689571966	3.620320051	0.000294239
Trgc1	112.463706	9.733330744	2.737597563	3.555427895	0.000377364
Dkkl1	112.4341976	9.732944384	2.764236453	3.52102454	0.000429883
Cnst	112.3262596	9.731563763	2.793567921	3.483560822	0.000494791
Dnajc28	112.3129502	9.731346631	2.694137199	3.61204568	0.000303791
Tnfsf13b	112.2108538	9.730060433	2.667075717	3.648213048	0.000264071
Mgmt	657.5909753	9.725269347	1.273281266	7.637958404	2.21E-14
Cd200r3	422.1929824	9.722313094	1.434014753	6.779785964	1.20E-11
Gm45555	1521.453549	9.721834284	1.599117067	6.079501296	1.21E-09
Zfp57	111.3415262	9.718785401	2.889459129	3.363531016	0.000769522
Nphp1	111.2182279	9.717213094	2.709130327	3.58683855	0.000334711
Dusp18	110.1682051	9.703512167	2.427657128	3.997068637	6.41E-05
B3galt5	109.8547919	9.699467077	2.697293724	3.595999572	0.000323148
Gm54030	109.740552	9.697607582	1.53193034	6.3303189	2.45E-10
Ercc3	1302.938582	9.694958339	1.186792724	8.169040931	3.11E-16
Schip1	108.9001852	9.687111991	1.625686223	5.958783347	2.54E-09
Arhgef25	108.5026667	9.681378247	2.797822107	3.460326596	0.000539521
Sh3yl1	108.0747051	9.675891132	2.687636656	3.600148521	0.000318035
Rapgef4	107.9629192	9.674418423	2.000450996	4.836118676	1.32E-06
Gm32834	107.7270157	9.671036763	2.850811814	3.392379924	0.000692883
Lysmd2	107.5950681	9.669446339	2.670103146	3.621375584	0.000293041
Zbtb17	106.6045841	9.656187764	1.98812033	4.856943324	1.19E-06
1110003F10Rik	106.4824485	9.654387505	2.851464161	3.385764983	0.000709801
Gm56717	106.4239905	9.653430176	2.870035819	3.363522543	0.000769546
Rasgrp4	105.9347595	9.647021491	2.703953072	3.567747382	0.000360063
Gm12917	105.557769	9.641910633	2.785626723	3.461307487	0.000537558
Cd19	105.3200238	9.638598593	3.109936379	3.099291245	0.001939842
Rtel1	396.9421806	9.638065058	1.409581396	6.837537075	8.06E-12
Tmem47	105.2004051	9.637003507	2.779943254	3.466618786	0.000527049
Scn2a	104.6852906	9.629877471	2.789667815	3.451979988	0.000556489
Prss36	104.3551788	9.62530006	2.735549967	3.518597786	0.000433834
Rxra	104.2356715	9.623654204	2.184939745	4.404539862	1.06E-05
Kcnb1	104.1888723	9.623380068	1.444462251	6.662257916	2.70E-11
NA	104.0834449	9.621358417	2.896619604	3.321581613	0.000895088
Lats1	1173.819329	9.619433723	1.067672119	9.009726443	2.07E-19
Lclat1	311.6316331	9.615173604	1.808170926	5.317624271	1.05E-07
Micall2	103.3549741	9.611643982	1.941333025	4.951053661	7.38E-07
Mir155hg	103.3400635	9.61097467	1.821244637	5.27714645	1.31E-07

Gm57263	102.6844721	9.601850507	2.84188185	3.378694475	0.000728309
Ighv1-80	102.3574845	9.597160135	2.2882392	4.19412452	2.74E-05
Gm15764	102.0714682	9.593340309	2.91914769	3.28634976	0.001014949
A430090L17Rik	101.9076004	9.59106472	2.730048902	3.51314759	0.000442831
Smox	101.7428374	9.588791825	1.524556842	6.289560063	3.18E-10
NA	101.5291861	9.585704627	2.741413564	3.496628438	0.000471178
Col23a1	101.4992551	9.585316305	3.090154627	3.10188889	0.001922901
Trpc6	101.3250189	9.582545803	2.30986434	4.148531858	3.35E-05
Trbv1	101.2083928	9.581202954	2.231804998	4.293028721	1.76E-05
A530064N14Rik	101.0486146	9.578880366	2.688917316	3.562355863	0.000367542
Gm45828	100.8096611	9.575511977	2.701392527	3.544657758	0.000393123
Fga	100.6565636	9.573043218	2.039833878	4.693050409	2.69E-06
Aldoc	100.2616571	9.567617234	3.09512564	3.091188645	0.001993569
Pld3	99.97692464	9.563500078	3.071779212	3.113342274	0.001849814
Rce1	99.85445754	9.561947803	1.880897737	5.083714874	3.70E-07
Gm26907	99.82316598	9.561044018	2.893326984	3.304515552	0.000951407
Gm37938	99.66366835	9.558958877	2.652448115	3.603825018	0.000313568
Dennd2a	99.53666853	9.557147716	2.693178521	3.54864991	0.000387212
Arhgef15	99.51287737	9.556734594	3.036733262	3.147044462	0.001649299
Gm45220	99.47919873	9.556290645	2.895013987	3.300948005	0.000963587
Gm42979	99.46740657	9.55617392	2.782781303	3.434036987	0.000594663
Spns3	445.2432101	9.555026243	2.427470717	3.936206595	8.28E-05
Daam1	99.00635704	9.549473656	2.699909991	3.536960005	0.000404761
Atp9b	292.7259074	9.546938472	2.009569401	4.750738375	2.03E-06
Gm29170	98.40020558	9.54057278	2.683288739	3.555552051	0.000377186
1500004A13Rik	98.20358996	9.537723342	1.961923131	4.861415409	1.17E-06
Gm37019	98.1981836	9.537566429	2.973898749	3.207091846	0.001340842
B230217O12Rik	295.9040597	9.535446319	1.77159845	5.382397078	7.35E-08
NA	97.78540416	9.531418509	3.161351623	3.014982085	0.002569944
Btg3	97.57381822	9.528723846	1.457750154	6.536596013	6.29E-11
Ehbp1	97.59373774	9.52847575	2.80433511	3.39776645	0.000679384
Gm19967	97.566869	9.52817538	3.26475223	2.918498774	0.003517213
Apoh	97.24249941	9.523378256	2.960179603	3.217162312	0.001294653
NA	96.49481159	9.512305324	3.057713378	3.110921185	0.001865047
Pcdh17	492.0188176	9.503824886	2.775732655	3.423897784	0.000617298
Neb	95.88181803	9.503312118	2.169687901	4.380036463	1.19E-05
Gm56941	95.86394423	9.502796602	3.012881054	3.154056344	0.00161018
Tnf	94.62580784	9.484277979	2.027528237	4.677753833	2.90E-06
Kank3	93.93462574	9.473683293	1.58986225	5.958807623	2.54E-09
NA	93.83187142	9.471671549	1.645297441	5.756814125	8.57E-09
Zcchc24	93.67100766	9.469111875	1.729692665	5.47444761	4.39E-08
Gnat2	93.63960046	9.468953653	2.975828831	3.181955075	0.001462845
Gm37642	93.21308567	9.462403339	2.968131321	3.188000232	0.001432604
Tmem81	92.82890568	9.456498641	2.671566969	3.539682423	0.000400609
Zbtb33	92.78715709	9.45588669	2.709259217	3.490211136	0.000482639
Il17re	92.25570325	9.447554352	2.861066507	3.302109311	0.000959607
Zfp114	91.94021536	9.442604499	2.501852956	3.774244396	0.000160493
Fmnl3	91.51752648	9.435971775	2.683001285	3.516946424	0.000436542
Gm56976	173.8397292	9.417168181	2.477621913	3.80088993	0.000144177
Gm49599	90.15152012	9.414318401	2.841930893	3.312648602	0.00092417

Glis3	89.96305456	9.411301649	2.721879627	3.457647999	0.000544913
Ighv1-77	304.5317853	9.408702132	2.147273506	4.381697116	1.18E-05
9130008F23Rik	89.71528827	9.407488609	2.122317552	4.432648922	9.31E-06
Snapc4	390.2173491	9.407401653	1.997864336	4.708728958	2.49E-06
Robo4	89.58457064	9.405214291	2.736057782	3.437505725	0.000587098
Ublcp1	359.4537521	9.404665079	2.411634399	3.899705976	9.63E-05
NA	89.46527685	9.403236904	1.78050938	5.281206046	1.28E-07
Zfp408	89.26061125	9.39990081	2.74448676	3.425012263	0.000614772
NA	88.03278586	9.379516442	1.680546069	5.581231371	2.39E-08
Jmjd4	87.86284524	9.377191548	3.070549025	3.05391364	0.002258771
Gstt1	87.82543141	9.376570953	2.138489084	4.384670945	1.16E-05
Tert	87.67621844	9.373966753	3.211690416	2.918701848	0.003514922
Tuba8	87.44073469	9.370154277	2.939225291	3.187967355	0.001432767
Abhd15	87.12048169	9.364978191	2.703596615	3.463896255	0.000532412
Ccdc57	87.05657717	9.363929481	2.781364903	3.366667017	0.000760825
Zfp267	664.617466	9.35101734	2.242604688	4.169712741	3.05E-05
Dcdc2b	86.1283819	9.348185462	2.847153689	3.283344169	0.001025833
Gm43462	86.10625919	9.348090238	2.729075818	3.425368462	0.000613966
Syne1	85.57221259	9.33899505	2.75920485	3.384668975	0.000712641
Syne2	336.8407393	9.330453253	2.015001508	4.630494428	3.65E-06
Ryr2	84.83731544	9.326568943	3.000752407	3.108076801	0.001883091
Xkr6	84.80330002	9.32608187	2.689067961	3.468146587	0.000524061
Gm37349	84.32648102	9.317819467	3.130250256	2.976701128	0.002913679
Iqcg	84.27197876	9.31719402	1.970092087	4.72931904	2.25E-06
NA	84.24086533	9.31660921	2.222185479	4.192543466	2.76E-05
Pde5a	84.23938083	9.316304406	2.839955199	3.280440624	0.001036451
Tmem19	505.3368582	9.314501639	1.17244185	7.944531864	1.95E-15
Trim41	83.8626296	9.309990627	2.689453775	3.461665976	0.000536843
NA	83.74958766	9.307946546	3.056819927	3.044977058	0.002326981
Tanc1	83.69799045	9.30711221	2.774876476	3.35406361	0.000796341
Lrch3	83.48964024	9.303702618	1.448283941	6.423949307	1.33E-10
Cxcr3	485.1487417	9.303558658	1.336950526	6.958790531	3.43E-12
Zbed5-ps	83.45544023	9.302305972	1.58677383	5.862401936	4.56E-09
NA	83.26466579	9.299659161	2.680963521	3.468774971	0.000522837
Cd55	82.79029519	9.291654719	2.041041446	4.552408642	5.30E-06
5830487J09Rik	82.76268167	9.290897223	2.693268564	3.44967351	0.000561265
Gm57320	82.70880162	9.289914821	2.85091277	3.258575611	0.00111973
Gm14858	82.60722865	9.287793506	1.876981403	4.948260803	7.49E-07
Fmo5	82.53151563	9.286914108	2.72538089	3.407565578	0.000655452
Apobr	82.51928072	9.286703025	2.91346942	3.187506607	0.001435052
Rsf1os2	82.31647161	9.282971711	3.13888238	2.95741305	0.003102322
Jakmip1	82.21845006	9.281170167	1.81536553	5.11256274	3.18E-07
Tatdn3	655.6866319	9.278431318	1.589249227	5.838248123	5.28E-09
NA	81.90079008	9.275783665	2.695191288	3.441604945	0.000578274
Mylk3	81.88671666	9.275420236	3.085001545	3.006617696	0.002641718
Pafah2	323.0718582	9.273655409	1.378996776	6.724929	1.76E-11
Slc25a24	645.1930047	9.270840058	1.395027357	6.645633156	3.02E-11
Siglece	81.54634982	9.269599522	2.719657383	3.408370326	0.000653521
2610017A05Rik	80.77127637	9.255308938	1.88185908	4.918173223	8.74E-07
Gm37024	80.66924475	9.253982486	2.905480423	3.185009409	0.001447493

Smg6	283.5762463	9.248713647	2.158573514	4.284641495	1.83E-05
Gm44321	80.30595821	9.247402202	2.728115669	3.389666467	0.000699777
NA	80.19168018	9.24489511	1.66636024	5.54795709	2.89E-08
Mfsd2b	80.16462133	9.244871078	3.082307047	2.999334893	0.002705697
Serpina3f	80.08967257	9.243718196	2.384105812	3.877226485	0.000105654
NA	79.97006843	9.2413319	3.209603593	2.87927516	0.003985904
Ghr	79.96090162	9.24118165	2.308290061	4.003475042	6.24E-05
Plod2	79.77406142	9.237805289	3.140091924	2.941890082	0.003262157
9530004M14Rik	79.68508297	9.235642038	1.624171864	5.686369922	1.30E-08
Gm26981	79.48952941	9.232588536	2.849241753	3.240366855	0.00119376
Gm27003	78.80497737	9.220457663	2.103291219	4.383823591	1.17E-05
Postn	78.42141057	9.213325247	2.45623878	3.750989244	0.000176138
Gm47583	77.7286796	9.200244342	3.169134335	2.903078056	0.003695145
Gm20682	77.63475665	9.19881351	2.378819861	3.866965154	0.000110198
Msantd3	235.3212153	9.193123345	1.690306341	5.438732093	5.37E-08
Gm17586	76.969686	9.18620884	2.695265815	3.408275647	0.000653748
Gm15903	76.6743199	9.180679633	2.799479666	3.279423582	0.001040194
Gm13562	76.29835456	9.173555229	2.736866297	3.351846321	0.000802746
Nod1	76.28214001	9.172857518	1.984505815	4.622237662	3.80E-06
NA	76.11952722	9.169661849	1.733873523	5.288541365	1.23E-07
NA	75.75283004	9.163216806	2.739946027	3.344305586	0.000824888
Eml6	75.6315766	9.160724202	2.83129949	3.235519321	0.001214217
Cep126	75.60662735	9.160280553	1.600477375	5.723467694	1.04E-08
NA	75.44900867	9.157452214	2.682986883	3.41315579	0.000642152
Kif16bos	75.06430238	9.149785853	2.795595975	3.27292854	0.001064394
Gm34758	74.82263734	9.144758685	1.510567981	6.053854444	1.41E-09
Morc4	247.757081	9.138550145	1.168334211	7.821863007	5.20E-15
Ube2e2	410.7326242	9.137043373	1.685647039	5.420496202	5.94E-08
Galnt12	74.26965313	9.134770989	2.799605675	3.262877723	0.001102871
Asap2	73.80445322	9.125573606	2.301211336	3.965552169	7.32E-05
NA	73.74896972	9.12460342	2.972836395	3.069325792	0.002145425
Ino80d	1067.399367	9.121742808	1.012797109	9.006485825	2.13E-19
Tspyl5	73.6280401	9.121623976	1.840950179	4.954845646	7.24E-07
Vdr	73.49924501	9.119710167	2.969656527	3.070964633	0.002133684
NA	73.22843552	9.114400789	2.757535201	3.305270876	0.000948846
Ptp4a1	73.20021503	9.113503059	3.235212742	2.816971799	0.004847878
Macroh2a2	72.44291564	9.098839335	2.801911684	3.247368355	0.001164775
Armc2	72.38359456	9.097649493	2.897179074	3.140175067	0.001688469
Zfp583	72.38127113	9.097332348	2.450779618	3.712015672	0.000205615
NA	72.02463992	9.090444111	2.998397022	3.03176799	0.00243126
Cpeb4	71.77335878	9.085427646	2.710505163	3.351931503	0.000802499
Ric8b	71.6312942	9.082707776	2.410865907	3.767404794	0.000164953
Nadsyn1	71.38457269	9.077548144	2.806616449	3.234338681	0.001219248
Gm45715	71.28860676	9.075380036	2.19264396	4.139012171	3.49E-05
Gm17690	71.18980454	9.073634846	2.707808616	3.350914387	0.000805452
Tbc1d4	739.2235917	9.070524026	1.732258975	5.236240168	1.64E-07
Adgre5	70.9753348	9.069323004	2.78803651	3.252942697	0.001142165
Shcbp1l	70.95958525	9.068896697	2.735749039	3.314959292	0.000916565
Gm37962	70.77644952	9.065273704	2.826441828	3.207309492	0.001339828
Gm43715	70.7265256	9.064233773	2.830517942	3.202323376	0.001363239

Nxpe5	70.61012917	9.061368125	1.592813488	5.688907201	1.28E-08
Gm42940	70.32183501	9.055707033	2.767400712	3.272278927	0.001066843
D330041H03Rik	70.28094617	9.055249774	1.914051779	4.730932504	2.23E-06
Mthfd2l	70.19726334	9.053618699	2.161591751	4.188403612	2.81E-05
Zfp407	1147.372956	9.043378534	1.777764026	5.086939775	3.64E-07
2210416O15Rik	69.56655243	9.040230211	3.000431501	3.012976703	0.002586988
Acadvl	405.1987561	9.030658929	1.452081774	6.219111823	5.00E-10
NA	69.0934416	9.030421784	2.09153915	4.317596342	1.58E-05
Efnb2	68.99360657	9.02842227	3.173870788	2.844609272	0.004446593
Gm44093	68.62792863	9.020688427	3.217515387	2.803619359	0.005053252
Gm13312	68.37786438	9.015103691	1.943341468	4.638970474	3.50E-06
Gm43621	68.31389349	9.013465836	1.699522303	5.303529009	1.14E-07
Gstt3	67.73917438	9.002185467	2.030068437	4.434424624	9.23E-06
Bnip5	67.58375489	8.998687962	2.778098534	3.239153634	0.00119885
Nherf2	67.25698092	8.991649191	3.13578672	2.867430088	0.004138202
NA	268.38559	8.982410019	2.417463446	3.715634267	0.000202695
Ankrd13d	66.64498359	8.978478565	2.812543107	3.192299006	0.001411451
Teddm2	66.41100465	8.97342353	2.726712934	3.290930782	0.000998565
Oas1b	66.31965384	8.971339554	2.725036725	3.292190329	0.000994103
Hoxb5os	66.30864418	8.970816725	3.009659767	2.980674701	0.002876141
Gm31812	66.30061988	8.970631165	1.701849307	5.271107805	1.36E-07
9630010A21Rik	66.11112315	8.966797885	2.778568213	3.227128937	0.001250391
Tlr12	65.75477086	8.959038871	3.09047061	2.898923822	0.003744459
Stox2	65.03211423	8.943058639	2.858559058	3.128519809	0.001756892
A930004D18Rik	64.93212905	8.940404313	1.737536442	5.145448519	2.67E-07
Gm15559	64.76350249	8.937132442	3.253184022	2.74719548	0.00601073
NA	64.66964319	8.935019843	2.726635569	3.276939516	0.001049388
Dst	201.9880981	8.930939009	1.469194991	6.078797618	1.21E-09
Myom1	190.8620528	8.928985909	2.008496231	4.445607501	8.76E-06
Rnf123	64.27985306	8.926367957	2.746876695	3.249642757	0.001155501
Slc27a4	64.27407797	8.92609726	2.809305355	3.177332519	0.001486365
Gm15441	63.93839889	8.918561318	2.787474718	3.199512899	0.0013766
Gm49309	256.6529592	8.918280148	2.405286468	3.707782946	0.000209082
Intu	63.56011175	8.909948228	2.841128214	3.136059888	0.001712342
Dipk1b	63.37901977	8.905990262	2.697226719	3.301906435	0.000960301
Stxbp4	1812.619165	8.90237825	1.563322907	5.694523	1.24E-08
Rora	63.16647795	8.901143526	2.705330137	3.290224511	0.001001075
Mx2	63.11957466	8.900068244	2.754213609	3.231437175	0.001231694
Ndrg1	63.14282739	8.899950011	2.038396022	4.366153542	1.26E-05
Rnf40	249.5244765	8.899421808	1.979196295	4.496482654	6.91E-06
4933416I08Rik	63.06915696	8.898575797	1.422909507	6.253788982	4.01E-10
Zfp319	63.02046386	8.897901462	2.509149336	3.546182499	0.000390855
Npr1	62.95952877	8.896573476	2.144776319	4.148019259	3.35E-05
Fbxl21	62.91927153	8.895482996	3.119803561	2.851295866	0.004354143
Rhobtb1	62.88503893	8.89474341	2.787600523	3.190824272	0.001418675
NA	62.83091636	8.893369063	2.78166632	3.19713727	0.001387989
Fmn2	62.80989855	8.892886094	3.136090913	2.835659533	0.004573117
Rtl6	62.64505289	8.889071467	2.988807673	2.974119595	0.002938305
Ago4	62.47968325	8.885398404	2.940383207	3.02185048	0.002512346
Cfap96	62.27861096	8.880757105	2.802247626	3.16915501	0.001528828

NA	62.14823709	8.877555242	2.835561939	3.130792214	0.001743355
Rpgrip1	873.8705359	8.875742853	1.326273712	6.692240653	2.20E-11
Zdhhc1	61.9764848	8.873686411	2.747409059	3.229838085	0.001238603
Gm17619	61.89024965	8.871695055	2.828512575	3.136523109	0.00170964
Sbk1	61.78857769	8.86953619	2.289696009	3.873674128	0.000107207
Gm57142	61.7973653	8.869515201	2.703756179	3.280441953	0.001036446
E330037G11Rik	61.65801424	8.866213145	3.153538819	2.81151229	0.004930921
Trbv24	61.56601769	8.863971911	2.965940921	2.988586809	0.002802709
Phlda3	61.01336505	8.85095164	3.145887976	2.813498671	0.004900559
NA	60.95385021	8.849714648	2.057008278	4.302226074	1.69E-05
A430027C01Rik	60.80341866	8.846129	2.702154666	3.273731556	0.001061374
Gm16140	60.50589555	8.838457441	1.879283263	4.703100173	2.56E-06
Hk1os	60.46894032	8.838265062	2.12283065	4.163433886	3.13E-05
Stk19	60.16383434	8.830914577	2.936737915	3.00704892	0.002637973
Gm26533	60.1389221	8.829905772	2.869573727	3.07707925	0.002090397
Slamf6	483.6324087	8.82925601	1.760082846	5.016386605	5.27E-07
Nfkbie	336.1023455	8.828739841	1.582451318	5.579154153	2.42E-08
Stau2	854.0247719	8.828674729	1.288633994	6.851188755	7.32E-12
Ypel4	59.99809895	8.827172798	2.049485814	4.307018247	1.65E-05
Dpp7	530.3716404	8.826477588	1.552887731	5.683912246	1.32E-08
Baiap2l1	59.59844474	8.817300809	2.808828399	3.139138301	0.001694455
Spry1	59.00270491	8.802736809	2.721458508	3.234565871	0.001218278
NA	59.0063531	8.802730216	2.971266717	2.962618659	0.003050342
1500035N22Rik	58.96393327	8.801785439	2.704878926	3.25404045	0.001137761
NA	58.92925127	8.800875311	3.217060876	2.735688148	0.006224999
Zbtb5	393.3595439	8.795879064	1.668965266	5.270258912	1.36E-07
NA	58.69324841	8.795124487	2.716630691	3.23751201	0.001205769
Slc22a18	58.65286466	8.794087935	3.212910868	2.737109212	0.006198171
Gm36496	58.58114317	8.792454073	2.852152054	3.082743804	0.002051016
Gm28809	58.55957368	8.791819939	3.068803779	2.864901301	0.004171392
Gm47096	58.45709084	8.789346783	2.720236943	3.231096028	0.001233165
Gm32046	57.98617241	8.777747648	2.780735686	3.156627828	0.001596049
Gm37397	57.93625186	8.776395206	3.077847528	2.851471727	0.004351736
Tmem147os	57.88565111	8.775085681	2.821131354	3.110484618	0.001867806
Gm55006	57.87696014	8.774262724	2.025635223	4.331610462	1.48E-05
Trappc10	230.6701427	8.773915017	2.178916384	4.026733233	5.66E-05
Reck	57.66927833	8.769769755	3.012886444	2.910753498	0.003605584
Sqor	57.56014561	8.767026664	3.110441741	2.818579287	0.00482367
M1ap	57.53390034	8.766455079	2.798695273	3.132336401	0.00173421
NA	57.49088599	8.765237105	2.833643684	3.093274273	0.001979611
5930430L01Rik	57.28552638	8.760122022	1.779667383	4.922336671	8.55E-07
NA	57.2627686	8.759503335	3.141101568	2.788672427	0.005292457
Fam110a	189.7750551	8.757997084	1.181908053	7.410049419	1.26E-13
Pabir2	224.1941383	8.754724485	1.850269423	4.731594426	2.23E-06
Gm56824	56.96054956	8.751512802	3.073512154	2.847398144	0.00440782
A630035G10Rik	56.71671608	8.745776279	2.73395282	3.198949234	0.001379295
Gm26641	56.31544833	8.73539817	2.796702104	3.123463939	0.001787358
Hykk	56.07792768	8.729311578	2.990279986	2.919228841	0.003508985
Bbs7	160.90276	8.727461044	2.736225354	3.189598778	0.001424704
Trbj2-3	55.80395503	8.722200882	1.394931611	6.252780291	4.03E-10

Gm12971	55.61118276	8.716692703	1.813277399	4.807147934	1.53E-06
Gm36462	300.5628909	8.714161006	1.871947481	4.655131137	3.24E-06
Cplane2	55.43144114	8.712888299	1.476280058	5.901921016	3.59E-09
Pwwp2b	165.0544338	8.707242486	1.891266002	4.603922704	4.15E-06
Gbp6	55.1617366	8.705618864	3.087650735	2.819495989	0.004809913
NA	54.74971935	8.69474227	2.771143775	3.137600564	0.001703368
Stc2	54.6500881	8.69226149	2.883823469	3.014144791	0.002577048
2610037D02Rik	54.36525131	8.684867383	2.126849701	4.083441995	4.44E-05
Gm14161	54.30632283	8.682516246	1.941384151	4.472332919	7.74E-06
Rnf180	53.89847354	8.671856872	2.809417209	3.086710242	0.002023848
Kif3c	53.75145192	8.668073668	3.231078127	2.682718687	0.007302639
NA	53.37080607	8.657905711	2.05075786	4.221807889	2.42E-05
Bmx	53.3510777	8.657623596	2.414111631	3.586256529	0.000335459
Cracr2a	209.6723997	8.654066282	1.897641408	4.560432886	5.10E-06
Calr3	52.98934868	8.648074729	2.060758982	4.196548362	2.71E-05
Gm33044	52.90959795	8.645405463	2.830734414	3.054121015	0.00225721
Maob	52.90639409	8.645215157	3.096137832	2.792257847	0.005234163
Mmrn2	52.89863609	8.644793421	2.213258883	3.905911544	9.39E-05
Gm11655	52.75035332	8.640902243	1.649621534	5.238111932	1.62E-07
Gm56874	52.73091516	8.640695609	2.766291148	3.123566952	0.001786732
Plscr4	52.7053897	8.639910163	2.886988787	2.992706519	0.002765155
Zkscan7	212.872423	8.634563189	2.873427526	3.004969888	0.002656071
NA	52.22050894	8.626623882	2.712947561	3.179797503	0.00147378
Poll	154.8130226	8.623581296	1.975463947	4.365344815	1.27E-05
Abca3	52.10711501	8.623361077	3.137985913	2.748056019	0.005994977
Gm43604	51.87483462	8.616957266	2.932518556	2.938415257	0.003298948
Gm34854	51.64512444	8.610873118	2.37101674	3.631721773	0.000281537
Gm56927	51.52741046	8.607209884	2.803412171	3.070262009	0.002138711
Ncr3-ps	51.20305127	8.598251396	3.124825159	2.751594396	0.005930592
Pisd-ps1	50.90009761	8.589960777	1.863644941	4.609226032	4.04E-06
Plekhg5	50.79863482	8.58663638	3.222149576	2.664878267	0.00770162
Tmprss7	50.78323259	8.58622962	2.875996793	2.985479553	0.002831341
C130050O18Rik	50.70314314	8.584009976	2.767015847	3.102262672	0.001920475
Zfta	561.137192	8.583786978	1.180541377	7.271059824	3.57E-13
Gm16573	50.67515042	8.582691802	2.025365908	4.237600607	2.26E-05
Fam98c	50.51213306	8.578679921	3.000923072	2.858680384	0.004254072
Gm47322	50.50530351	8.578391903	2.739045216	3.131891308	0.001736842
NA	50.42492582	8.57677067	1.589838112	5.394744664	6.86E-08
Gng11	556.1352965	8.571465039	1.183329014	7.243518021	4.37E-13
Gm42572	49.96848209	8.562889433	2.323158945	3.685881868	0.000227912
Kbtbd6	49.8466407	8.559347507	2.965146137	2.886652837	0.003893636
Rnf125	148.657271	8.558807553	1.917899535	4.462594309	8.10E-06
Capn5	200.4057063	8.554549264	2.586606318	3.307248267	0.000942174
B130055M24Rik	95.6156334	8.554239401	2.56504526	3.334927276	0.000853217
NA	49.50378273	8.549485356	2.732368147	3.128965386	0.00175423
Col18a1	49.10836513	8.537918738	3.168696986	2.694457304	0.007050336
Xlr3b	884.7572655	8.535613553	1.103155884	7.73745005	1.01E-14
Camsap1	196.6841041	8.535045251	2.368757428	3.603174031	0.000314355
Atosb	48.82306595	8.529816585	2.339927943	3.645333016	0.000267046
Usp33	154.3345678	8.528984274	1.416228834	6.022320738	1.72E-09

Oxnad1	999.5492276	8.526511463	1.988764986	4.287339894	1.81E-05
Jade2	48.59259726	8.522722333	2.855965138	2.984182902	0.002843367
NA	48.59410385	8.522398556	3.106647479	2.743278281	0.006082911
Tnfsf11	48.56503649	8.521987744	2.76915725	3.077466165	0.002087685
NA	48.33701866	8.515126945	2.729560623	3.119596199	0.001810991
Robo1	48.29510915	8.513767418	2.922077787	2.91360054	0.003572868
Gprasp2	569.9565016	8.512404026	1.719945753	4.949228201	7.45E-07
Slc38a4	48.04995297	8.506582675	3.071942505	2.769121708	0.005620763
Medag	48.04614329	8.505929051	3.085602838	2.756650644	0.005839671
Trbj2-6	47.77760686	8.498159469	1.426996299	5.955277863	2.60E-09
Gm38313	47.68324344	8.495495609	2.804340084	3.029409899	0.00245032
Gm44731	47.58086054	8.491892322	3.063518284	2.771941126	0.005572311
Pygm	207.9707543	8.490394752	2.058882039	4.123788828	3.73E-05
Gm43857	47.35620393	8.485282931	2.811495551	3.018067351	0.002543924
Gpr84	47.35101967	8.485216887	2.942851768	2.883331393	0.003934932
Gm26387	47.31027383	8.483999654	2.846790849	2.98019774	0.002880624
NA	47.1499095	8.479196393	1.749262608	4.847297571	1.25E-06
Dkk1	47.08011942	8.476955468	2.855140326	2.969015354	0.002987557
NA	47.09630098	8.476715812	1.636309567	5.18038639	2.21E-07
Atf5	46.75422193	8.466990891	2.815286655	3.007505781	0.002634011
Crybg2	46.64612437	8.463733945	2.744797488	3.083554973	0.002045433
4933439C10Rik	183.7319276	8.462464107	1.90575182	4.440485911	8.98E-06
Trbj1-1	46.59327376	8.461746545	1.394148293	6.069473806	1.28E-09
Selenbp1	1061.031016	8.459183778	1.499433298	5.641587251	1.68E-08
Gm49708	46.31395316	8.453411229	2.739757034	3.085460179	0.002032374
Gm15927	45.98153346	8.44301116	2.802817765	3.012329687	0.002592509
Zfp846	369.121259	8.440668754	0.998564092	8.452806204	2.84E-17
E230029C05Rik	45.81479006	8.437319824	2.288252338	3.687233127	0.000226706
6430562O15Rik	45.74983013	8.435643274	2.848114069	2.961834769	0.003058118
Dip2a	45.52934858	8.428790208	2.322374134	3.629385156	0.000284097
Gm47603	45.53238953	8.428181971	2.446120837	3.44552969	0.000569941
Whamm	270.6433676	8.427986007	1.222725966	6.892784028	5.47E-12
Stard8	45.48094804	8.427519431	2.272248494	3.708889874	0.00020817
Gm29966	45.47647019	8.426895889	3.098673385	2.719517304	0.006537727
Gm5431	130.8729664	8.426518458	2.676643628	3.148165998	0.001642984
NA	45.37562118	8.424543628	1.714867843	4.912648904	8.99E-07
Kcnk5	45.29759857	8.421769674	2.263848989	3.720111066	0.000199135
Pou2af1	44.9775827	8.411085182	3.07208113	2.73791115	0.006183078
Eral1	920.6449975	8.40870247	1.905780516	4.412209275	1.02E-05
Serpinc1	44.86658363	8.407733024	2.801929916	3.000693549	0.002693655
B3galt4	44.82584926	8.40622155	3.146384656	2.671708157	0.007546625
Armc7	88.83914333	8.404251259	2.087853926	4.025306155	5.69E-05
Itgae	44.75364512	8.40403533	2.863704545	2.934672624	0.003338998
Castor2	237.3527275	8.397357812	2.074431462	4.048028564	5.17E-05
Gm42611	44.48157888	8.39530054	2.927134744	2.868095005	0.004129515
Gm38071	44.3077403	8.389654807	2.223911002	3.772477764	0.000161634
Lima1	44.22736942	8.387031996	2.808069349	2.986760992	0.002819501
Acot4	252.3139072	8.386686041	1.450323974	5.782629392	7.35E-09
Gm29438	44.1557288	8.384563623	3.057783064	2.742040049	0.00610589
Gm43080	44.0479313	8.380684799	2.882793195	2.907140482	0.003647494

Nrg4	727.9503764	8.379594422	1.423219579	5.887773428	3.91E-09
Arhgap28	233.0330196	8.377770497	2.142988758	3.909386116	9.25E-05
Ighv1-56	43.78088849	8.372345723	2.753286286	3.040855492	0.00235907
Tnfrsf4	43.73916232	8.370928342	2.734249967	3.061508071	0.002202251
Dnal4	43.52516143	8.364331709	1.861287462	4.493841966	6.99E-06
Gm31253	43.53362054	8.36413639	2.787253179	3.000852758	0.002692247
Sorbs3	455.076173	8.355962925	1.612443474	5.182174173	2.19E-07
Shisa2	42.93433279	8.343993489	2.27537803	3.667080098	0.000245336
Ets2	516.1144355	8.333861699	1.494787266	5.575282778	2.47E-08
Cbx7	84.78730271	8.331807084	2.37314961	3.510864654	0.000446652
Sgsm1	42.43117988	8.326716373	3.039104027	2.739858951	0.006146556
Ccdc8	165.8567981	8.324512419	1.789976701	4.650626132	3.31E-06
Zfp865	42.3495917	8.323652765	2.282767983	3.64629819	0.000266045
Acvr2b	42.31136031	8.322896465	3.03909398	2.738611086	0.006169931
Gm38111	42.30281115	8.32258244	3.096971288	2.687329545	0.007202585
Gm21057	42.28190021	8.322131108	2.786914787	2.986144803	0.002825188
A930037H05Rik	42.19112522	8.319028555	3.026436262	2.748786968	0.005981625
Akap17b	429.4513072	8.317939835	2.143712168	3.880157029	0.000104389
Nradd	42.12601568	8.316600397	3.074948789	2.704630537	0.00683804
NA	41.83671122	8.306450597	2.80726616	2.958910956	0.003087283
Tjp2	592.4116798	8.300984314	1.594146096	5.207166604	1.92E-07
C1qtnf6	119.816062	8.298926226	2.674991098	3.102412652	0.001919502
NA	41.56405686	8.296933622	2.932186909	2.829605983	0.004660536
Trappc11	698.1921779	8.296649672	1.677156685	4.946854247	7.54E-07
Wdr62	126.3829193	8.294049234	1.68290301	4.928417848	8.29E-07
Gm56915	41.45292208	8.293398984	2.799064376	2.962918272	0.003047375
Gm10827	41.15140397	8.282889409	2.990467642	2.769763931	0.005609693
Gm10941	41.10585405	8.281383155	2.850568705	2.905168761	0.003670551
Rgs3	41.0254367	8.279151799	1.787013034	4.632955464	3.60E-06
Tspyl4	40.99052073	8.277260157	3.039417743	2.723304547	0.006463245
NA	40.98570806	8.276882337	2.305159913	3.590589222	0.000329931
Gm19522	40.9315751	8.275167068	2.969195127	2.78700682	0.005319736
Mpl	2316.664948	8.269609128	1.779697437	4.646637658	3.37E-06
Gm16725	40.76252246	8.269365007	2.869834214	2.881478298	0.003958145
R3hdm2	120.7696561	8.268490872	2.015939286	4.101557488	4.10E-05
Serinc5	40.72640431	8.268219713	2.318774568	3.565771261	0.000362788
Pcp4l1	40.66411507	8.266115401	2.321222387	3.561104463	0.000369298
Trp53i11	40.6473087	8.26506731	2.792558334	2.959675796	0.003079629
NA	40.62544166	8.264236607	2.049102154	4.033101322	5.50E-05
Fam181b	40.60849886	8.264017499	2.227418062	3.710133109	0.00020715
Selp	40.51776692	8.260085651	3.01436267	2.740242816	0.006139381
Gm39326	40.49196569	8.259749909	2.858699643	2.88933814	0.003860537
Sstr2	120.6795247	8.259284422	1.936623481	4.264785852	2.00E-05
Fgfr1	40.4566162	8.258332881	1.902838864	4.340006417	1.42E-05
Cd36	2101.100383	8.258023968	1.209707484	6.826463485	8.70E-12
Zbtb12	40.42586772	8.257214105	2.976723955	2.773926716	0.005538415
Gm37660	40.39767138	8.256116665	2.988462595	2.762663545	0.005733183
Gnb4	657.1889744	8.25397742	1.609863849	5.127127629	2.94E-07
Zfp689	40.23175981	8.249949517	2.80480113	2.941367011	0.003267671
Dmc1	80.13718997	8.245754205	2.688648052	3.066877496	0.002163075

Nr6a1	39.96768184	8.240556233	2.28054203	3.613420022	0.000302185
Gm30906	39.89884915	8.238250653	2.802629635	2.939471755	0.003287722
NA	39.7997835	8.234763565	2.789545883	2.952008646	0.003157141
Lhpp	496.2672105	8.23380921	2.556951857	3.220165913	0.001281164
NA	39.78386929	8.233295536	1.617992872	5.088585788	3.61E-07
Scml4	39.52568474	8.224793156	3.078160771	2.671982969	0.007540448
NA	39.49877265	8.223987849	2.44636511	3.361717273	0.000774594
Hid1	39.50674516	8.223931555	3.057329889	2.689906505	0.007147204
Dock5	156.3913339	8.212718831	2.423818781	3.38833864	0.000703174
Gm9744	39.17191817	8.211921953	3.019496622	2.719632767	0.006535445
Bok	39.08179401	8.208369757	3.039120775	2.700902782	0.006915155
8430426J06Rik	39.02756271	8.206352301	2.89656248	2.833134917	0.004609393
Tmem198b	39.00074696	8.205629076	2.913629662	2.816291028	0.004858164
C430002N11Rik	38.91553039	8.202381433	2.95072952	2.779780856	0.005439559
NA	38.90085785	8.20190575	2.808447534	2.920441152	0.003495362
Zfp30	38.88015845	8.200954167	2.802172887	2.926641038	0.003426441
Fam53c	488.9896164	8.1960707	1.556535426	5.265585712	1.40E-07
Soga1	38.66453067	8.192922153	2.822151875	2.903076275	0.003695166
Gm47272	38.42938538	8.183696687	2.967672568	2.757614427	0.005822483
Gm56791	312.6473861	8.176509124	2.594588654	3.151370107	0.001625064
Gm49911	38.18095043	8.174956671	2.820831149	2.898066647	0.003754708
Arhgap25	1033.69124	8.16286058	1.063377088	7.676355523	1.64E-14
Tcp11l2	694.5402934	8.162455507	1.501057919	5.437801836	5.39E-08
NA	37.64040958	8.153786042	2.943397713	2.770195141	0.005602272
Dlc1	37.59670883	8.152867357	2.144337921	3.802044108	0.000143507
Klhl4	341.4547596	8.150191344	1.935488002	4.210923206	2.54E-05
Capn3	37.5029031	8.148707558	2.796966742	2.913408814	0.003575062
NA	37.25375804	8.139263698	3.043192139	2.674580942	0.007482271
Wfdc21	37.21917395	8.138166704	2.78641791	2.920655467	0.003492959
Dyrk4	291.6422749	8.134690167	1.291255593	6.299829568	2.98E-10
Gm34102	37.13293163	8.134629374	2.992913977	2.717962974	0.006568519
Gm57080	37.13528346	8.134515902	2.799807195	2.905384313	0.003668024
Sgsm3	165.9994098	8.13180788	2.39191368	3.399707919	0.000674579
Hemgn	463.2011024	8.129547618	2.421143349	3.35773081	0.000785851
Gm9256	36.98474587	8.129306595	2.126397086	3.823042577	0.000131815
Palld	686.0537375	8.119079157	1.128157256	7.196761898	6.17E-13
Eml2	36.52778978	8.111136968	2.92373996	2.774233372	0.005533197
6030443J06Rik	36.31349417	8.102709244	2.16014474	3.751002928	0.000176129
Abhd4	204.7212934	8.098026638	1.510881297	5.359803349	8.33E-08
Gm15952	36.19295718	8.097629854	2.321017465	3.488827626	0.000485144
Slc66a1	36.05618223	8.092858106	1.885641314	4.291833258	1.77E-05
Cipc	861.4948556	8.090011216	1.026753464	7.879214922	3.29E-15
NA	35.97109539	8.088961564	2.38308887	3.394318049	0.000687997
Mdga1	35.9484744	8.087912467	2.821684693	2.866341689	0.004152458
Gm13594	35.90665383	8.08696059	1.710816591	4.726959413	2.28E-06
Naip6	511.3353494	8.086488477	1.558092563	5.189992346	2.10E-07
Gm49312	35.8642979	8.084367375	2.9240146	2.764817719	0.005695461
Ranbp3l	35.86026004	8.08433942	2.786769997	2.90097117	0.003720081
Cimip4	35.7532747	8.080126421	2.783134504	2.903246829	0.003693154
Nlrc5	70.89049568	8.078431035	2.025301485	3.988754807	6.64E-05

NA	70.81117489	8.077242175	2.063963953	3.913460874	9.10E-05
Zfp109	105.2365237	8.070204872	2.021894938	3.991406636	6.57E-05
Kif17	570.4303524	8.068126286	1.125927333	7.165761104	7.74E-13
Cyp7b1	35.36758431	8.064508797	2.77722996	2.903795837	0.003686685
Mdrl	35.36319661	8.064291774	2.785635513	2.894955832	0.003792119
Bcl6b	35.34069679	8.063181838	2.558511499	3.151512839	0.00162427
Gm50205	35.20405907	8.057802108	2.814539689	2.86292005	0.004197565
Slc2a3	808.4155168	8.055319079	1.672046931	4.817639344	1.45E-06
Wdr11	1340.588594	8.049562588	1.194517123	6.738758645	1.60E-11
D330050I16Rik	68.99954941	8.034294927	2.379931635	3.375851141	0.000735877
Fah	34.48215203	8.027960111	2.988110451	2.686634327	0.007217592
Ehd3	566.7845946	8.025974652	2.530421949	3.171793011	0.001515009
NA	34.30987417	8.021056923	2.308085966	3.475198515	0.000510475
Gm47586	149.0269507	8.013302961	2.018599093	3.969734748	7.20E-05
Ift140	279.0274511	8.011778007	2.612901878	3.066237609	0.00216771
Qprt	65.86995209	8.010845018	1.892264045	4.233471031	2.30E-05
Tmem69	464.2850033	8.00519083	1.049120243	7.630384489	2.34E-14
Prkn	33.81393113	7.999086915	2.946699366	2.714592133	0.006635744
Gm43489	33.67358284	7.993783818	2.945587418	2.713816527	0.006651299
1700084E18Rik	33.6717613	7.993586865	2.787005072	2.868163731	0.004128618
Vps13d	478.631805	7.993074082	1.307104696	6.115098591	9.65E-10
Gm10277	33.64492424	7.991247844	1.945665188	4.107206056	4.00E-05
Gm17151	33.60961687	7.990977254	2.787350729	2.866871819	0.004145509
Sptb	33.44956304	7.984012213	2.820709235	2.830498129	0.004647558
Hoxb3o	33.44833087	7.983959932	2.817060522	2.834145688	0.004594838
Gng3	33.44456018	7.983439665	2.812420411	2.838636654	0.004530671
Gm9378	33.43057049	7.983141597	2.412140201	3.309567825	0.000934401
Fkbpl	33.31626901	7.978399719	2.933727284	2.719543756	0.006537205
Zfp831	96.33739836	7.976141336	2.514656862	3.171860725	0.001514656
Plcg1	764.8075054	7.975997416	1.585679874	5.030017438	4.90E-07
Lfng	33.222509	7.974109743	2.836748648	2.811003276	0.004938728
Supt7l	1405.510625	7.969829416	0.946720527	8.418354931	3.82E-17
Nelfb	940.5846932	7.960179667	0.711292555	11.19114718	4.51E-29
Igsf23	32.77942396	7.954898436	2.778958984	2.862546185	0.00420252
Csf3r	433.6908725	7.949433007	1.734825776	4.582265906	4.60E-06
Snca	94.31712498	7.944794786	2.500679904	3.177053878	0.001487794
Trbv3	32.48552777	7.942106433	2.529516968	3.139771954	0.001690794
Prdm16	471.6840074	7.933596855	1.52161017	5.213948362	1.85E-07
Gm42890	32.06021529	7.922965092	2.849571615	2.780405676	0.005429103
Gm30938	32.05781753	7.922246447	2.851151059	2.778613368	0.005459146
Dusp1	126.1957126	7.917168051	1.927128041	4.108272975	3.99E-05
Sulf2	31.90726322	7.91580823	2.829951867	2.797152956	0.005155512
H1f1	127.8086577	7.910754613	2.390417537	3.309361018	0.000935092
Evc2	31.59529236	7.90189231	2.856056644	2.766714143	0.005662438
Ptpn21	31.56168137	7.90029016	2.808748291	2.812744092	0.004912073
Wincr1	31.51900797	7.898181122	2.868868617	2.753064771	0.005904021
Zfp987	335.3512877	7.890729246	1.221970842	6.45737932	1.07E-10
Gm23849	31.25144217	7.885897348	2.828057839	2.788449812	0.005296095
NA	31.23978008	7.884966883	2.28611176	3.449073235	0.000562514
Pax5	91.85231684	7.878812766	2.080880986	3.786287068	0.000152915

Exoc6b	251.6342728	7.877802291	1.920029056	4.102959935	4.08E-05
Plekha8	375.1261049	7.87699541	1.410178463	5.585814573	2.33E-08
NA	60.32792337	7.876039153	1.386725836	5.679593577	1.35E-08
Tesk2	188.8009636	7.872337279	2.444029895	3.221047867	0.001277228
Asb13	30.90927626	7.87034399	2.487857265	3.163503028	0.001558827
Triqk	125.0913913	7.868452199	2.748191064	2.863138703	0.004194669
Nudt18	30.84501231	7.867684877	1.962945827	4.00810087	6.12E-05
Agtrap	491.0581598	7.857805569	1.201245946	6.541379467	6.10E-11
Tmed8	1305.675618	7.854356559	1.732854341	4.532612102	5.83E-06
Gas8	320.1284082	7.853644095	2.02438021	3.879530168	0.000104658
Gm20531	30.52758292	7.852243549	2.860083145	2.745459887	0.006042615
Snord91a	30.44932649	7.848499685	2.800116804	2.802918676	0.005064244
Zdhhc9	474.2482459	7.838799653	1.384074859	5.663566245	1.48E-08
Gm45292	30.22783716	7.83779358	2.886814673	2.71503178	0.006626941
NA	30.11440046	7.832550506	2.884334178	2.715548901	0.0066166
Pdgfrb	155.641881	7.82894774	2.579409521	3.035170521	0.002403997
Acer2	486.3222706	7.816324233	1.68040102	4.651463634	3.30E-06
Nynrin	228.6953528	7.814845808	2.335189113	3.346558001	0.000818216
Gsta4	133.316907	7.814499609	2.392268625	3.266564435	0.00108861
Zfyve26	408.1429764	7.806673625	1.582418854	4.933380062	8.08E-07
2900005J15Rik	29.55187764	7.805441525	2.554959897	3.05501528	0.002250491
Pira1	29.42104676	7.798843939	2.916709128	2.673850424	0.007498589
NA	29.34220807	7.794475351	2.294642641	3.396814481	0.000681752
Spag1	29.09306244	7.782693725	2.12579387	3.66107638	0.000251158
Nalf1	113.2671624	7.778569109	1.725925086	4.506898459	6.58E-06
Zfp719	2886.895803	7.773478389	1.37712913	5.644698251	1.65E-08
Nlrx1	28.87432307	7.771893271	2.79750002	2.778156645	0.005466826
Tcn2	540.9636808	7.769677538	1.129728645	6.87747237	6.09E-12
Itpkb	234.6578317	7.764697024	2.386614737	3.253435464	0.001140186
4930506C21Rik	28.41348083	7.74872721	2.906532934	2.665969176	0.007676673
Nudt12	843.2028163	7.740234999	1.795256708	4.311492036	1.62E-05
Efcab12	28.20533222	7.738193348	2.891632287	2.676064098	0.00744924
Cyp4f13	28.09476769	7.731793655	2.200826257	3.513132229	0.000442857
Mettl17	698.8824974	7.731382569	1.129250752	6.846471036	7.57E-12
Hfm1	113.6211453	7.728325017	2.789865134	2.770142873	0.005603171
Nepro	460.8373616	7.726549377	2.519214001	3.067047648	0.002161844
Zfp7	146.4857659	7.724007416	2.339869709	3.301041671	0.000963266
Atg2a	146.1504184	7.718808247	2.3166504	3.331883069	0.000862605
Dctn1	85.87858951	7.713557251	1.561286091	4.940514934	7.79E-07
Gm43292	27.65363147	7.709510103	2.852682693	2.702547368	0.006881038
Ccdc142os	27.53245401	7.7032646	2.886590435	2.668637887	0.007615951
1500026H17Rik	27.36444955	7.694367836	2.816808226	2.731590942	0.006302934
Actn2	27.28642164	7.690957644	2.043344582	3.763906349	0.00016728
Gzf1	181.7784779	7.687559709	2.015648009	3.813939574	0.000136769
Def8	219.0085855	7.68369454	1.75490601	4.378408015	1.20E-05
NA	27.1096096	7.680879105	2.820742489	2.722999045	0.006469224
Phc3	332.5316432	7.680856717	2.020724256	3.801041479	0.000144089
Snord8	26.96712302	7.673360253	2.816492184	2.724438682	0.006441089
Tef	440.2197158	7.66340547	2.18377856	3.509241098	0.000449387
Oas1c	243.9871573	7.662379913	1.952564396	3.924264894	8.70E-05

B230208H11Rik	26.68210091	7.658459124	2.363471406	3.240343464	0.001193858
Mboat2	103.8243682	7.658127649	1.680020418	4.558353913	5.16E-06
Aph1a	26.66944623	7.657021398	2.187940438	3.499648008	0.000465873
Gas2	312.5147983	7.65607568	2.021888191	3.786596961	0.000152725
Xlr3c	26.58021265	7.65200669	1.577062275	4.852063745	1.22E-06
Phf8	435.4011753	7.644394382	2.492818558	3.066566701	0.002165325
Gm22334	26.41633856	7.643502473	2.860716419	2.671884015	0.007542671
Ldb2	104.7698735	7.642638931	2.004916331	3.811949063	0.000137875
NA	26.36607876	7.640759754	2.814436397	2.714845417	0.006630671
B3gat2	111.6240432	7.619325247	1.850657631	4.11709066	3.84E-05
Ubtd1	25.97545966	7.619274455	2.822897255	2.699097334	0.006952784
Cd5	104.2403469	7.616292427	2.372827506	3.20979608	0.001328292
Acot1	355.7581106	7.611552242	1.716152651	4.435241957	9.20E-06
Gbp2	643.4845551	7.611123801	1.23076612	6.18405372	6.25E-10
Gata6	25.80976607	7.610078746	2.760511838	2.75676367	0.005837653
Rab23	150.5061372	7.600744655	1.297088931	5.859848523	4.63E-09
4931422A03Rik	25.62158472	7.59990756	1.757451597	4.324390825	1.53E-05
Nrk	352.8333903	7.598399993	1.733814194	4.382476518	1.17E-05
NA	477.4249017	7.592184064	2.039275404	3.722981236	0.000196884
Ggact	73.99103671	7.591807133	2.47189319	3.071252093	0.002131631
NA	25.48755745	7.591306933	1.889312466	4.01802617	5.87E-05
Gm45345	25.36875019	7.585197981	2.845295195	2.665873824	0.007678851
Sipa1l1	207.0356465	7.583311951	2.388008002	3.175580629	0.00149537
Rel	748.6770215	7.57591776	2.041464208	3.711021595	0.000206424
Slc41a1	448.3863332	7.573314732	1.655563254	4.574464136	4.77E-06
Ppp4r1	1483.020039	7.568052162	1.375493549	5.502062999	3.75E-08
Psd3	478.2415369	7.565498766	1.149764506	6.58004202	4.70E-11
Fbxo25	195.9504671	7.563883906	1.969341803	3.840818234	0.000122625
Ccser2	438.4744729	7.563159831	2.045471344	3.697514441	0.000217721
Zswim4	97.08566777	7.563067781	1.661915045	4.550814918	5.34E-06
Coro2b	101.6432768	7.562711596	1.495477831	5.057053632	4.26E-07
Ptpn9	257.1322165	7.559332372	1.90588017	3.966320912	7.30E-05
Nfib	339.2367425	7.558239628	1.534211399	4.926465567	8.37E-07
Zfp455	720.8183522	7.547037744	1.205346977	6.261298937	3.82E-10
Cnksr3	304.563236	7.544013398	2.809227517	2.685440517	0.007243427
Ccr7	201.3590421	7.542578141	2.415552215	3.122506769	0.00179318
Hnf4a	132.8789038	7.528451711	1.832845888	4.107520311	4.00E-05
Slamf1	111.0211248	7.525437187	2.772125677	2.714681102	0.006633962
Gm49700	24.14840667	7.513968832	2.363654402	3.178962553	0.001478032
Tcaim	292.1839114	7.506853748	1.569076631	4.784249285	1.72E-06
Neto2	587.4156345	7.492920091	1.858432881	4.031848644	5.53E-05
Zfp438	239.9009053	7.490771583	2.448643605	3.059151428	0.002219649
Lrrc8b	508.9360596	7.488552578	1.564149835	4.787618429	1.69E-06
Gcfc2	230.4443253	7.487710869	2.186149367	3.425068287	0.000614645
4933439K11Rik	180.1022063	7.480724132	2.556554585	2.926095995	0.00343245
Selenbp2	23.44230779	7.47071572	1.876236856	3.981755126	6.84E-05
Gm12009	23.43215211	7.469840853	1.71139266	4.364773222	1.27E-05
Fkbp9	68.96518622	7.456119078	1.996658072	3.73429942	0.000188238
Far2	310.6058839	7.4521287	1.353025526	5.507751742	3.63E-08
Grina	103.7424412	7.451914914	2.382854822	3.127305468	0.001764165

Ess2	153.1975667	7.44091485	2.076946656	3.582622032	0.000340163
H2ac6	134.8097191	7.43840755	1.450751898	5.127277492	2.94E-07
Gm2011	95.45053095	7.43032339	1.655847243	4.487324192	7.21E-06
Gm57232	22.76014572	7.427904185	1.556665877	4.771675345	1.83E-06
Syne4	101.8785078	7.424537041	2.396671695	3.097853184	0.00194928
Wdr7	176.0502605	7.422521948	2.137574901	3.47240321	0.000515821
Kif21b	511.33408	7.418213305	1.150319861	6.448826589	1.13E-10
Card11	89.68406648	7.398834663	2.36209309	3.13232137	0.001734299
Slc35a5	1082.131476	7.398677186	1.08274838	6.833237826	8.30E-12
Zfp280b	320.5676769	7.39446083	1.573893064	4.698197737	2.62E-06
Fsbp	238.1993224	7.394018969	1.379321806	5.360619207	8.29E-08
Flcn	817.2865539	7.382555901	1.619871131	4.557495815	5.18E-06
B3gnt7	333.9185075	7.381084619	1.976999919	3.733477452	0.000188854
Syt11	208.2434508	7.379883986	1.356711192	5.439539401	5.34E-08
Snord98	22.00640669	7.378494341	2.169135712	3.401582621	0.000669969
E430018J23Rik	43.55027035	7.371859803	2.089212666	3.528534898	0.000417867
Erg	1028.190213	7.371067659	1.067235143	6.906695032	4.96E-12
Gm37219	187.0111392	7.369743247	1.278232401	5.765573802	8.14E-09
Gm56655	21.68581627	7.357223064	1.90778475	3.856421991	0.000115059
4930592A05Rik	21.48269844	7.344203718	2.475362645	2.966920314	0.003007989
Hoxb4	86.68155361	7.33880285	2.680610553	2.737735566	0.00618638
Zfp597	263.4394231	7.337091166	2.4514952	2.992904562	0.002763361
Zfp963	21.26092792	7.329448818	2.136231505	3.431018034	0.000601321
Tmc4	234.6649579	7.329327924	1.353262401	5.416043422	6.09E-08
Aplp2	782.8672523	7.327635212	1.069836902	6.849301231	7.42E-12
Aldh6a1	604.6506071	7.32641711	0.883646928	8.291113651	1.12E-16
Ccdc91	787.2611501	7.325886664	0.679736342	10.77754154	4.39E-27
Osbpl3	111.7339241	7.317923921	2.182590402	3.352861771	0.000799806
Entrep3	60.45955901	7.311315889	2.722982224	2.68503989	0.007252115
NA	71.4407956	7.305333629	1.787924839	4.08592882	4.39E-05
Hip1r	20.79224659	7.298104723	2.25128678	3.241748136	0.00118799
Fxyd2	85.34297085	7.292601702	1.552522897	4.697258711	2.64E-06
Rbpj-ps3	20.67766228	7.290071183	2.01833822	3.611917523	0.000303941
Hs1bp3	81.99399649	7.287604065	2.003621084	3.637216698	0.0002756
Dusp5	166.423533	7.284788583	1.777747705	4.097762895	4.17E-05
Myo5c	208.4641765	7.277915604	1.830847895	3.975161249	7.03E-05
Snx29	40.84682047	7.273638979	2.403423312	3.02636616	0.002475124
Airn	166.1672797	7.271053499	2.102822817	3.457758515	0.000544689
Il4ra	266.8668706	7.270961625	1.52233214	4.776199249	1.79E-06
Tbc1d12	167.0262948	7.269578396	2.545859556	2.855451463	0.004297569
Ppp1r13b	283.9205892	7.268506934	2.002296332	3.630085526	0.000283327
Gm12444	20.22392327	7.257741653	1.799073869	4.034154337	5.48E-05
Ing4	509.2129901	7.24806123	1.298682777	5.581086743	2.39E-08
Nav1	564.612667	7.246780554	1.013918944	7.147297716	8.85E-13
Rhbdd2	251.5163156	7.242803214	2.091023797	3.463759344	0.000532683
Nit1	292.60119	7.239917187	0.972327434	7.445966177	9.62E-14
Ipcef1	692.3169371	7.235721506	1.093118939	6.619335963	3.61E-11
Mink1	57.62672099	7.228926513	2.452112754	2.948040012	0.003197957
Rnu12	19.76429002	7.225462041	1.841418957	3.923855575	8.71E-05
Tmem161a	212.3573534	7.205912441	0.904153172	7.969791694	1.59E-15

H2bc6	332.7968692	7.205210146	2.368614524	3.041951349	0.002350499
Dnajb2	56.27550482	7.204769099	2.656639396	2.711986095	0.00668814
Gm16279	19.41801099	7.199654812	2.224070955	3.237151582	0.001207293
Peli2	128.9455261	7.192663029	2.162128046	3.326659141	0.000878938
Dlg2	326.8759474	7.191319272	1.218387514	5.902325156	3.58E-09
Dennd1c	506.0937089	7.188855333	1.672703761	4.297745661	1.73E-05
Als2	276.7091441	7.188569611	2.010397134	3.575696308	0.000349297
Emid1	194.5645124	7.188355409	2.47837436	2.900431639	0.003726491
Tead2	19.22415177	7.183788126	2.389806887	3.006011978	0.002646986
Trbj2-2	19.16467861	7.180549129	1.732431069	4.14478201	3.40E-05
Gm37407	166.013148	7.180267952	2.110312489	3.402466691	0.000667805
Slc22a21	152.5523083	7.176963303	1.481608296	4.84403558	1.27E-06
Cpa3	927.9434439	7.174341532	2.199795821	3.261367015	0.001108764
Scaf1	55.07058305	7.170198604	2.584071657	2.774767713	0.005524115
Itpk1	274.6510904	7.166105543	1.510767361	4.743354753	2.10E-06
Flot2	56.3483376	7.16467615	2.004932071	3.573525634	0.000352207
Obsl1	162.8853106	7.160659139	1.31483967	5.446032168	5.15E-08
Rnf122	317.9544896	7.158397725	1.562193476	4.582273473	4.60E-06
Trbj1-2	18.85726477	7.15793742	1.601239503	4.470247833	7.81E-06
F2rl3	240.8689244	7.153247114	1.853980995	3.858317389	0.00011417
NA	75.81402656	7.152548664	2.43182765	2.941223513	0.003269185
Zfp457	18.79516556	7.152385438	2.601517985	2.749312316	0.005972045
Wdtc1	54.25554855	7.148943946	2.593497306	2.756487901	0.005842577
Cd200r4	187.7059964	7.147616523	1.989718009	3.592276137	0.000327802
Raf1	348.0293753	7.141438355	1.54126897	4.633479617	3.60E-06
4933404O12Rik	200.0595174	7.1399448	2.089314183	3.417362911	0.000632309
F2r	426.5941734	7.118500806	1.543883114	4.610777035	4.01E-06
Gtf2ird2	273.8831364	7.116793663	2.070030376	3.438014122	0.000585997
Gm56507	108.0837682	7.11077597	2.482080294	2.864845262	0.004172131
Zfp493	608.317331	7.11031366	1.25556596	5.663034749	1.49E-08
Gm22956	18.21064868	7.106079817	2.019910556	3.518017071	0.000434784
Dnajb9	188.4730378	7.100802735	1.811919701	3.918938974	8.89E-05
Gm43609	18.0842988	7.095825206	2.048382016	3.464112236	0.000531985
Sec16a	506.5910876	7.093191227	2.094763779	3.386153273	0.000708798
Stn1	932.0164148	7.090310793	0.946234719	7.493183929	6.72E-14
Naa80	159.4558396	7.088540303	1.539581171	4.604200439	4.14E-06
Slc29a3	446.064133	7.085367973	1.8342383	3.862839399	0.000112077
Gm55238	17.91779605	7.084329773	2.107853389	3.3609215	0.000776829
Zfp595	260.5136896	7.073387855	1.507621679	4.691752548	2.71E-06
Pdpr	671.7663145	7.067437784	1.953346845	3.618117183	0.000296754
Vcl	144.3833104	7.059455651	2.468822457	2.859442416	0.004243865
Kifbp	244.1793247	7.054770595	1.900091983	3.712857408	0.000204932
Pdgfb	17.44254869	7.044913243	1.91229206	3.684015318	0.000229588
Gm7334	17.44181184	7.0448944	2.227677292	3.162439383	0.001564533
Awat2	142.5963591	7.040022587	2.55635365	2.7539314	0.005888411
Ankrd16	509.888248	7.039442654	1.886988235	3.730517512	0.000191087
Trerf1	152.761824	7.03620697	1.061722707	6.627160676	3.42E-11
Hacd4	3364.61654	7.030558533	1.567800789	4.4843443	7.31E-06
Galnt16	17.2388993	7.027778788	1.561221869	4.501460637	6.75E-06
Prkch	600.111422	7.027515617	0.887914496	7.914631024	2.48E-15

Nphp3	278.7592196	7.016813376	1.463958728	4.793040434	1.64E-06
Vipr2	429.7269559	7.014338047	1.660441611	4.224381033	2.40E-05
Csrnp1	77.16042655	7.007139286	2.60552329	2.689340492	0.007159335
Slc18b1	16.92549207	7.001924252	2.626147488	2.666234202	0.007670624
Map2k3os	211.881769	6.999891744	1.277338487	5.480060152	4.25E-08
Cnr2	130.7401518	6.992873319	2.158397293	3.239845297	0.001195946
Hemk1	235.8573616	6.983649416	2.481039632	2.814807682	0.004880643
Aff3	221.1743091	6.979636044	2.217472028	3.147564414	0.001646368
Pear1	213.5507793	6.978585231	1.849732492	3.772753769	0.000161456
Slc26a6	67.45130739	6.978076366	2.585249602	2.699188644	0.006950876
Myo18a	396.0435155	6.970579615	0.950926426	7.330303819	2.30E-13
Tle6	124.7900233	6.949912225	2.57607289	2.697870954	0.006978449
Gm55563	16.23278361	6.940770388	2.552965515	2.718709026	0.006553723
Tbcd	695.6957751	6.931752714	0.722993652	9.587570651	9.02E-22
Tnfsf9	195.9469612	6.913928034	1.931675946	3.579238044	0.000344597
Pan2	418.0985073	6.908091678	1.645581024	4.197965083	2.69E-05
Atp1b1	447.8533033	6.906943419	1.793468695	3.85116475	0.000117557
Snx19	464.7666198	6.904544654	1.003804609	6.878375125	6.05E-12
Iqce	184.4743047	6.899025178	2.430297104	2.838757931	0.00452895
Cdk5	409.4325381	6.88881314	0.77936339	8.839025835	9.66E-19
Mical2	306.0324993	6.888432172	1.927958894	3.57291444	0.00035303
Zswim8	65.9516454	6.885523971	1.691319073	4.071096981	4.68E-05
Armcx4	273.0838816	6.884778941	1.099825287	6.259884201	3.85E-10
Gm5530	15.56204396	6.880548953	1.795208335	3.832730061	0.000126729
Zfp72	707.434864	6.876624364	0.7228345	9.513414706	1.85E-21
NA	15.40027346	6.865527574	2.070222425	3.316323644	0.000912101
Eps15l1	864.7368022	6.859545666	1.333467507	5.144141594	2.69E-07
Nhs	188.5834007	6.858742461	2.008032235	3.415653564	0.000636291
Rwdd2b	378.9627247	6.847987072	0.715623993	9.569253043	1.08E-21
Tspyl3	344.1468251	6.845720054	1.484904835	4.610207937	4.02E-06
Bnip1	750.3046374	6.838303546	1.578312816	4.332666806	1.47E-05
Rpusd4	811.2848507	6.809722928	1.260208243	5.403648935	6.53E-08
Epm2aip1	622.8319209	6.809642398	1.035024434	6.579209315	4.73E-11
E2f1	301.8564948	6.806052279	0.938365326	7.253094389	4.07E-13
Spata9	14.76215846	6.802396833	2.135210783	3.18581982	0.001443445
Samd1	333.9567918	6.78349174	1.479610439	4.584647121	4.55E-06
Jam2	57.68619185	6.780763548	1.949318746	3.478529903	0.000504172
Dock9	326.4353093	6.780312192	1.566719977	4.327711583	1.51E-05
Golga1	1354.687307	6.773842316	1.334464978	5.076073503	3.85E-07
Bend5	110.9251872	6.767557188	2.357510734	2.87063685	0.004096458
Pdgfa	14.38056184	6.766776709	2.190509509	3.089133684	0.002007411
Rad9a	387.0926509	6.761290407	1.577438424	4.286246806	1.82E-05
NA	14.28429794	6.757548424	2.043499837	3.306850483	0.000943512
Tmub2	847.5112898	6.752593651	0.833858184	8.098012084	5.59E-16
Cav2	449.1312436	6.75019686	0.99709558	6.769859378	1.29E-11
Lrrc47	102.0469547	6.744618615	1.266342633	5.3260614	1.00E-07
Ap5s1	239.7623452	6.74414698	1.675888637	4.024221437	5.72E-05
Lrrc27	143.1974757	6.7382017	2.353674257	2.862843777	0.004198575
Jade3	374.3497224	6.737326509	1.210306309	5.566629255	2.60E-08
Matcap2	114.1422522	6.736415786	1.799367335	3.743769076	0.00018128

Cryzl2	521.590637	6.730763984	1.63301841	4.121670609	3.76E-05
Zfp532	13.98841065	6.724424567	2.289826053	2.936653008	0.003317751
Mcm9	705.870641	6.722956582	1.186220409	5.667544187	1.45E-08
Carm1	26.99033951	6.720459967	1.787317525	3.760081728	0.000169858
Iqgap3	144.3848326	6.712420716	1.790170634	3.749598273	0.000177118
Rilpl1	27.59263475	6.712314705	1.947558493	3.446527911	0.00056784
Abca5	13.7993497	6.706276847	2.039371614	3.288403545	0.001007573
Trim68	169.4447667	6.702728362	2.036642363	3.291067927	0.000998078
Snx13	436.1289417	6.699232119	2.185303807	3.065583879	0.002172454
Arhgap23	105.135403	6.695342749	2.460378723	2.721265099	0.006503259
Zfyve27	629.4694066	6.69242155	0.503994035	13.27877135	3.07E-40
Yipf2	54.9934347	6.687564189	2.40138742	2.784875166	0.005354833
Dclre1b	618.5463062	6.687223326	0.70951169	9.425106614	4.30E-21
NA	13.59030218	6.684463127	1.872294857	3.570197878	0.000356712
Osbp	214.9445542	6.68382601	2.073802395	3.222981141	0.001268639
Mapk3	239.8447888	6.679683658	1.868522897	3.574847098	0.000350433
Abca2	160.4533405	6.678897847	2.164820166	3.085197539	0.00203417
Lrrc45	190.5397421	6.673593256	2.486640179	2.683779226	0.007279516
Ppm1d	194.4502403	6.673352807	1.640985757	4.066673206	4.77E-05
Lgals4	224.9928475	6.673256721	1.620026936	4.119225781	3.80E-05
Arhgap27	160.5869198	6.668100413	1.55875746	4.277830633	1.89E-05
Eif2ak3	647.8140253	6.650166335	1.544443178	4.305866625	1.66E-05
Gm17201	38.6542459	6.64686438	2.390260277	2.780811966	0.005422313
Cln8	267.5723951	6.646278632	1.776464149	3.741296236	0.000183074
Hdac6	68.91979895	6.639743387	2.453923889	2.705765821	0.006814708
Nacc1	351.289112	6.63713683	1.155489502	5.744004441	9.25E-09
Bltp1	987.7797719	6.622321416	0.806577555	8.210396348	2.20E-16
Hyi	268.6090953	6.620863197	2.159612822	3.065763978	0.002171146
Tacc2	122.6299541	6.614683443	1.960262512	3.374386543	0.000739804
Edc4	386.1059185	6.613966445	1.32359423	4.996974372	5.82E-07
Xkr8	326.6700633	6.613105372	1.608558855	4.111198887	3.94E-05
Dgke	554.199816	6.612500092	1.429916297	4.62439662	3.76E-06
NA	37.49956531	6.601246405	2.36454625	2.791760324	0.005242217
Tctn3	178.1965987	6.601161673	1.829799158	3.607588102	0.000309057
Mcoln1	266.4194606	6.589955966	1.618101481	4.072646891	4.65E-05
Spty2d1	531.0793068	6.578396613	1.938987659	3.392696484	0.000692083
Ptgir	12.57606106	6.572076812	1.841524657	3.568823685	0.000358588
Wdr24	299.6968508	6.568455415	2.121691613	3.095857747	0.001962445
Dop1b	131.059096	6.563213097	1.236208569	5.309147065	1.10E-07
Padi4	56.3044394	6.561085082	2.431855313	2.6979751	0.006976266
Cd151	277.0278053	6.554537435	1.046330983	6.264305979	3.74E-10
Rnf113a1	48.47742404	6.546368714	1.744750387	3.752037405	0.000175403
Ern1	445.7453529	6.546352838	1.187552435	5.51247477	3.54E-08
Cc2d1b	255.8087271	6.541888143	1.060407469	6.169221107	6.86E-10
Cenpp	604.8566022	6.538681757	0.718229004	9.103895438	8.71E-20
Trim39	349.7294064	6.538598526	2.171777981	3.010712229	0.002606357
Specc1	1420.282911	6.533923408	1.526251546	4.281026561	1.86E-05
Mark4	82.00298306	6.532955679	2.034681596	3.210800006	0.00132366
Pgghg	206.849151	6.528796589	1.696264825	3.848925293	0.000118637
Zfp599	794.039314	6.524573453	0.787461164	8.285581243	1.18E-16

Szt2	256.6989985	6.524217919	1.826920526	3.571155847	0.000355409
Pou5f2	113.2311346	6.523694631	2.418174486	2.697776636	0.006980426
Nupl2	149.7575553	6.517462364	2.422557842	2.690322703	0.007138295
Dhx34	12.08922016	6.517163942	2.08486383	3.125942255	0.001772363
Sergef	349.6927275	6.513743601	1.973321177	3.300903916	0.000963739
Gm19331	319.4716583	6.510733027	2.014991021	3.231147415	0.001232943
Gmeb2	249.2398846	6.50997166	2.423768463	2.685888426	0.007233724
Epb41l5	296.8612576	6.505106457	1.67814154	3.876375324	0.000106024
Gm12216	93.4339259	6.499400526	1.284556289	5.059646339	4.20E-07
Fstl1	1426.130635	6.494781404	1.344466145	4.830751172	1.36E-06
Omd	109.6244604	6.494113601	2.062132199	3.149222734	0.001637054
Zfp937	422.7320165	6.493552451	1.801120034	3.605285782	0.00031181
Preb	376.9119814	6.481587584	1.773309963	3.655078763	0.000257103
Snora41	61.69703705	6.469376275	2.331170076	2.775162714	0.00551741
NA	43.69779657	6.456773232	1.343574307	4.805668879	1.54E-06
Cenpb	200.4076621	6.444197391	1.532610043	4.204720841	2.61E-05
9930012K11Rik	244.6907963	6.443548529	1.781780024	3.616354681	0.000298781
Smarcd1	259.5568882	6.442306825	1.394373985	4.620214443	3.83E-06
Klhl12	285.1709689	6.439354972	1.085226894	5.933648539	2.96E-09
St3gal1	222.30008	6.43366544	2.088350643	3.080740038	0.002064868
Gucy1b1	146.0241663	6.426940459	1.858477106	3.45817575	0.000543846
Lrrc1	152.2829311	6.42208885	2.036407461	3.153636477	0.001612498
Dok2	968.7730279	6.420949631	1.924275922	3.336813373	0.000847448
Patl1	636.4570456	6.419056127	1.3629266	4.709759224	2.48E-06
Eef1akmt4	400.251515	6.417742344	1.234751444	5.197598572	2.02E-07
Zbtb24	151.8914131	6.417004906	2.074362816	3.093482422	0.001978223
Paxx	104.6479651	6.415550231	1.988209681	3.226797601	0.00125184
Clec14a	323.9282404	6.402302113	1.968520745	3.252341703	0.001144583
Mfsd13a	90.18463477	6.396397855	1.613255398	3.964900946	7.34E-05
Kat6b	436.9899046	6.395158857	1.353500292	4.724903936	2.30E-06
Ankzf1	261.6549654	6.390447145	1.97600105	3.234030238	0.001220565
C030013G03Rik	228.5142789	6.390173223	1.80107777	3.547971848	0.00038821
Map3k4	236.9793653	6.388737497	2.139367468	2.986274024	0.002823995
Tsen54	87.63967134	6.38792463	1.880156861	3.397548769	0.000679925
Mtmr3	768.2325852	6.384454071	0.632755592	10.08992121	6.12E-24
Gm12932	158.9796827	6.384072117	1.482016197	4.30769389	1.65E-05
Etohd2	224.8482525	6.375745113	1.522106709	4.188763558	2.80E-05
Gm14302	10.96848425	6.373853466	1.834461891	3.474508517	0.00051179
Arxes2	219.1277911	6.371767223	2.151709408	2.961258244	0.003063849
Ddrgk1	478.6022896	6.370121407	1.074422251	5.928880752	3.05E-09
Acd	451.4114352	6.368193283	1.13710645	5.600349277	2.14E-08
NA	77.68189792	6.359656215	1.812110364	3.509530293	0.000448899
Otud7b	245.9659781	6.350755964	1.333535899	4.762343457	1.91E-06
Pla2g6	83.12278394	6.348633833	2.366670961	2.68251647	0.007307055
Susd3	142.3690831	6.348086894	1.440159231	4.407906264	1.04E-05
Zbtb8a	138.4381908	6.34806289	1.839157095	3.451615366	0.000557241
Mef2d	170.3927887	6.347173926	2.005639945	3.164662702	0.001552628
Serpina1b	222.0308939	6.338759612	1.735440529	3.652536348	0.000259663
Mtmr12	724.0324753	6.337119655	0.98572552	6.428888697	1.29E-10
Esyt2	696.7135079	6.336235927	0.797852714	7.941611043	2.00E-15

Gramd1b	97.55837283	6.333899255	1.920733059	3.297646816	0.000974987
Gstm3	21.14359842	6.331054826	1.694175135	3.736954165	0.000186263
Siah1b	194.0300575	6.327234529	2.258876228	2.801054104	0.005093598
Gtpbp2	394.4991233	6.324682704	1.072093205	5.899377662	3.65E-09
Gmip	487.5559683	6.324488318	0.736641878	8.585567159	9.04E-18
Ier2	115.341522	6.322541749	1.324428544	4.773788496	1.81E-06
NA	1220.064096	6.321357891	0.937121265	6.745506828	1.52E-11
Gm41818	10.50651315	6.314143976	2.088086436	3.023890136	0.00249547
Armc9	242.4106056	6.311693847	2.190737056	2.881082342	0.003963121
Zik1	690.6008864	6.306184595	1.551575486	4.064374986	4.82E-05
Senp8	934.1407202	6.302777564	1.166583538	5.402765733	6.56E-08
Jmjd7	10.4087173	6.297293953	2.213154287	2.845393107	0.004435664
Gm20045	193.5221289	6.295560688	1.698077534	3.707463625	0.000209345
Gsdmd	510.5856456	6.295471674	1.051540188	5.986905444	2.14E-09
Agfg1	1241.967107	6.288119509	0.639494888	9.832947261	8.12E-23
Chrnb1	104.9153527	6.283625657	2.304659584	2.726487547	0.006401237
Gm55122	371.1669961	6.281500702	1.300940765	4.828429448	1.38E-06
Ssx2ip	357.4205079	6.279414152	0.994197385	6.316063837	2.68E-10
Ubr4	353.8710889	6.279085296	1.653613541	3.797190298	0.000146345
NA	10.24246413	6.275512098	2.317190814	2.708241401	0.00676408
Mest	488.9916617	6.275301009	2.06306412	3.041738232	0.002352164
Gm16023	351.0331508	6.253969795	0.986002754	6.342750836	2.26E-10
Tie1	278.3294712	6.249981339	1.352740593	4.620236407	3.83E-06
Tmem94	202.6348276	6.248953549	1.919048243	3.256277466	0.001128834
2900026A02Rik	445.2566669	6.239467411	1.37241756	4.546333123	5.46E-06
Ptk2b	291.5734503	6.236709344	1.462731329	4.263742235	2.01E-05
Rfx8	140.5651872	6.235995243	2.28946534	2.723777965	0.006453988
Hivep3	638.7938568	6.234207791	1.811937753	3.440630221	0.000580361
Rdh10	181.8587585	6.23344627	2.300506238	2.709597639	0.006736487
Dennd4b	205.0118397	6.232300253	1.793993358	3.473981788	0.000512796
Stradb	547.7538812	6.231090822	1.056808712	5.896138775	3.72E-09
Dis3l2	899.5825372	6.230487632	1.477245829	4.217637654	2.47E-05
Zfp870	827.5725673	6.220444227	1.274917882	4.879094029	1.07E-06
Gaa	246.3769853	6.215912392	1.879657716	3.306938461	0.000943216
Apaf1	617.3405416	6.212501297	1.971919778	3.15048379	0.001630003
Lrch1	437.6737057	6.210876975	0.980411314	6.334970727	2.37E-10
C1rl	446.8617041	6.20974564	1.923803843	3.227847612	0.001247254
Tdrd7	623.9383931	6.208425216	1.832378399	3.388178566	0.000703584
Fam3a	140.7928095	6.207506835	1.711319125	3.627322773	0.000286375
Eps8	79.51464992	6.203245913	2.001219432	3.099733	0.001936952
Sppl2b	85.77169089	6.1999192	1.455012614	4.261075911	2.03E-05
Tom1l2	143.2160474	6.168603648	1.433290467	4.303805678	1.68E-05
Sox12	278.241486	6.167490957	1.241453724	4.967958802	6.77E-07
Ighv1-82	116.500529	6.163185756	1.797396112	3.428952425	0.000605916
Chl1	86.84568155	6.156807523	2.027683108	3.036375605	0.002394409
Foxk1	112.0089239	6.15468718	1.74313352	3.530817983	0.000414277
Mov10	348.3725073	6.15353443	0.705503929	8.722183076	2.73E-18
Ercc2	9.406157817	6.151708525	2.144915231	2.868042725	0.004130198
Rnf31	226.7204528	6.151349266	1.465378955	4.197787367	2.70E-05
Ydjc	41.45486898	6.14465222	2.111543707	2.910028431	0.003613959

Fam169b	76.4767689	6.141614444	2.163097141	2.839268902	0.004521703
Ube4a	632.3680879	6.139356282	1.719928353	3.569541876	0.000357606
Slc19a1	345.5676955	6.127935649	1.553795505	3.943849516	8.02E-05
Enpp4	590.2047942	6.126817156	0.964477166	6.352475074	2.12E-10
St3gal4	625.6769837	6.125128798	0.674544046	9.080398586	1.08E-19
D2hgdh	407.4510687	6.123679856	1.343261766	4.558813489	5.14E-06
9130019O22Rik	261.1886542	6.118858069	1.922797585	3.182268438	0.001461263
Ccdc14	726.5528783	6.111027001	1.851499067	3.300583355	0.00096484
Fads2	238.7574722	6.105732394	1.352630654	4.51396867	6.36E-06
Smpd4	1187.316724	6.099956144	0.61990236	9.840188607	7.56E-23
Gm17259	126.0619823	6.090408493	1.954014829	3.116869126	0.001827827
Hpse	204.7943022	6.087337269	1.126943928	5.401632785	6.60E-08
Rab5b	476.1520704	6.080456879	1.317201819	4.616192287	3.91E-06
NA	331.2448215	6.069864507	1.629657589	3.72462568	0.000195605
Borcs5	210.8669312	6.069636754	1.040649876	5.832544543	5.46E-09
Aig1	199.6328984	6.062812075	1.478428049	4.100850277	4.12E-05
Numb	343.5614605	6.061634519	1.345305447	4.505768213	6.61E-06
Ro60	1397.613507	6.05212167	1.528061269	3.960653799	7.47E-05
Snord83b	88.40261258	6.050493766	1.426321335	4.242027108	2.22E-05
Ttpal	437.1954552	6.04974182	2.090885189	2.893387859	0.003811104
Tubb6	550.1624155	6.049685812	1.305441073	4.634208266	3.58E-06
Gm3695	37.77045673	6.04836089	1.828926967	3.307054355	0.000942826
Cluh	513.1074614	6.044466003	1.821673031	3.318085024	0.000906369
Chd3	163.1518894	6.043180617	1.762917884	3.427942205	0.000608175
Rftn2	120.2517157	6.041158546	1.641374626	3.680548274	0.000232733
Pygo2	175.2131614	6.036253817	1.899701266	3.177475282	0.001485634
Prune1	386.48571	6.036171819	1.812224694	3.330807619	0.000865944
Zfp692	185.5463788	6.032539041	2.116009418	2.850903681	0.004359517
Ypel3	34.42965734	6.032234789	1.915669377	3.148891381	0.001638911
Gstm2-ps1	33.35236299	6.031211176	1.505126815	4.007111637	6.15E-05
Aak1	286.8083818	6.030171855	2.087719737	2.888401037	0.003872058
Zcwpw1	992.8752764	6.02567399	1.434857382	4.199493321	2.68E-05
Cd82	430.6678913	6.012012747	2.125992181	2.827862115	0.004685998
Cars2	506.5349611	6.002726764	1.824361848	3.290315883	0.00100075
Heatr5a	1761.160571	5.998534393	0.611399467	9.811154117	1.01E-22
Gm57150	16.4210103	5.995346721	1.647419742	3.639234476	0.00027345
Wsb2	245.9882178	5.991265928	1.793110909	3.341269019	0.000833964
Gm39792	208.5693475	5.988749551	1.808410159	3.311610212	0.000927607
Serpini1	236.9189891	5.986669383	2.049055058	2.921673266	0.003481566
Atxn2	376.8410981	5.979532154	1.709313855	3.4982061	0.000468399
Igfbp5	36.11730126	5.975406626	1.8648605	3.204211053	0.001354332
Jazf1	146.7848047	5.972044434	2.01812963	2.959197638	0.003084412
Mirt1	314.8820261	5.971476513	1.737823439	3.436181362	0.000589976
Zdhhc24	179.0575731	5.970491655	1.441551803	4.141711481	3.45E-05
Mfng	462.4202311	5.962722398	0.890071021	6.699153501	2.10E-11
Gm37646	56.82824567	5.960810568	1.583858358	3.763474518	0.000167569
Vldlr	154.4676405	5.954136777	1.980540372	3.006319317	0.002644312
Diaph2	597.0245422	5.951484397	0.757363906	7.858156889	3.90E-15
Rad54l2	247.7024209	5.947395832	1.153014229	5.158128738	2.49E-07
Tollip	470.0888327	5.947295149	1.212890369	4.903407021	9.42E-07

Zfp251	521.0789545	5.944938379	1.683873515	3.53051362	0.000414754
Gata2	231.8493537	5.944579478	1.9354535	3.07141426	0.002130473
Socs7	164.1620487	5.943556747	1.719277077	3.457009243	0.000546206
Tm4sf1	375.7763729	5.943296046	2.140203014	2.776977701	0.005486694
Tnfsf14	66.23966766	5.933842152	1.52809011	3.88317555	0.000103101
Rbpms	1260.03696	5.926173107	1.577068265	3.75771502	0.000171472
Stx16	577.0789933	5.91954134	1.295212111	4.570325811	4.87E-06
Slc35f5	82.6334167	5.913379304	1.944207134	3.041537706	0.002353731
Wdr27	23.9339265	5.91154434	1.913063587	3.090092969	0.002000939
Gramd4	337.6311765	5.898658645	1.673713909	3.524293259	0.000424614
Il11ra1	214.3586779	5.898514696	1.479199528	3.987639656	6.67E-05
Tada3	562.689435	5.896246734	0.921710357	6.397071153	1.58E-10
Ryk	196.0105772	5.895386545	2.051888305	2.873151785	0.004063988
Fbxo4	97.03842517	5.8920958	1.963781884	3.000381991	0.002696412
Gbe1	266.6843196	5.890257505	1.584598478	3.717192454	0.000201449
Ipmk	485.7527843	5.877874203	0.772468735	7.609206608	2.76E-14
Arhgap1	455.3492724	5.868390341	1.612851959	3.638517664	0.000274212
Xlr3a	71.14748086	5.868010524	1.825775464	3.213982573	0.001309076
Uckl1	607.7913169	5.865484643	1.029754801	5.696001259	1.23E-08
Ipo13	117.381184	5.86385499	2.022510262	2.899295544	0.003740022
0610009E02Rik	105.9602616	5.863165378	1.562237781	3.753055679	0.000174692
Serpinb6a	528.8907291	5.862752841	1.780410626	3.292921732	0.000991521
Pglyrp2	210.327963	5.861334803	1.593906781	3.67733852	0.00023568
Fancm	561.5760925	5.852847294	1.364268538	4.290099147	1.79E-05
Tsc1	886.8205694	5.821436634	1.36716891	4.258022977	2.06E-05
Traf4	123.3826494	5.817172127	1.986652988	2.928126937	0.003410108
Mapkapk5	60.1154114	5.812822176	1.592903775	3.649198569	0.00026306
Gm10286	211.6895428	5.810727279	1.966572583	2.954748444	0.003129241
Cdc14b	210.8832912	5.808107811	1.676358038	3.464717966	0.000530788
Atg2b	271.719683	5.805926171	1.020532095	5.689116684	1.28E-08
Map3k14	179.4827778	5.805583873	1.961771515	2.95935782	0.003082809
Gm56596	32.31948584	5.803253	1.577080961	3.679743237	0.000233469
Usp12	194.3218696	5.798697938	1.412272626	4.10593382	4.03E-05
Slc9a1	280.8387671	5.794626912	2.150030629	2.695136913	0.007035971
Mast3	297.4502721	5.791488562	1.158951738	4.997178375	5.82E-07
NA	679.8637789	5.789759271	1.232654374	4.696985136	2.64E-06
Hook2	172.4653261	5.772456465	1.517234416	3.80459104	0.000142039
Zfp976	530.8838909	5.770524272	1.360296114	4.242108916	2.21E-05
Manea	412.4606409	5.769506627	1.314642915	4.388649239	1.14E-05
Cox4i2	184.1096087	5.766731695	1.450458247	3.975799861	7.01E-05
Tmem42	38.59157694	5.766701821	2.125528878	2.713066794	0.006666367
Lcmt2	179.1662601	5.764416896	1.055320378	5.462243521	4.70E-08
Kctd18	189.7515322	5.76168115	1.180209997	4.881911833	1.05E-06
Btbd2	145.0502831	5.756255283	1.822047072	3.159224243	0.001581897
Sun1	678.9700394	5.755458889	1.826118658	3.151744201	0.001622984
Tns3	84.36114605	5.752424153	1.782080786	3.227925579	0.001246914
Mrtfa	122.8320705	5.746848818	1.572958908	3.653527623	0.000258662
Hspg2	31.40176859	5.74258092	2.065975414	2.779597899	0.005442625
Hecw2	133.019631	5.733648219	2.042654851	2.806958902	0.005001162
Slc25a16	151.185907	5.733056518	2.121147587	2.702808872	0.006875627

Rab27b	47.38274317	5.733045045	1.989078705	2.882261537	0.003948319
Cnot11	492.6964331	5.731438574	1.051619455	5.450107022	5.03E-08
Pik3r6	246.7687077	5.726604299	1.578419143	3.628063132	0.000285555
Nsun3	774.1465967	5.715757887	1.269777051	4.501386981	6.75E-06
Ankrd46	1389.005253	5.707134706	1.455810077	3.920246739	8.85E-05
Trbv12-2	84.42262072	5.705252359	1.474838233	3.868391958	0.000109555
Faap24	548.2666748	5.698984792	1.751429878	3.25390406	0.001138307
Arap1	198.1880262	5.68806057	1.321714049	4.303548543	1.68E-05
Sdc1	300.5636678	5.686306148	1.751722973	3.246121811	0.001169888
Alg12	339.0912001	5.681386004	1.453037712	3.910005885	9.23E-05
Btbd6	197.9450787	5.673798539	1.748761439	3.244466862	0.001176707
Acad10	97.46921435	5.670071688	1.966485408	2.883353045	0.003934662
Gm57403	153.2833094	5.65258988	1.200012989	4.71044058	2.47E-06
Abcd1	236.3466787	5.649701175	1.153920222	4.896093392	9.78E-07
Fam13b	342.9672134	5.647809638	1.16115644	4.863952388	1.15E-06
Gcn1	270.2077312	5.634962601	1.285620418	4.383068691	1.17E-05
Klhdc4	565.8881546	5.626274381	1.22124266	4.607007737	4.09E-06
Tbc1d17	148.2160421	5.620406756	1.059491403	5.304815819	1.13E-07
Nfatc1	709.7294355	5.617639709	0.890935889	6.3053243	2.88E-10
Ank	178.6477903	5.611714852	1.905339355	2.945257408	0.003226861
Cep95	622.6540415	5.605596235	1.49668788	3.745334154	0.000180154
Map7d2	171.6872795	5.591560937	1.324097113	4.222923591	2.41E-05
Golm2	582.1943723	5.589928234	0.588320665	9.501499043	2.07E-21
Crtc3	423.9841833	5.584483138	1.18240423	4.722989817	2.32E-06
Ermap	137.9352871	5.582154267	2.078712489	2.685390259	0.007244516
Ppox	200.6214292	5.576478569	1.557175068	3.581150691	0.000342084
Ninl	330.9793351	5.575548116	1.391737138	4.006179014	6.17E-05
Coq10b	163.7422565	5.573525519	1.069480648	5.211431855	1.87E-07
Pank2	410.1397344	5.573116248	1.17724951	4.734014497	2.20E-06
Klhl22	305.3084614	5.569464512	1.025100049	5.433093597	5.54E-08
Plxdc2	77.314	5.561272758	1.902430085	2.923246852	0.003464018
Xkr5	130.540058	5.555800915	1.222749312	4.543695803	5.53E-06
Nefh	403.0231156	5.555269058	1.794734772	3.095314776	0.001966041
Dusp23	112.2732026	5.549092423	1.534071311	3.617232383	0.00029777
Prdm11	166.1925155	5.547075404	1.282875455	4.323939149	1.53E-05
Fam83d	145.645031	5.535732696	1.94211092	2.850368966	0.004366854
Mylk	43.55837101	5.516963095	1.70442693	3.236843421	0.001208597
Dhrs13	84.89268051	5.512788184	1.940542187	2.84084944	0.004499355
Atp11a	164.6316184	5.511056089	1.662466324	3.314988105	0.00091647
NA	145.3299298	5.509891005	1.715204903	3.212380629	0.001316398
Lmbr1	99.36830158	5.502682917	1.651436235	3.332058968	0.00086206
Dhx33	629.6676526	5.500313764	1.772026635	3.103967885	0.001909441
Nol4l	157.2234748	5.499585712	1.600590467	3.435973051	0.00059043
Smug1	215.9438062	5.476859649	2.036459049	2.689403282	0.007157989
Plet1	95.81918799	5.476645236	1.344191244	4.074305097	4.62E-05
Kbtbd8	768.6619176	5.475272407	0.725887275	7.542868699	4.60E-14
Robo3	181.7638809	5.474442268	1.123409659	4.87305964	1.10E-06
Tgfbr3	195.8534473	5.473290732	1.742024077	3.141914514	0.00167847
Iffo1	128.5012742	5.472788943	1.318246795	4.151566281	3.30E-05
Ecscr	1709.419516	5.47144951	2.025090939	2.701829041	0.006895921

Ddx51	440.808967	5.466475358	1.625592895	3.362757905	0.00077168
F11r	401.0079777	5.454073571	1.322279268	4.124751633	3.71E-05
Klhl23	1139.777124	5.446849891	1.836075106	2.966572485	0.003011394
Slc35c1	390.0804392	5.445542075	0.611632743	8.903287369	5.42E-19
B3glct	353.6284474	5.445149498	1.09867283	4.956115552	7.19E-07
Tal1	512.6779076	5.430679581	1.334423016	4.069683688	4.71E-05
Slc4a2	140.1708776	5.429175759	1.169115766	4.643830761	3.42E-06
Ralgapa1	2287.621617	5.429133634	1.483227787	3.660350542	0.00025187
Gm18648	16.92710942	5.418159606	2.033556355	2.664376423	0.00771312
Zfp119b	1104.828443	5.417605908	0.836141108	6.479296201	9.22E-11
Selenon	250.8211555	5.414377781	0.95895876	5.646100755	1.64E-08
Thada	735.2210222	5.413087677	1.565349991	3.458068615	0.000544063
Igsf3	197.9366486	5.411479295	1.090781633	4.961102323	7.01E-07
Stat2	394.6534642	5.409449223	0.724090591	7.470680173	7.98E-14
NA	22.38090643	5.404347698	1.862088954	2.902303719	0.003704292
Zfp346	139.7602278	5.400855631	1.955512185	2.761862428	0.005747269
Stx17	742.459065	5.395327603	1.959780222	2.753026866	0.005904705
Mettl27	127.3652488	5.395061288	1.222779295	4.412130065	1.02E-05
Plcb2	614.9531353	5.393036345	1.670918069	3.227588741	0.001248383
Foxj3	316.4086673	5.385095566	0.981296952	5.4877329	4.07E-08
Rnpc3	384.3351651	5.384408111	1.524178719	3.532661914	0.000411398
Cacna1d	77.5257447	5.379385013	1.931449183	2.785154825	0.005350217
Agap1	427.0666209	5.375730978	1.462821002	3.674906888	0.000237936
Pdss1	195.1706079	5.362682999	1.417777776	3.782456665	0.000155288
Rgs12	179.7217153	5.356394525	1.163585864	4.603351323	4.16E-06
B3gntl1	296.8271388	5.346771536	1.745667422	3.062880975	0.002192172
Epb41l3	314.9892134	5.344168004	1.940018001	2.754700215	0.005874593
Exd2	305.0070765	5.343060019	1.643590954	3.250845356	0.001150624
Map4k3	287.9804418	5.336521788	1.068053839	4.99649137	5.84E-07
Cenatac	136.7597791	5.328569382	1.85824145	2.867533377	0.004136852
Pard6g	241.8141077	5.313752068	1.811146324	2.933916492	0.003347142
Neu1	381.2214676	5.305447314	1.120929063	4.733080344	2.21E-06
Ttc41	293.1680781	5.300444258	1.469915494	3.60595169	0.000311011
Zdhhc5	78.13998819	5.298777459	1.719535668	3.081516456	0.002059491
Srp68	1154.497062	5.290322241	0.878527185	6.021808237	1.72E-09
Mon2	847.8295551	5.288884558	1.382442661	3.82575329	0.000130373
Bcorl1	65.92319769	5.287141503	1.465056612	3.608830852	0.00030758
Zfp61	364.6187781	5.286386356	1.724599682	3.065283156	0.00217464
Tfip11	124.8635776	5.283175864	1.551545536	3.405105258	0.000661386
Wiz	308.7738336	5.271747015	0.847054652	6.223620874	4.86E-10
Zfp212	154.5471558	5.260530839	1.632102814	3.22316143	0.001267841
Blcap	107.2600762	5.246148119	1.712235885	3.063916698	0.002184597
Itgav	643.9861193	5.245828498	1.043067027	5.029234325	4.92E-07
Kansl3	701.7034088	5.242408002	0.981395607	5.341788737	9.20E-08
Specc1l	1582.000352	5.240243539	1.635861722	3.203353601	0.001358371
Plekhm1	116.5264212	5.238583654	1.504684189	3.481517047	0.000498582
Grik5	39.74871548	5.235657021	1.922632618	2.723170809	0.006465862
Zfp592	250.2678837	5.227828154	1.437540839	3.636646705	0.00027621
Gm24095	33.58594663	5.222590979	1.791299752	2.91553157	0.003550832
Triobp	60.60159341	5.221705544	1.281313936	4.075274138	4.60E-05

Smo	400.7102231	5.196296849	0.889696859	5.840525117	5.20E-09
Dnase1l1	681.9706068	5.193794528	1.744269855	2.977632453	0.002904841
Dph2	192.937652	5.19342679	1.230372661	4.221019335	2.43E-05
Tmem154	2040.620704	5.191314372	0.752015377	6.903202424	5.08E-12
Prep	283.9789245	5.188645686	1.334889843	3.886946713	0.000101513
Tedc2	273.913916	5.178528862	1.791068315	2.891307283	0.003836428
Foxk2	943.6630109	5.171236615	1.440849607	3.589018999	0.000331925
Plekha1	114.6422084	5.165978256	1.923495509	2.685724105	0.007237282
NA	142.4991174	5.161660861	1.299510067	3.972005289	7.13E-05
Tusc3	772.6566645	5.159167259	1.046743543	4.928778678	8.27E-07
Krtcap3	18.78310855	5.147466912	1.847828201	2.785684789	0.005341479
Arhgap6	253.2557408	5.143811002	0.501093524	10.26517157	1.01E-24
Matn2	110.4431794	5.141668939	1.621109508	3.171697479	0.001515508
Txnrd2	179.1100673	5.136302318	1.575987365	3.259101203	0.001117658
G6pc3	1170.196013	5.134351771	0.812925745	6.315892691	2.69E-10
Prr3	209.8649124	5.13179237	1.59606029	3.215287294	0.00130314
Scly	113.0059698	5.12734214	1.697686361	3.020193987	0.002526128
Atxn1l	166.398235	5.117540928	1.897587458	2.696866965	0.006999523
Vmn2r84	68.85266194	5.114606796	1.46974839	3.479919986	0.000501564
Bcl9	429.4248867	5.101361593	1.291392577	3.950279477	7.81E-05
Pdlim1	175.8641243	5.090268169	1.769481247	2.876700829	0.004018564
Phlpp2	356.325226	5.072213698	1.372201698	3.696405349	0.000218674
D5Ertd579e	383.7261017	5.06787085	1.792790953	2.826805234	0.004701491
Cd1d2	345.139885	5.057376058	1.056390388	4.787412036	1.69E-06
Mtus2	91.76324881	5.054880705	1.774778173	2.84817606	0.004397059
Acbd6	180.5769532	5.051557751	1.707472487	2.958500233	0.0030914
Gramd1a	242.3677571	5.041179478	1.334452233	3.777714445	0.000158274
Gm43511	159.7725999	5.036908986	1.721182622	2.926423333	0.00342884
Mcat	239.5209431	5.004329002	1.446019754	3.460761161	0.00053865
Zfp229	60.38894891	4.997217095	1.849618531	2.70175553	0.006897446
Oaf	415.1847741	4.989471909	1.316345592	3.79039664	0.000150407
Tspan4	2859.94955	4.980702404	1.737187825	2.867106443	0.004142437
Orai2	367.742317	4.974422064	1.208247267	4.117056335	3.84E-05
Smyd4	531.981644	4.966287714	1.359468525	3.653109743	0.000259083
Ski	264.5945363	4.965808746	1.79970004	2.759242448	0.005793553
Snn	60.16145165	4.964534153	1.767364318	2.809004404	0.004969497
Ssh1	566.5995487	4.956749385	1.406304009	3.524664193	0.00042402
Irf2bp1	77.80136995	4.942647916	1.102761295	4.482065102	7.39E-06
Prrc2b	1193.390784	4.93765245	1.042439304	4.736633039	2.17E-06
Zc3h12c	584.7559256	4.937305868	1.639779687	3.010956841	0.002604259
Ushbp1	64.43347291	4.936173375	1.771741117	2.786057922	0.005335334
Setdb2	132.8130299	4.935240735	1.169984827	4.218209179	2.46E-05
Cmtm6	852.7604957	4.927836351	1.393536161	3.536209887	0.000405912
Runx1t1	68.65727344	4.925906136	1.419120951	3.471096761	0.000518337
Bap1	280.4313134	4.9082527	1.584216686	3.098220555	0.001946865
Sox13	114.231245	4.902303016	1.731790883	2.830770772	0.004643599
Zfp758	451.4537212	4.896323099	0.849946506	5.760742664	8.37E-09
Mfge8	211.6653748	4.893331271	1.489986415	3.28414489	0.001022923
Tbc1d24	145.2082203	4.893105971	1.654365166	2.957694027	0.003099496
Slc17a9	143.9498691	4.891337955	1.629386954	3.00194987	0.002682563

St6galnac4	313.6370519	4.891169874	0.611770962	7.995099758	1.29E-15
Acvr2a	740.8128793	4.887965968	1.626733989	3.004772753	0.002657793
Glipr1l1	80.76370264	4.880638706	1.579243359	3.090491835	0.001998253
Bcl7b	328.7528272	4.860974254	1.641515393	2.961272416	0.003063708
Tmt1a2	15.15692251	4.854534302	1.619079546	2.998329708	0.002714638
Pml	626.7529635	4.836142693	1.531090613	3.158626048	0.001585147
Slc22a3	1078.149095	4.817371268	1.559733882	3.088585382	0.002011119
NA	85.36285378	4.815560058	1.7326662	2.779277428	0.005447997
NA	1293.215481	4.814767952	1.649013291	2.919787231	0.003502704
Phactr4	157.27689	4.813032125	0.89117578	5.400766305	6.64E-08
NA	88.53770885	4.809462663	1.741478287	2.761712678	0.005749905
Ttc38	205.3236361	4.808424077	1.681944959	2.858847462	0.004251832
Rpap1	328.5314161	4.806650926	1.434058252	3.351782202	0.000802932
Igf2bp2	1345.358819	4.801436308	0.83177473	5.772520053	7.81E-09
Gm35315	874.4493831	4.796369025	1.029519564	4.658842037	3.18E-06
Dgat1	205.9484378	4.785024913	1.729580347	2.766581454	0.005664743
Tnfrsf1b	181.1319602	4.783633503	1.556932421	3.072473434	0.002122927
Rnf157	167.8779783	4.776957534	1.740213576	2.745040954	0.006050335
Cgrrf1	578.8103213	4.77152313	1.148107297	4.155990597	3.24E-05
Slc37a1	260.318393	4.769670868	1.452042705	3.284800681	0.001020545
Marchf8	148.7070495	4.761644962	1.58056539	3.012621302	0.002590019
Gm15545	259.671991	4.758090357	1.251322193	3.802450227	0.000143272
Tubgcp3	607.1795446	4.741769578	1.475680224	3.213277172	0.001312296
Fbxl4	211.967851	4.734449477	1.565758427	3.023741975	0.002496693
Ube3b	442.3888434	4.73219647	1.208639556	3.915308287	9.03E-05
Pskh1	175.693879	4.72121538	1.770732027	2.666250629	0.007670249
Acads	193.3901271	4.710902166	0.975271319	4.83035036	1.36E-06
Jup	69.30071424	4.710417166	1.346337472	3.498689789	0.00046755
Spg21	779.3539944	4.695264111	0.672883335	6.977827902	3.00E-12
Atp13a1	252.6025674	4.68744922	1.457090655	3.216992164	0.001295421
Gorab	1035.599002	4.674021593	1.155839969	4.043831083	5.26E-05
Zmym3	967.66488	4.663279599	0.932705007	4.999736856	5.74E-07
Lrrc14	83.91664154	4.661490881	1.486042628	3.136848698	0.001707742
Afg3l2	1001.949889	4.657810599	1.332733847	3.494929323	0.000474187
Mir1931	63.0051929	4.654461186	1.572999071	2.958972623	0.003086665
Arhgap21	229.6432961	4.653952602	1.082273908	4.30016151	1.71E-05
Ino80dos	842.7259803	4.652831172	1.616775756	2.877845709	0.004004009
Stimate	253.8458017	4.63870303	1.191401759	3.89348345	9.88E-05
NA	410.7256411	4.614682233	0.949801668	4.858574574	1.18E-06
Tmem39a	474.847495	4.61124533	1.388083927	3.32202199	0.000893677
Bckdhb	71.46180421	4.610325224	1.181099139	3.903419343	9.48E-05
Hus1	862.4844034	4.604911918	0.954302668	4.825420775	1.40E-06
NA	121.4455356	4.593510062	1.672911423	2.74581786	0.006036026
Ccm2l	51.89962952	4.580260984	1.273405695	3.596859197	0.000322083
Anks1	319.2212145	4.576896935	1.297214714	3.528249323	0.000418318
Casp7	1134.699194	4.569983507	1.100611004	4.152224075	3.29E-05
Cdk14	271.1107994	4.564984864	1.30610687	3.495108225	0.00047387
Ogfod1	686.2328042	4.544538819	1.568521767	2.8973387	0.003763432
Ecm1	93.41550731	4.541668662	1.282052756	3.542497483	0.000396357
Chpt1	110.3584117	4.536356562	1.43285648	3.165953203	0.001545757

Gfm2	1526.51426	4.515594673	0.69074895	6.537244357	6.27E-11
Acad12	59.79847567	4.514993614	1.680474943	2.686736648	0.007215382
Vps52	120.2633726	4.51408974	1.473109515	3.064327326	0.002181601
Zfp729a	902.6384596	4.501191168	1.690088396	2.663287424	0.007738129
Glrx2	495.877395	4.496085044	1.213829163	3.704050934	0.000212184
Osbpl2	367.6597972	4.493096398	1.44585272	3.107575438	0.001886288
Gstt2	1731.483024	4.483469228	1.265710854	3.542253915	0.000396723
Trbj2-7	61.42903687	4.463409969	1.582227766	2.820965517	0.004787935
Nos1ap	134.0342949	4.450318999	1.556050524	2.860009319	0.004236286
Zbtb34	301.9961066	4.445658748	1.307430915	3.400301077	0.000673117
Tmsb15b2	23.58251487	4.421105704	1.495663038	2.955950366	0.003117072
Gpr180	160.2896511	4.416095077	1.014090187	4.354736031	1.33E-05
Xk	183.5016682	4.406112512	1.615955434	2.726629968	0.006398475
Atp2a2	1931.590554	4.402073512	0.765428356	5.751124158	8.87E-09
Pitrm1	2925.578887	4.393507104	1.362728845	3.22405086	0.001263909
Akt1	354.534055	4.392146006	1.243065625	3.533317886	0.000410379
Map4	327.7279008	4.388255648	1.173862594	3.738304354	0.000185266
Lsm14b	143.3417806	4.384909846	1.414162786	3.100710817	0.001930567
Dlx1as	89.88291432	4.383753075	1.245288066	3.520272294	0.000431104
Katnip	109.7094923	4.372918945	1.608628288	2.718414799	0.006559555
NA	59.49048054	4.370919665	1.19160804	3.668085073	0.000244374
Sik2	461.2441974	4.369692826	1.033319148	4.228793045	2.35E-05
Nob1	2543.204148	4.337089307	1.254634078	3.456855973	0.000546517
Cog7	303.4202213	4.31721831	1.046611369	4.1249488	3.71E-05
Wdr4	505.4052562	4.307777131	0.80411684	5.357153233	8.45E-08
Inpp5k	1238.109226	4.305475175	1.006813107	4.276340014	1.90E-05
Zfp146	180.3348166	4.304764828	1.555159816	2.768053022	0.005639228
Rbp1	4779.129922	4.295472602	1.451026834	2.960298528	0.003073411
Pbx1	1926.211319	4.295392699	1.414886315	3.035857123	0.00239853
Zfp952	995.5948989	4.294793128	1.515737039	2.833468482	0.004604585
Tmem87a	310.3555416	4.293714739	0.452069038	9.497918182	2.14E-21
Mllt3	2820.008416	4.279697609	0.961696797	4.450152713	8.58E-06
Mcrip1	405.837394	4.274578711	0.803868132	5.317512337	1.05E-07
Gm3200	68.2937066	4.268122375	1.56523888	2.726818525	0.00639482
6330549D23Rik	142.9149311	4.268066998	1.071336885	3.983870112	6.78E-05
Mavs	719.7484	4.266494354	1.421197423	3.002042001	0.002681752
Rreb1	893.5019286	4.250620711	1.344707851	3.1609994	0.001572288
Mgrn1	148.738339	4.248483779	1.213996065	3.499586122	0.000465981
Gfer	305.8785964	4.248044154	0.932685177	4.554638865	5.25E-06
Banp	116.2514693	4.24775462	1.432486457	2.965301765	0.003023862
Zfp704	1582.257337	4.247560298	1.020330961	4.16292405	3.14E-05
Adck1	1068.155259	4.245416874	1.442638996	2.942813058	0.003252448
Kat2a	425.5397412	4.242092784	1.406837331	3.015339934	0.002566914
Rhof	90.31392947	4.213987565	1.4838284	2.839942654	0.004512164
Lrp5	37.8816391	4.205912267	1.517956948	2.770771775	0.005592361
Stk25	732.8477713	4.20326176	1.193116194	3.522927426	0.000426808
Sfmbt2	202.8058391	4.202811223	1.551451814	2.708953759	0.006749575
Abhd17c	1046.648233	4.181996379	1.24360409	3.362803656	0.000771552
Atr	1777.742775	4.181088432	0.872338413	4.792966088	1.64E-06
Pigq	204.8160146	4.160402202	1.45819446	2.853118919	0.004329242

Lrrc8d	232.3029954	4.144647361	1.254164092	3.304709	0.000950751
Map2k5	1165.927981	4.14019301	1.481369427	2.794841674	0.005192514
Sec22a	1940.204005	4.129345963	1.00153189	4.123029933	3.74E-05
Ppp6r2	218.4113075	4.117790282	1.045573418	3.938308119	8.21E-05
Dlgap4	44.70666392	4.11431108	1.195686002	3.440962822	0.000579648
NA	127.4767787	4.11396426	1.488707972	2.763446114	0.005719454
Emc10	124.691972	4.107175085	1.227833322	3.345059149	0.00082265
Slc9b2	262.2896888	4.100731973	1.177420963	3.482808698	0.000496183
Tmt1a	2046.030586	4.099721572	1.276152352	3.212564367	0.001315557
Txlna	965.7767646	4.098181024	1.15415256	3.550813963	0.000384042
Apoe	98.86580069	4.094771741	1.412864133	2.898206308	0.003753036
NA	30.36064264	4.078291485	1.480117191	2.755384175	0.005862326
Traf3	137.3713737	4.071083354	1.209333842	3.366385039	0.000761603
Gcnt2	4742.22806	4.065305818	0.979713353	4.149484955	3.33E-05
Gm14322	139.4794253	4.063583012	1.37985948	2.94492524	0.003230327
St6galnac3	1020.024641	4.048208914	0.945385076	4.282074062	1.85E-05
Mxd4	370.1622906	4.044913101	0.999409039	4.0473049	5.18E-05
Ppp4r1l-ps	197.4788603	4.03906056	0.921389298	4.383663417	1.17E-05
Vps18	261.0537752	4.037486345	0.934454233	4.320689234	1.56E-05
Ddx60	720.6778671	4.022286851	1.465039569	2.745514139	0.006041616
Lck	1651.05062	4.019878474	1.428077199	2.814888773	0.004879412
Peak1	588.6627405	4.013252872	1.321484089	3.036928635	0.00239002
Ttyh3	204.7555658	3.992225151	1.321645284	3.020647976	0.002522344
Cln3	986.0129059	3.968641959	1.098451111	3.612943644	0.000302741
Slc24a5	369.5973522	3.96364535	1.48088743	2.676533861	0.007438805
Cap1	312.7886007	3.95151231	1.165708243	3.38979529	0.000699448
Leng8	112.4745967	3.948812082	1.125671795	3.507960402	0.000451556
Crtc2	108.2894042	3.948282931	1.040386292	3.795016294	0.000147634
Tmem38b	716.2976137	3.945537117	1.432998817	2.753342898	0.005899007
Mob1b	360.9374164	3.937943996	1.275650102	3.08700951	0.002021811
Akap7	128.6362476	3.923690397	0.770081867	5.095160094	3.48E-07
Slc43a2	104.4784964	3.920793131	1.103282171	3.55375373	0.000379775
Pogk	884.7786558	3.916451226	0.978560619	4.002257143	6.27E-05
Tnfrsf1a	154.8137179	3.908708564	1.293437086	3.021954918	0.002511479
Josd1	561.5100353	3.905201435	1.29778368	3.009131255	0.002619959
Ncor2	77.18595934	3.900430079	1.283189772	3.039636196	0.002368641
Gm8712	13.75600212	3.877605432	1.309871464	2.960294607	0.00307345
Ylpm1	1925.392918	3.874717472	1.080005304	3.587683744	0.000333629
Senp1	1452.21479	3.852362917	0.640854682	6.01128934	1.84E-09
Ttc28	85.16157378	3.849783474	1.163607356	3.30849015	0.000938005
Pdk3	478.5457215	3.847519709	1.120432206	3.433960295	0.000594831
Gm12454	648.4998696	3.844337562	1.412037944	2.722545508	0.006478111
Alyref2	215.0642444	3.844250173	1.182111095	3.252021058	0.001145875
Rassf2	927.9421755	3.842551149	0.618010266	6.217617023	5.05E-10
Ptgs1	1163.054519	3.839676137	0.895900722	4.285827709	1.82E-05
Mboat1	113.5798887	3.835332472	1.266345636	3.028661657	0.002456396
Glb1	618.269201	3.832646936	1.323935214	2.894890095	0.003792913
Dbr1	812.1664624	3.8278027	1.195959997	3.200610981	0.001371365
Trim8	282.6577552	3.79711376	1.392007054	2.727797786	0.006375868
2510002D24Rik	293.4541588	3.796921416	0.848715169	4.473728711	7.69E-06

Zc3h7b	701.4197538	3.778205438	0.777008314	4.862503231	1.16E-06
Znrf1	1034.046784	3.742946542	0.7856234	4.764301242	1.90E-06
Gon4l	254.1497204	3.738767548	1.399752552	2.671020347	0.007562106
Asb7	786.8713978	3.736938318	1.250672778	2.987942477	0.002808624
Gm20257	290.0106317	3.735500525	1.056652641	3.535220924	0.000407434
Tti2	117.8221517	3.717036798	0.939484449	3.95646442	7.61E-05
Rab12	254.3818228	3.702393851	1.140024308	3.247644657	0.001163645
Bcl2l2	463.6375135	3.675109007	1.112201294	3.304355988	0.000951949
Gfpt1	736.3262624	3.66093507	0.41574597	8.805701879	1.30E-18
Mtss1	712.9355049	3.660634985	0.756202418	4.840813647	1.29E-06
Cbx6	143.092299	3.647301168	1.318031846	2.767232961	0.005653434
Sipa1l3	163.9902724	3.64551662	1.165363279	3.128223348	0.001758665
Mrgbp	767.7691819	3.633060851	1.245414219	2.917150611	0.003532452
Slx4ip	1021.568896	3.632183422	0.867905126	4.185000541	2.85E-05
Zfp574	80.75408521	3.62146602	1.227725508	2.94973591	0.003180457
Usp40	1141.929916	3.605402765	0.970647166	3.714431865	0.000203661
Gm8717	33.55884176	3.598679649	1.152000191	3.123853343	0.001784994
Acap1	718.0240228	3.594450696	1.333994875	2.694501128	0.007049409
Nfix	323.2746615	3.594193898	0.589486467	6.097161008	1.08E-09
Atg16l2	727.8888658	3.586910648	1.158269419	3.096784383	0.001956321
Pced1b	321.8509065	3.584668973	1.292286514	2.773896449	0.005538931
Gna12	346.4278737	3.581609677	1.1125511	3.21927656	0.001285145
4930447C04Rik	611.9145554	3.561470058	0.817064541	4.358860138	1.31E-05
Zw10	2305.578081	3.538077289	0.866706986	4.082206958	4.46E-05
Pde4d	2572.910149	3.532711461	1.284879206	2.749450255	0.005969532
Men1	180.0893114	3.519963524	1.211748058	2.904864175	0.003674125
Khnyn	272.3322149	3.516576076	1.05259689	3.340857367	0.000835201
NA	104.5759432	3.476500074	1.279792317	2.716456435	0.006598488
Pik3ip1	128.5765798	3.467136845	1.260986935	2.749542243	0.005967857
Naga	645.3160508	3.454556385	1.282658978	2.693277358	0.007075338
Ano6	880.9068526	3.452079625	0.437337232	7.893404387	2.94E-15
Mprip	877.885321	3.447864175	1.165063723	2.959378192	0.003082605
Ctbs	138.9935336	3.447173388	1.173216972	2.938223254	0.003300992
Zfp1007	696.3687305	3.442475596	1.051094435	3.275134452	0.001056117
U2af2	115.2967985	3.441036171	0.710374003	4.84397818	1.27E-06
Fgd4	1623.900619	3.43988152	1.03874276	3.311581705	0.000927701
Zfp97	932.9552344	3.439272721	1.030028213	3.339008269	0.000840781
Sco1	1011.290998	3.426493923	0.836233855	4.097530736	4.18E-05
Mad1l1	1285.090121	3.41253983	1.212588779	2.814259781	0.00488897
Tut1	697.7746039	3.401730117	1.016263594	3.347291134	0.000816055
AU022252	399.0118704	3.389811241	1.263547968	2.682772104	0.007301473
Rbsn	683.5675883	3.388489464	1.225597277	2.764765823	0.005696367
Arhgap26	597.1486471	3.381172196	0.912928954	3.703653149	0.000212517
Gm15564	35.36130488	3.377408155	1.189461702	2.83944254	0.004519243
Mafg	360.4964345	3.372522555	1.120624381	3.009503107	0.002616754
Zfp637	1137.042732	3.371030916	1.156460769	2.914954839	0.0035574
1810014B01Rik	787.8307291	3.355060604	0.782566008	4.287255735	1.81E-05
Ptp4a3	841.875947	3.353583155	1.174332826	2.855734832	0.004293736
Adipor1	676.8346667	3.340689262	0.915425493	3.649329508	0.000262926
NA	796.2670011	3.333974734	1.022776298	3.25973015	0.001115183

Styx-ps	49.75060436	3.305585046	1.092703864	3.025142634	0.002485159
Med25	105.2056173	3.294294653	0.776033319	4.2450428	2.19E-05
Gtf3c1	117.8737927	3.278434017	0.846654572	3.872221478	0.000107848
Yrdc	195.2123802	3.261782385	1.224548639	2.663660944	0.007729543
Klhl20	566.9695382	3.257939054	1.068142743	3.050097074	0.002287674
Fgd2	235.6270426	3.25410908	1.168035223	2.785968279	0.005336809
Maged2	5140.268741	3.244898221	1.19755441	2.709604002	0.006736358
Hcfc1	1614.875413	3.242310331	1.109664909	2.921882367	0.003479229
Bak1	168.1750456	3.221135189	0.944582683	3.410114589	0.000649356
Gclm	536.0485661	3.200177073	0.697807214	4.586047564	4.52E-06
Ikzf2	5942.60526	3.194972486	0.32178138	9.929016057	3.11E-23
Car2	23373.72644	3.19459286	0.534857467	5.972792864	2.33E-09
Lratd2	777.3428693	3.187874588	1.19123158	2.676116585	0.007448073
Dpm1	427.1889322	3.147308296	0.957977328	3.285368248	0.001018492
Lcor	1850.630506	3.13806581	1.064899683	2.946818241	0.003210619
Tfcp2	177.2426542	3.134900729	1.059222525	2.959624304	0.003080144
Sass6	1653.874659	3.132379327	1.061398703	2.951180664	0.003165617
Irf2	2924.239052	3.122738096	1.021076035	3.058281645	0.002226102
Abcc5	286.5371088	3.110103177	0.91311574	3.406033915	0.00065914
Vps35l	3459.737364	3.1097727	0.791747572	3.927732535	8.58E-05
Ctla2a	6813.417843	3.103197094	0.803930142	3.86003327	0.000113372
NA	120.4394299	3.101195916	0.688677198	4.503119786	6.70E-06
Zfp64	307.5362279	3.099444782	1.058021078	2.929473567	0.003395367
Lgals9	10855.61253	3.099154478	0.709123363	4.370402443	1.24E-05
Depdc1b	122.024767	3.082326177	1.014785308	3.037417029	0.002386151
Mau2	2504.926121	3.073384906	0.680961446	4.513302365	6.38E-06
Snf8	803.1470932	3.064374467	1.093490131	2.802379629	0.005072714
Prkaca	511.3766211	3.056559353	0.795799583	3.840865737	0.000122601
Lin9	3018.9159	3.050259767	0.742784066	4.106522888	4.02E-05
Bbs4	1098.118225	3.045638864	0.77061679	3.952209327	7.74E-05
Irag2	4675.271565	3.036289537	1.064857112	2.851358649	0.004353284
Zfp397	1633.943092	3.012669306	1.07317371	2.807252245	0.00499661
Rnf114	494.1921855	2.987593219	0.499193126	5.984844464	2.17E-09
Ccdc88b	70.98179532	2.982384308	1.112321608	2.681224824	0.007335322
Trp53	3713.900511	2.981330451	1.039942229	2.866823143	0.004146146
Lpin2	1320.113611	2.980892115	0.670030898	4.448887545	8.63E-06
Xpc	1721.413587	2.960948241	1.063049868	2.785333342	0.005347272
Cnot3	272.2702064	2.955281893	1.04303645	2.83334479	0.004606367
Cog1	273.5617487	2.953833467	1.103441474	2.67692808	0.007430058
Rsph3b	41.60093445	2.951632715	1.084649832	2.721277068	0.006503023
Fndc3a	2949.265172	2.951379785	0.672725448	4.387198066	1.15E-05
Trim11	160.2387027	2.920485709	0.933859052	3.127330299	0.001764016
Chmp6	104.5801781	2.919920701	0.985962393	2.961492974	0.003061515
Dnajc25	561.0245274	2.919620263	0.97499402	2.994500688	0.002748944
Tigd2	495.8236225	2.903408279	0.784346527	3.701690745	0.000214168
Vav1	2108.874847	2.893126421	0.842290334	3.434832745	0.00059292
Cherp	513.3687512	2.879731985	0.773057559	3.725119754	0.000195223
Mettl25b	1040.658555	2.879373304	0.978395997	2.942952866	0.003250979
Ermardl1	134.7507106	2.871410971	0.7868525	3.649236637	0.000263021
Prkdc	2157.117398	2.867786514	0.934248053	3.069620006	0.002143313

Tmem209	2447.493797	2.866107099	0.569678352	5.031097091	4.88E-07
Hbp1	5807.46552	2.864637171	0.633225368	4.523882518	6.07E-06
Zfp955b	3643.58521	2.792275575	0.679585925	4.108789591	3.98E-05
Igf2bp1	2572.393116	2.78878466	0.795543467	3.505508843	0.000455735
Uba7	866.1164109	2.77333437	1.009011536	2.748565573	0.005985666
Dcp2	669.7807725	2.772853106	1.01462306	2.732889892	0.006278132
Spns1	214.7167467	2.768863141	1.020449631	2.713375612	0.006660157
Zfp746	368.8733053	2.76830137	1.001265302	2.76480306	0.005695717
Zfp935	307.932323	2.766161825	0.860842535	3.213319176	0.001312104
Parp10	439.3249226	2.754725178	0.905750716	3.041372344	0.002355024
C2cd5	1822.065569	2.743787964	0.933627629	2.938846153	0.003294366
Siae	1857.482491	2.734568731	0.478825807	5.710988613	1.12E-08
Sptlc2	1028.044091	2.719360654	1.005607878	2.704195852	0.006846992
Ndrg3	1558.287969	2.66298139	0.518884954	5.132122972	2.86E-07
Dock1	614.6310147	2.658054647	0.535112681	4.967280237	6.79E-07
Pip5k1a	1396.25121	2.657107403	0.954519361	2.783712423	0.005374066
Repin1	321.2848325	2.648937869	0.85741381	3.089450901	0.002005268
Trim23	3927.234174	2.571212275	0.940644821	2.733457112	0.006267328
Fus	1479.52973	2.549352779	0.700489649	3.639386798	0.000273288
Ogt	13370.92869	2.510194708	0.69465591	3.613580005	0.000301998
Per3	3643.721654	2.491321984	0.615903566	4.044987111	5.23E-05
Iqsec1	303.1531889	2.483082591	0.912393749	2.721503291	0.006498574
Atxn1	2838.549862	2.478835932	0.594693756	4.168256196	3.07E-05
Uhrf1	1460.02408	2.457226174	0.648744128	3.787666151	0.000152069
2500004C02Rik	2125.607591	2.448315077	0.628053583	3.898258274	9.69E-05
Tspan32	11489.34654	2.439119512	0.644082903	3.786965157	0.000152498
Yae1d1	1047.768591	2.433181673	0.724310458	3.359307665	0.00078138
Gm19590	5356.585516	2.432839388	0.434856031	5.59458583	2.21E-08
Slc12a6	1888.28909	2.426748278	0.838271965	2.894941473	0.003792292
Snx12	999.271952	2.421271886	0.805794188	3.004826693	0.002657322
Gm3815	21.478349	2.387366916	0.800304361	2.983073731	0.002853692
Dtx3l	586.7922266	2.385049201	0.558840398	4.267853951	1.97E-05
Tmem176a	2527.505498	2.351169341	0.84984007	2.766602123	0.005664384
Kdm5b	2813.443789	2.349989854	0.687413671	3.418596329	0.00062945
Alkbh8	3499.186736	2.328001029	0.694503506	3.352036394	0.000802195
Gbf1	559.4693256	2.318911459	0.720417096	3.218845683	0.001287077
Cd84	4679.162862	2.313324493	0.573466843	4.03392894	5.49E-05
Cnot6	4923.810871	2.31112529	0.802738201	2.879052333	0.003988721
Rp9	420.3670344	2.297622641	0.571360175	4.021320952	5.79E-05
Zfp512b	2529.183666	2.270278533	0.817752846	2.776240456	0.005499152
Tacc1	4625.80675	2.242427375	0.68018411	3.296794709	0.00097795
Cdkn2aip	782.3200837	2.233440938	0.816848551	2.734216685	0.006252888
Smarca2	1722.489351	2.228971797	0.349930031	6.36976424	1.89E-10
Nek9	1549.806101	2.199167291	0.470796797	4.671160269	3.00E-06
2010004M13Rik	1241.859483	2.190904569	0.520958429	4.205526669	2.60E-05
6820431F20Rik	703.4612819	2.18255713	0.517512021	4.217403735	2.47E-05
Cemip2	1323.894377	2.150804757	0.407040754	5.284003475	1.26E-07
Prpf6	2740.101526	2.136067605	0.213191547	10.01947607	1.25E-23
Rbm33	763.2860986	2.133369113	0.709066109	3.008702694	0.002623657
H2bc22	137.8630058	2.120735688	0.678575383	3.125276485	0.00177638

Arid1b	1426.724427	2.118866783	0.52134832	4.064205643	4.82E-05
Sypl1	4757.4255	2.095152119	0.481485764	4.351431079	1.35E-05
Dusp3	1561.429725	2.063482115	0.546979782	3.772501622	0.000161619
Kdsr	2261.132306	2.054271831	0.345646834	5.94326819	2.79E-09
Runx3	438.9584498	2.049869019	0.599627173	3.418572593	0.000629505
Sgms1	2100.932874	2.048300879	0.638523147	3.207872556	0.001337207
NA	66.43640253	2.047870056	0.751516904	2.724982027	0.006430499
Sel1l	1012.583586	2.047272292	0.744026714	2.751611271	0.005930287
Kit	5276.964686	2.046702191	0.647802776	3.15945264	0.001580658
H2-K1	2124.869838	2.023622458	0.756688128	2.674315061	0.007488207
Rock2	3746.447815	2.011122056	0.601195436	3.345205131	0.000822217
Dock2	9982.406396	1.988598513	0.676361295	2.940142386	0.003280615
Gm7805	566.8728499	1.988386176	0.673490193	2.952360994	0.00315354
6030458C11Rik	1982.024097	1.962979164	0.716068996	2.741326851	0.00611916
Fchsd2	3931.291948	1.957938917	0.671349752	2.916421598	0.003540717
Rnf6	3200.824873	1.946756928	0.623330297	3.123154672	0.001789237
Deaf1	432.3392505	1.940974988	0.36810112	5.272939648	1.34E-07
Zfp36l2	2846.271911	1.933686134	0.71977981	2.686496771	0.007220565
Aar2	1295.002057	1.918624644	0.636487184	3.014396349	0.002574912
Dtnbp1	3691.466182	1.918158205	0.647873854	2.960697047	0.003069437
Lpar1	856.5224436	1.893681873	0.502056205	3.771852346	0.00016204
Tkt	6181.355138	1.875231621	0.556431641	3.370102421	0.000751402
Apobec3	9026.720134	1.861295102	0.451195622	4.1252508	3.70E-05
Eri3	3657.81552	1.859341295	0.537166011	3.461390438	0.000537393
Pcbp2	7418.160819	1.841797133	0.346767111	5.311337427	1.09E-07
Retreg1	9879.751971	1.826618222	0.671158788	2.721588773	0.006496893
Zfp266	8360.670885	1.824604811	0.264414234	6.900554421	5.18E-12
Opa3	1366.4018	1.789489334	0.645832787	2.770824537	0.005591455
Gimap6	49338.43761	1.786869987	0.544460365	3.281910128	0.001031065
Mgst3	4176.976019	1.77298325	0.399748158	4.435250577	9.20E-06
Rbm22	5203.429723	1.732476992	0.590465945	2.934084525	0.003345331
Tcf12	2820.150039	1.705048356	0.563686883	3.024814676	0.002487855
Pkp4	1702.046213	1.70214292	0.592269854	2.873931383	0.00405397
Otud4	4534.8285	1.680186914	0.588326503	2.855874938	0.004291842
Sirt3	7411.41482	1.669288989	0.363984548	4.586153446	4.51E-06
Scd2	4941.600496	1.652420913	0.603095817	2.739897816	0.006145829
Mycbp2	4560.060391	1.648297536	0.510179456	3.230819112	0.00123436
Qrich1	2269.789792	1.643077817	0.447852273	3.668794194	0.000243697
Meis1	11513.66097	1.64150286	0.540076216	3.039391128	0.002370569
Tspan6	18229.81227	1.640300678	0.492125231	3.333096081	0.000858853
2010204K13Rik	1124.014527	1.639370273	0.581560866	2.818914352	0.004818637
Gm7890	1145.652509	1.636720438	0.486036569	3.367484144	0.000758574
Herc1	2975.458237	1.621862545	0.496585532	3.266028589	0.001090672
Cit	4481.90759	1.613483327	0.56447252	2.858391276	0.00425795
Ap2m1	6473.165167	1.60933524	0.559565282	2.876045551	0.004026916
Pds5a	7809.677959	1.608665508	0.512790708	3.137080063	0.001706395
Ptpn7	3853.79446	1.585295557	0.455888867	3.477372822	0.000506353
Hadhb	7209.706831	1.559468735	0.565304954	2.758632706	0.005804373
Khdc4	2895.443788	1.544811801	0.531615311	2.905882822	0.003662186
Gm57315	1553.50643	1.535293322	0.488847161	3.140640762	0.001685787

Cd1d1	4238.075309	1.51134906	0.455228523	3.319978832	0.000900243
Ipo5	9078.942238	1.498926741	0.365622517	4.099656532	4.14E-05
Srrm2	3336.874237	1.487678746	0.450752754	3.300431851	0.000965362
P2rx4	551.6455785	1.485490705	0.527419069	2.816528243	0.004854577
Kansl1	1060.247817	1.481321573	0.54073393	2.739464812	0.00615393
A630089N07Rik	6005.010248	1.472751247	0.45738476	3.219939481	0.001282177
Prpf19	636.4030154	1.463185972	0.402407544	3.636079872	0.000276819
B020010K11Rik	936.8377881	1.4583932	0.52239171	2.791761762	0.005242194
Ppil4	6344.607781	1.455574741	0.392885757	3.704829491	0.000211533
R3hdm4	234.014793	1.453289329	0.525088743	2.767702313	0.005645299
Acadm	11742.87853	1.44955173	0.430579423	3.366514174	0.000761247
Cd63	5560.641316	1.435329194	0.465972403	3.080287985	0.002068005
Braf	3673.951888	1.434030972	0.515575871	2.781415992	0.005412234
Lin52	2018.294596	1.428777697	0.369787954	3.86377566	0.000111648
Zfp638	8503.387955	1.427034202	0.496938275	2.871652826	0.004083313
N4bp2l2	6602.17109	1.41003871	0.385339975	3.659206935	0.000252997
Zcchc7	4691.398818	1.396307316	0.49191363	2.838521299	0.004532309
Prim1	16560.51281	1.38687089	0.40372649	3.435174366	0.000592173
Gpatch4	2527.498663	1.359406967	0.448349335	3.032026279	0.002429181
Tsnax	1945.529609	1.311973789	0.324677431	4.040853058	5.33E-05
Smarcad1	2027.891782	1.307731869	0.403952825	3.237338094	0.001206504
Il2rg	29590.57589	1.299390443	0.356579558	3.644040756	0.000268391
Ccs	2845.63851	1.298455502	0.264465339	4.909737922	9.12E-07
Tor1b	2144.730577	1.28371654	0.41689701	3.079217431	0.002075451
Arap3	1588.272592	1.28209222	0.450285704	2.847286088	0.004409372
Tmx2	9686.671345	1.26874221	0.395036543	3.211708466	0.001319482
Dapp1	15844.47609	1.250601556	0.432662963	2.890475177	0.003846599
Rbm18	5632.077624	1.23542458	0.21829098	5.659531047	1.52E-08
Vps41	6611.297542	1.224142188	0.455602843	2.686862485	0.007212664
Gigyf2	3239.851162	1.202086129	0.446506613	2.692202297	0.007098188
Ppwd1	669.9579661	1.166902246	0.37640553	3.100119823	0.001934424
Ipo11	22384.14029	1.163268319	0.374353604	3.107405152	0.001887375
A730008H23Rik	2810.111284	1.152847661	0.415665612	2.773497799	0.005545722
Nr2c2	2703.422924	1.126183024	0.399759877	2.817148715	0.004845209
Srsf5	29293.65511	1.124691549	0.34887349	3.223780486	0.001265103
Gstz1	3897.459264	1.099028775	0.362751856	3.029698555	0.002447979
Ppp1r2	997.7517123	1.08315062	0.355872855	3.043644958	0.002337308
NA	1662.240679	0.965673389	0.36060725	2.677908971	0.007408335
ND4L	222.1223657	0.897387816	0.334423183	2.683389977	0.007287995
Tbl1xr1	2584.527877	0.773070707	0.28849789	2.67964077	0.007370121
Fbxo46	2032.152136	0.635069624	0.221731031	2.864144098	0.004181377

padj
8.83E-15
4.03E-21
2.46E-14
9.69E-23
8.69E-14
7.33E-22
6.86E-15
3.59E-16
0.00045603
2.76E-09
1.27E-16
1.32E-13
1.54E-15
3.40E-15
4.95E-13
1.80E-14
0.000125078
1.19E-12
0.000237187
1.93E-09
1.47E-13
2.75E-18
1.52E-12
7.02E-13
5.17E-17
0.000152803
1.87E-09
7.00E-06
1.70E-14
1.80E-17
1.01E-11
2.75E-18
1.14E-14
1.31E-10
4.06E-18
5.25E-13
4.18E-05
2.82E-15
0.000224425
1.30E-11
0.000362183
3.43E-12
1.22E-15
5.34E-07
0.000333163
2.17E-17
1.18E-09
3.26E-14

0.000512666
1.64E-14
0.00026508
8.15E-10
0.000300741
4.06E-13
0.000414332
0.000576727
3.36E-05
3.52E-13
7.34E-07
0.000385745
8.61E-11
4.45E-11
7.37E-12
6.23E-16
0.001525784
2.36E-12
1.48E-06
0.000390047
2.36E-12
7.50E-15
2.00E-17
1.61E-06
6.55E-06
8.61E-11
9.12E-07
1.21E-07
1.31E-16
7.27E-10
0.000407466
4.22E-09
8.70E-23
9.53E-12
1.65E-05
0.000422395
2.12E-10
4.77E-10
8.78E-09
5.15E-07
9.33E-09
0.000521322
9.99E-07
0.00066728
1.12E-05
0.000699924
1.15E-05
0.000900138
0.002523037
5.23E-06

4.55E-05
0.002838944
4.82E-07
3.96E-09
2.19E-14
0.000976924
0.000638971
0.000816197
0.001828239
0.000971131
0.000635819
0.001133816
0.000644598
5.53E-06
7.66E-10
8.31E-06
0.002695534
4.75E-13
0.000672928
3.23E-06
5.46E-11
7.53E-06
8.29E-07
3.30E-06
7.95E-06
0.001700296
2.88E-10
3.12E-05
0.001179745
0.000771925
2.76E-05
8.50E-13
0.001504703
0.001136527
0.000799464
3.29E-10
1.42E-08
0.003241841
1.80E-05
0.000937255
0.002632564
0.003918929
3.79E-32
0.001419602
0.006619961
0.002074673
7.70E-06
3.60E-15
1.26E-09
3.41E-16

3.83E-09
5.17E-11
3.12E-05
4.30E-10
0.000993057
0.001116793
0.003639738
0.000917306
6.46E-12
5.91E-07
0.002388389
1.88E-09
1.85E-05
2.76E-07
2.73E-05
2.00E-10
6.75E-08
1.67E-09
7.47E-06
3.93E-08
5.80E-12
0.001090486
2.38E-10
4.53E-09
0.003323419
0.001359057
3.96E-10
0.001549585
0.001441361
9.23E-12
0.001248774
3.40E-13
0.005221654
2.73E-05
0.001163549
0.001916883
2.21E-07
0.001234356
0.006066351
1.78E-08
7.53E-06
6.49E-07
0.006606868
0.004378381
0.00120536
0.001398089
0.006284238
0.003589908
0.009472645
1.78E-11

1.15E-06
0.000406993
0.00149506
7.42E-09
0.002156469
7.90E-05
0.000464437
0.007355139
1.10E-10
4.75E-07
0.001689116
2.76E-12
2.58E-06
1.01E-05
0.003323688
0.001902376
4.41E-10
0.002646367
5.36E-05
7.95E-09
7.27E-10
0.001479324
8.81E-08
0.001771771
0.006235074
0.001848585
0.002461248
1.01E-09
0.002317037
0.003583302
0.004267875
1.75E-08
6.09E-06
0.003719135
0.003892963
9.04E-09
3.39E-06
1.69E-06
0.002008977
0.000945302
0.00207993
0.004406909
0.00010892
1.62E-05
3.41E-05
0.002829797
0.001734178
0.002109396
0.00275431
0.007152009

0.000635819
0.006633685
1.96E-05
0.000329052
0.001771771
1.25E-07
0.004688818
9.44E-05
1.91E-09
0.004597238
3.17E-19
0.001848585
1.70E-10
0.00022319
0.002695534
7.69E-17
0.001793638
0.002226447
0.004374296
0.002994425
0.003985094
1.06E-06
0.003240783
8.10E-13
0.002407709
0.005690827
0.003079238
0.00352671
9.08E-14
5.33E-05
1.77E-06
0.00368123
3.57E-06
0.002542063
0.008281003
0.004020143
0.002246567
0.000206976
1.59E-06
2.12E-07
0.002884698
0.001498518
0.002483249
0.01153196
4.48E-06
0.00279152
0.00283283
7.24E-05
1.66E-05
1.75E-09

0.002337906
0.00105238
0.002332778
3.19E-12
6.01E-06
3.33E-07
0.003222149
0.003826831
2.44E-07
2.41E-09
2.87E-05
0.000489343
0.002646367
0.00648408
0.004077484
0.002523037
0.003323419
0.010424764
8.11E-13
0.001011412
0.000521322
1.26E-05
0.005426027
0.002483249
0.002516591
3.12E-05
0.007105217
3.58E-05
4.55E-05
9.26E-06
0.006560375
1.69E-07
0.002714498
6.09E-05
0.000802789
0.000478172
0.008023939
0.002994425
0.002993479
2.56E-05
0.002756036
0.011914214
0.016414819
0.000951104
0.006570462
0.000158564
1.14E-10
0.013220089
0.000959582
0.009185576

0.003929339
5.43E-05
3.68E-08
0.004168295
0.006024036
8.87E-15
1.55E-08
0.004836356
6.98E-10
3.57E-05
7.50E-15
0.000160433
2.80E-05
0.009011175
2.13E-09
0.000124904
0.005447316
0.004194479
0.00370563
0.004841231
0.000441885
0.015539565
0.006353852
2.65E-05
3.61E-08
0.006917597
0.000151676
0.003377505
9.83E-11
0.009704721
0.000230624
0.003518722
0.005057672
0.007431151
0.005510463
0.004597397
0.004359072
7.97E-07
0.008677543
4.05E-05
0.017906486
0.000619352
0.003396874
1.23E-05
0.003647436
0.007112392
9.23E-12
0.00422025
0.019907341
0.003586628

0.003492129
2.02E-16
0.000108279
5.08E-05
0.003611648
0.000141015
0.005403075
1.24E-07
0.000642505
1.74E-08
0.00029125
0.005894294
0.003883061
0.004699441
0.0052303
0.005856407
0.003968658
0.003577396
3.14E-12
1.05E-09
7.29E-08
0.008281003
0.004283554
0.001115862
0.004167994
1.65E-08
6.48E-14
1.45E-07
0.006246037
0.004112796
3.97E-05
0.0075716
0.003870699
3.62E-05
0.007722285
0.008281003
0.004529701
0.006235074
0.017158449
7.27E-10
0.006149733
0.006387545
0.005269712
0.000244878
2.14E-09
0.009366479
9.27E-17
4.26E-06
2.43E-05
5.14E-06

0.007912011
0.000545167
0.010300445
0.00535734
2.08E-08
0.005610638
0.01704934
0.000644598
0.000379879
0.00460033
0.00486297
7.30E-05
0.017549811
0.016520018
1.29E-05
0.009795347
0.004065678
0.004809934
0.015074795
0.009882327
0.006697846
0.001391282
0.004990254
5.74E-05
0.004699441
3.57E-05
0.012775962
3.07E-06
0.021498964
4.64E-09
0.007451584
0.02746802
0.01244731
0.016635996
0.006908796
0.000269477
0.014827074
7.84E-05
1.45E-07
4.45E-07
1.92E-06
0.013717763
0.013486957
0.004943179
0.005726311
0.009867289
0.002427401
0.005289587
0.002219162
0.009602939

0.006283967
0.000267842
0.000218081
6.85E-05
0.006641073
0.001575752
5.05E-06
0.006885722
1.10E-06
0.01934391
0.000266254
0.02746462
0.013486957
0.006195942
0.00821943
0.000600518
0.01036827
0.006885722
0.00774749
9.59E-05
0.016776734
0.006119697
0.023678839
6.26E-05
0.000548245
0.010447057
3.52E-13
0.006235074
0.01983627
0.008519808
9.33E-09
3.48E-10
2.47E-07
0.006110221
0.000131982
0.006432391
0.011157367
2.45E-05
0.00724274
0.013499881
0.024843746
1.13E-05
2.81E-07
0.006576425
0.02186593
1.48E-09
2.38E-09
0.007229313
2.77E-05
0.013599638

0.000389955
0.007635666
1.31E-06
0.022220332
0.001706018
0.030250784
0.0010899
0.025885722
6.43E-07
0.011753627
0.000266663
0.002600278
0.028521903
0.001765914
2.32E-06
0.007229313
0.010477653
0.008559401
9.95E-05
4.89E-06
0.008743107
0.011898681
5.31E-07
0.007127697
0.010685071
8.44E-08
8.11E-13
2.54E-06
0.011026475
0.001244846
0.018566515
9.27E-17
2.39E-05
0.018497343
0.009783823
0.035343443
0.011520928
0.01538533
0.002941589
0.02051263
0.008559401
0.002477145
0.011924308
0.00066728
0.008580092
6.25E-06
0.011342751
0.009537297
0.012774519
0.012956018

6.35E-07
0.010702389
6.22E-05
0.000556845
1.27E-05
0.021590144
3.16E-08
0.000346018
0.032876266
0.036517086
9.29E-05
4.53E-06
0.000216634
0.011787097
0.031151175
0.002908235
0.01333718
0.010162027
0.010130547
0.023433934
5.27E-06
0.012140682
0.028817151
0.015852709
9.69E-06
0.041502641
0.010563083
7.29E-08
0.000206976
0.01144448
0.013885557
0.013066231
0.002973942
0.015535939
0.009867576
6.19E-07
0.010173585
0.012025061
0.000285862
0.000166607
2.57E-08
0.004841231
0.000644901
0.032349123
0.013396892
0.013149038
0.033621719
0.023852789
0.021076662
0.014210522

0.015749757
1.81E-09
0.012057857
0.015520928
0.001727323
0.010447057
0.035790266
0.02294069
0.035622276
0.000366042
0.010661983
7.03E-05
0.000614827
0.021858124
0.018175285
1.79E-05
1.11E-06
6.86E-10
0.000361374
6.48E-07
0.01542089
0.0119227
0.024654157
0.01131962
0.042574392
5.28E-06
0.011846509
0.0424105
0.017906486
0.031311068
0.012025061
0.014706098
0.032349123
0.016650576
0.000328601
0.001000528
0.028013358
0.035201615
0.01569668
0.017437298
2.73E-05
0.038004561
1.54E-11
6.21E-05
0.032661883
0.013083417
0.016029924
0.027445899
0.013445251
2.57E-08

4.49E-05
8.67E-05
1.99E-07
0.000106581
0.035135858
0.01549249
0.021534056
0.000816197
0.000184482
0.01773202
0.047802907
0.000493602
0.004289414
0.000128114
0.000540094
0.01934173
0.037659013
0.00154096
6.21E-06
0.016029924
0.022645825
0.021936425
0.013811557
0.000286485
0.041441465
0.026079833
0.003738755
0.018530069
0.041130812
0.000104443
0.049776465
0.023111169
0.017038028
4.26E-11
0.000464613
0.031765022
0.015710088
2.88E-06
5.10E-11
0.003192709
0.029702765
0.000192451
0.009735601
0.009004694
0.015838341
0.04668798
1.52E-12
0.004072312
0.00359795
1.02E-07

0.000387066
0.023196552
0.041853934
0.018162372
0.016212422
0.027814643
2.45E-05
0.039537598
0.040671685
1.47E-07
0.020649906
0.039308821
0.000706585
0.02130536
0.029983994
0.023448815
3.78E-05
0.024239316
8.14E-06
0.021837516
0.017878825
0.000211627
7.69E-08
7.97E-07
0.017796196
0.021590144
7.50E-15
0.003178817
0.024703524
0.003766003
0.006511668
5.23E-10
0.002963819
0.044162468
0.015035636
2.84E-05
0.002865495
0.042346361
0.000237187
0.022146002
0.04896708
0.001005414
0.026340191
0.000933814
0.031122562
0.00243719
0.023052648
3.88E-07
0.041992526
0.028289913

2.14E-07
0.00152234
0.020029101
0.018925551
0.000168413
0.022146002
8.08E-06
0.003377505
1.13E-06
0.005394431
0.042174258
8.83E-05
0.003591004
0.042275878
0.04748383
0.02307367
0.041387813
0.001691125
0.045670209
0.024763488
7.22E-06
0.017038028
0.034145749
2.47E-05
2.66E-05
0.024643625
0.039478484
0.028410796
9.50E-05
0.043857085
0.004237027
0.038144954
8.99E-05
0.030086371
0.000763376
0.004556232
0.00461841
0.024763488
0.000977289
0.002952141
0.042164106
0.029495857
0.000418473
0.000318202
7.72E-10
0.03916664
0.040080851
1.05E-05
0.02591372
0.018697402

0.003959688
0.026018869
0.025214555
0.01235039
1.27E-05
0.048962883
0.008317195
0.047223851
0.007665912
0.044162468
0.046060593
0.033821248
0.035383585
0.038760132
0.027369554
0.026929572
5.40E-06
0.028521903
0.040590163
0.014917747
0.028866023
2.38E-12
2.32E-06
0.039451332
0.002213449
0.000513059
0.027817103
0.04866511
0.027365206
1.95E-08
0.044309821
0.028406085
0.007409857
0.008419751
0.00205235
7.02E-11
0.039163503
0.002600278
3.43E-06
0.005751422
0.000381375
5.42E-13
0.007529326
0.03119505
6.32E-05
7.80E-06
0.039879958
0.028674253
0.028521903
0.001148944

0.001503549
0.001137499
8.61E-11
0.028505871
0.029069184
0.014917747
0.03142988
4.28E-05
1.35E-09
0.007988368
0.047517733
0.014100635
0.006003661
0.001228839
0.018715614
0.000472575
3.27E-12
0.044600754
0.044685022
0.031122562
5.90E-08
0.000748714
0.031163447
0.034067523
0.033764648
0.033338254
0.009688851
0.044162468
0.014100635
1.68E-05
0.035829373
9.42E-15
2.22E-25
0.031443514
0.000116621
0.013890142
0.01539704
7.00E-06
0.03870425
0.038822355
0.000747151
0.037147283
0.009689226
0.039716014
0.035670946
0.040989671
7.73E-09
0.038012255
0.006441722
0.002329437

0.000759716
1.08E-06
6.60E-07
0.012336541
0.014425972
0.03142988
0.00107257
4.54E-09
0.000143773
0.001693636
0.041634717
0.036578662
7.17E-07
0.044600754
0.04455298
0.020328948
8.81E-05
0.008697285
0.01090599
2.62E-05
0.019295318
0.048719441
0.007466492
0.003446612
0.000160203
7.88E-07
0.038842268
5.75E-10
0.011330696
0.049661891
0.000355185
0.04850523
0.00535734
6.98E-10
0.039451332
0.018697402
0.009882327
0.009071442
2.54E-05
0.045895283
0.049352029
0.042988277
0.002504457
0.002120563
0.000271082
0.043859802
0.002219162
0.043749176
0.005411291
0.001453876

0.011753627
0.000128951
0.005568447
0.002328931
3.70E-05
0.018705931
0.048962883
0.002135481
0.044600754
0.00072291
0.046269604
0.012689042
0.000216156
3.89E-08
0.040671685
2.49E-07
0.000337022
0.000267296
0.002838621
0.018490369
0.001030829
0.049661891
0.013952072
0.002944631
0.000120845
1.67E-06
3.61E-09
0.001921383
0.003064441
0.000132762
1.48E-05
0.001242266
2.68E-05
2.47E-08
0.047569645
0.016062682
0.000748642
0.044600754
0.013842642
4.95E-05
0.000981337
0.019064006
4.89E-05
0.006885722
0.026948191
0.001177815
0.000286798
0.002743353
1.62E-06
0.015889291

0.004345805
1.05E-05
0.000173084
5.23E-05
0.017221351
0.006047317
8.10E-09
0.01569668
7.45E-10
7.18E-05
3.42E-06
0.000129261
0.002749619
2.31E-06
0.007375634
0.005113516
4.83E-10
4.26E-07
0.001824064
0.024391727
0.042349387
0.02264366
0.006765771
2.59E-06
6.91E-10
2.48E-14
1.63E-23
0.00855071
0.047580353
0.000808507
0.011711426
7.20E-05
0.003968658
0.003679721
0.000771925
0.001205324
0.020835223
0.006283967
5.14E-05
0.032025217
0.003759165
0.000976193
1.10E-06
9.78E-11
0.006195942
1.20E-11
2.78E-09
0.025465146
0.001454708
2.98E-13

0.020002205
0.044851126
0.011846509
0.009223556
1.99E-07
0.000372428
0.004428116
0.028708717
0.021883818
0.000653231
0.007357283
3.83E-05
0.011077917
0.039117918
5.94E-05
0.004457372
2.24E-06
0.000116621
0.00018599
0.001813863
0.02591372
0.041340848
0.040672799
0.004217
9.49E-05
0.007044849
0.000104132
0.006633685
0.031311068
7.17E-07
0.005276928
0.001479324
0.006195942
0.007717036
8.81E-12
0.000106581
0.00178635
0.00833515
7.33E-05
0.003909286
0.031720799
0.002934655
0.003213153
0.014469732
0.040934132
0.002773944
2.67E-09
0.000175237
0.000163074
4.32E-13

4.77E-05
0.000489343
0.047240765
0.049661891
1.87E-06
0.011766357
0.035512375
0.015057304
0.00243719
0.046269604
2.76E-11
0.046370196
0.044250333
7.85E-19
0.00437979
0.000537872
0.001855727
5.75E-10
0.033338254
3.49E-16
0.004463977
0.000854319
2.48E-08
0.001981495
1.44E-18
0.009504211
9.73E-06
0.007083885
8.86E-19
0.000104132
0.000327519
2.77E-06
3.61E-09
4.79E-11
0.013570213
0.000115697
0.005938964
0.000332973
1.34E-05
0.030947071
0.017629735
0.000388266
0.009735601
1.12E-13
1.12E-09
4.08E-06
0.001008855
0.03142988
1.18E-06
0.002660275

0.000711293
0.026200501
7.03E-07
0.002530267
0.002612139
0.006492668
0.010232602
0.010162027
0.01873473
0.043989836
4.55E-36
0.038285378
2.75E-18
0.004501117
0.012269588
0.004438713
0.017801392
0.047735958
0.000867848
0.000717965
0.000399775
0.000362191
0.038674467
0.002683931
0.045534922
4.75E-07
4.66E-14
0.018734361
0.008023939
1.96E-05
0.000740557
9.86E-05
0.037680343
0.004020143
0.0008497
0.007568441
0.004514972
0.017316997
4.43E-06
0.046370196
2.43E-08
0.002594602
1.59E-06
4.23E-08
4.45E-17
0.021681086
0.000394641
0.012652961
0.001870778
2.56E-14

0.004490227
0.046370196
0.047186045
0.015953426
0.009882327
0.012025061
0.047569645
0.001710129
1.46E-05
4.05E-05
0.015009198
0.00404642
0.003515166
0.039089131
4.51E-05
0.000523764
0.003925509
0.000100109
1.67E-07
0.018006188
0.006283967
0.014839192
0.008950191
6.83E-05
7.53E-06
0.017435443
0.012146783
0.011359145
0.001246818
6.37E-05
0.011929314
0.004818291
0.02307367
0.007451995
9.06E-21
0.000360804
0.000556708
0.006014346
0.024709377
1.70E-07
9.99E-07
0.005411291
5.43E-05
0.04781068
0.000241481
0.006391227
0.014386587
0.003533849
9.11E-09
3.56E-13

0.009970049
0.002717236
0.036736923
2.02E-07
2.73E-15
5.19E-05
1.30E-09
0.020969104
0.030108738
0.000874562
2.78E-06
0.03282303
0.002974835
1.24E-07
8.59E-20
0.043558416
4.08E-05
1.78E-08
0.002246567
0.045217037
0.020004874
1.55E-08
0.000100109
0.011240512
0.000135393
0.000419841
0.04381669
0.006590036
0.045134466
0.006021386
2.05E-07
0.000499901
3.30E-05
0.009735601
0.014953813
1.61E-08
0.012126126
0.007665912
0.003789417
0.017143122
0.000423687
0.000364395
2.25E-05
0.006812296
0.020271019
0.005077976
8.98E-16
0.031122562
0.000537872
0.028046063

0.033326463
0.004506445
0.00135226
1.46E-08
5.17E-17
0.000128887
0.013711612
0.009885276
0.00015572
8.59E-20
0.016343378
2.79E-06
0.000101888
0.002828444
2.90E-07
0.000764752
0.00016053
0.001266301
0.000457447
0.029193487
9.47E-05
0.009735601
0.009471139
0.006827328
0.003241841
0.013885557
0.009100097
0.032372805
0.015014848
0.001075797
0.029568628
0.000535279
0.034315321
0.010173585
9.95E-20
0.003657606
0.008826663
0.009626122
0.027290388
0.005584206
0.012896198
0.024763488
0.006668529
0.000661182
1.74E-09
0.002506254
0.021873922
6.34E-13
9.12E-06
2.95E-05

0.005079617
0.018490369
0.006287704
0.038927399
0.001673923
0.002549183
0.000123046
0.02000622
0.017593763
0.005178962
0.00115301
1.11E-08
0.030733132
0.022156368
0.002893167
3.78E-12
0.00366449
0.012562211
6.16E-07
0.028797973
0.002586665
0.010118158
0.003276729
0.000383807
0.00042891
0.026815437
0.003566964
0.02501872
0.006188484
6.35E-07
0.024763488
0.003249036
0.000750951
0.046634569
1.96E-05
7.20E-05
0.002195372
0.000457447
0.00026265
0.001203487
0.044725371
2.05E-06
3.26E-05
0.014602963
0.014917146
0.012126126
0.00352671
0.03876332
0.036175969
0.04587986

0.030051432
2.19E-06
0.003781949
0.000163074
0.001473344
0.001757516
0.01131962
0.000364395
0.011556056
0.001520128
0.011615668
0.029983994
6.82E-05
3.06E-05
3.53E-05
0.00026698
0.000105379
4.51E-06
1.89E-08
0.025660649
0.002646367
0.00049184
1.51E-18
6.42E-05
0.047569645
0.004359072
0.001078776
7.08E-06
6.16E-05
2.38E-06
0.027181617
0.000136869
0.0173384
0.003918929
0.000337211
0.032411031
0.011851455
0.033227904
0.009537297
0.012599813
0.009071442
0.01697064
0.006668568
0.047240765
0.000844712
6.19E-12
3.39E-05
0.015322509
0.000639972
0.045963311

0.008291921
0.000704538
0.024405969
2.06E-16
2.38E-05
0.00085846
9.09E-05
0.00345319
0.049829043
6.75E-09
7.84E-07
0.006283967
2.32E-05
1.01E-11
0.028567424
0.040159835
0.040989671
0.000237187
0.01212914
1.79E-06
0.005051056
0.038270852
0.003304991
0.002358309
0.000106689
0.018849881
0.04085726
0.011403811
1.96E-05
0.031151175
0.026376338
6.18E-05
0.004039596
0.017969877
1.02E-07
0.002032032
0.0040091
0.018742663
0.007297419
3.09E-08
0.012269588
0.018795661
1.68E-05
3.75E-06
0.012926348
0.005887165
0.043857085
0.00368123
0.027672193
0.000842243

2.78E-07
0.023619978
0.000494652
4.92E-10
0.001649949
0.029357101
0.004258933
0.047569645
0.001218588
2.66E-05
0.038245296
1.87E-21
0.014100635
0.011150444
1.78E-08
0.012513358
0.021168298
0.046476203
0.005912939
0.001317941
0.030438167
0.003074931
0.034411756
4.89E-05
0.032618866
0.024783087
0.002398737
0.02693413
0.006240843
0.045963311
0.002303077
0.031163447
0.00072291
0.003529207
0.040426553
0.036017297
0.00517598
0.000176833
6.09E-05
0.021675805
0.038230352
0.000499483
0.004996126
0.006066585
0.017210285
0.034055358
4.37E-07
0.010352989
0.024834545
0.022091611

2.46E-13
0.021936425
0.017580589
0.024709377
0.022269057
0.014623849
0.01764692
0.038782932
0.02741255
2.79E-06
0.040159835
0.031764316
0.008559401
4.10E-07
8.53E-05
0.039716014
0.01844597
0.041668514
0.000631846
0.010336442
0.021590144
0.002212127
0.012576805
0.020969104
0.00149542
0.049661891
4.05E-05
0.005578578
3.08E-10
0.01244731
0.000945302
1.94E-05
0.015513215
0.005635065
0.024763488
0.000368929
0.030357073
0.001613179
3.59E-05
0.009358322
0.001553477
4.13E-05
0.041628059
0.004157872
0.005114809
0.000638971
0.005635065
0.028918079
0.004898882
0.014349854

4.64E-09
0.047517733
0.018780792
0.04992526
0.003000767
0.016794692
0.004899319
0.034992573
0.031680142
0.007399289
0.024934896
0.000298894
0.043558416
4.59E-07
0.012247846
0.00504272
0.002708023
0.017107068
0.005240851
0.04426952
0.003372268
0.000481173
0.006287704
0.000704538
3.47E-06
0.000401885
0.039648646
0.024746368
0.020294304
0.033815805
1.51E-18
0.000203618
4.26E-06
0.043554783
0.001168735
0.022091611
0.014532379
0.005568447
0.000130809
0.02449361
0.000615387
0.025822515
0.021485751
0.033289297
0.039412702
0.00520144
0.045181262
0.008291921
4.77E-05
0.03222617

0.009795347
0.037377517
0.000708011
0.001380722
0.006586992
0.04002266
0.008725632
0.005868194
0.012599813
0.004774565
0.028866023
0.040791098
0.00821943
0.000643292
0.025674424
0.000393351
0.000935563
0.000266663
0.000341709
0.041634717
0.035512375
0.020245441
0.021148557
0.003963465
0.048479922
0.007635666
0.005427105
0.002262954
0.040988552
0.017724665
1.23E-05
0.004725482
0.001092949
0.021076662
0.021769772
0.020098833
0.024746368
0.00427339
1.08E-07
0.009705827
0.006697846
0.04389995
0.011364351
3.18E-08
0.000388438
0.020689339
0.029069184
0.013024893
0.043445687
0.000183576

3.55E-05
5.40E-05
0.049024565
0.022976407
0.005010698
0.001287231
0.01151744
0.009795347
4.48E-16
3.88E-05
0.039710815
0.015859044
0.027557961
0.000564498
0.025352847
0.002919269
0.016028142
0.04668798
6.58E-08
0.017273254
0.03916664
0.012372651
0.000293762
0.000818514
0.041340848
0.028423599
0.008833446
0.044471466
0.041340848
0.046832603
4.95E-13
0.024763488
0.026082066
0.010616377
3.83E-05
0.009626122
0.008886109
0.000771925
0.035555492
0.008680546
0.047802907
0.039879958
0.003002466
0.033324918
0.021755349
0.027708798
0.000387066
0.032012762
0.003566964
0.011134531

0.020907812
0.000453235
0.001734178
0.049908893
0.019546215
0.038230352
0.045134466
0.027286518
0.007191483
0.000115121
3.84E-20
1.34E-07
0.04850523
0.010329319
0.025545207
0.024763488
0.02526862
0.019108347
0.007278065
0.001436321
0.001803108
0.000162279
0.026713708
0.000280785
0.020224233
0.000155952
0.036621979
0.001921383
0.000749984
0.001308847
0.032349123
0.036160705
1.25E-07
0.047974429
0.031163447
0.000204493
0.038268273
0.033815805
0.048444241
0.043989836
0.000263997
0.015889291
0.024709377
0.022537995
0.003020171
0.00668649
0.002825669
0.025822515
0.003566964
0.018559083

1.67E-05
0.000149338
0.000746427
0.005468455
0.04139643
0.042851569
0.044724236
0.039879958
0.012576805
0.02000622
0.026057517
5.68E-07
0.045709382
1.03E-05
2.25E-05
0.038404341
0.017621371
0.042824334
0.003657606
0.003959688
0.000942568
0.043989836
0.000602764
0.002323721
0.001583451
0.002327881
0.008377912
1.03E-06
0.029069184
0.021936425
0.023267964
0.000414332
0.039716014
0.007018889
0.008559401
0.012383206
0.000976193
0.030256659
0.001017721
0.0389971
0.009986551
0.042745389
1.32E-08
8.06E-05
0.000522609
0.000499901
4.99E-06
1.68E-20
0.021788274
0.015978228

0.000874562
0.000302519
0.00243719
1.58E-07
0.007018889
0.012757751
0.04369729
0.041130812
0.014600625
0.048673342
0.008725632
0.025989302
0.02519939
0.042047515
0.027607897
0.016037065
5.23E-06
0.047517733
0.021528356
0.024740966
0.002440822
0.008139003
0.000704538
0.006235074
4.39E-06
0.043989836
4.98E-10
0.039412702
0.010414034
0.000216156
0.0263761
0.02091861
0.030673039
0.032012762
0.000115121
0.042174258
0.012025061
0.003366067
0.020103671
0.009051269
0.035182231
0.008210675
0.01091926
0.031777956
0.030483918
0.015510494
0.005959914
0.040482974
0.028375711
0.015370347

0.009413767
0.000767744
0.009885276
0.035374866
0.042205301
0.012352107
0.003686018
0.037680343
0.002994425
0.039672496
0.00821943
0.018022928
0.038640165
0.001781432
0.030863509
0.003465427
0.033338254
0.006683156
0.020506771
0.000954695
0.011846509
0.003611648
2.87E-05
0.018077182
0.032661883
0.012621169
0.029419728
7.30E-07
0.047517733
0.046941877
0.017130988
0.016794692
0.039177242
0.035341381
0.012251397
0.020641931
0.019907341
0.048345181
0.047770404
0.048159478
0.031364567
